# The complement cascade in Alzheimer’s disease: modern implications of an ancient immune protagonist

**DOI:** 10.1186/s13024-025-00916-y

**Published:** 2025-12-15

**Authors:** Maria-Tzousi Papavergi, Praveen Bathini, Brijendra Singh, Cynthia A. Lemere

**Affiliations:** 1 Ann Romney Center for Neurologic Diseases, Brigham and Women’s Hospital, Harvard Medical School, Boston, MA 02115 USA; 2 Mental Health and Neuroscience Research Institute (MHeNs), Department of Psychiatry and Neuropsychology, Maastricht University, Maastricht, 6200 MD The Netherlands

**Keywords:** Complement system, Alzheimer’s disease, Cerebral amyloid angiopathy, Genetic risk factors, Epigenetic regulation, Gut-brain axis, Biomarkers, Complement-targeted therapies

## Abstract

The complement system, a critical arm of innate immunity, has emerged as a key contributor to the pathogenesis of Alzheimer’s disease (AD). While complement-mediated synaptic pruning is essential during brain development, its reactivation in the aging and diseased brain can promote neurodegeneration. In AD and cerebral amyloid angiopathy (CAA), aberrant complement activity contributes to amyloid-β (Aβ) accumulation, synapse loss, neuroinflammation, vascular dysfunction and blood-brain barrier (BBB) disruption. This review traces the evolving understanding of complement dysregulation in AD, from foundational findings in human studies to mechanistic discoveries in animal models and emerging induced pluripotent stem cell (iPSC)-derived cellular systems. It describes how genetic and epigenetic factors, including risk variants, chromatin modifications, and microRNAs, modulate complement pathways in the AD brain. Systemic influences, such as gut-brain axis disruption, are also considered for their potential to exacerbate complement-mediated neuroinflammation in AD. We further highlight efforts to identify biomarkers of complement activation and review the therapeutic potential of targeting complement components and regulators. By integrating molecular, experimental, and translational perspectives, this review outlines the multifaceted involvement of complement in AD and discusses key directions for future research and therapeutic interventions.

## Graphical Abstract

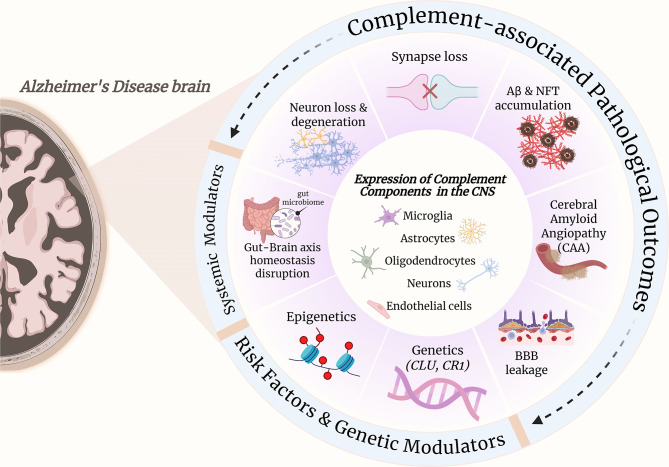

## Background

Neurodegeneration involves the progressive loss of neuronal structure and function and is a core feature of diverse neurological disorders including Alzheimer’s disease (AD), Parkinson’s disease (PD), Huntington’s disease (HD), and amyotrophic lateral sclerosis (ALS) [1]. Neurodegeneration is also linked to other neurological conditions such as ischemic stroke, traumatic brain injury (TBI) and vascular diseases. These conditions, while distinct in their aetiologies, can all lead to neuronal damage, cerebrovascular changes, functional decline, and risk of developing dementia [2]. AD, the most prevalent type of dementia, is characterized by extracellular deposition of amyloid-β (Aβ), intracellular neurofibrillary tangles (NFTs) and progressive synapse loss. It is a complex disease with a pathological continuum of degenerative events starting decades before the onset of clinical symptoms [3]. Despite decades of research into key proteins like Aβ and tau and strategies to combat neuroinflammation, AD remains difficult to treat effectively due to its complex and heterogeneous biology. Importantly, diverse combinations of risk or resilience factors affect distinct biological domains implicated in the development and course of AD pathogenesis [4]. Indeed the aetiology of AD is shaped by interplay of genetic risk factors, ageing and other, modifiable, risk factors including cardiometabolic disorders, environmental factors and lifestyle [5]. It is becoming more apparent that inflammatory processes represent a key driver of neurodegenerative diseases rather than acting as a bystander, with confirmation from genetic studies indicating many risk genes to be associated with inflammation and the innate immune response [6] including complement regulators like CLU, which encodes for clusterin, a weak negative regulator of membrane attack complex (MAC) assembly, and CR1, encoding for complement receptor 1 [7–9].

The complement system, a crucial component of innate immunity, has emerged as a key player in both central nervous system (CNS) development and neurological disease [10]. Originally characterized for its role in host defense and inflammation [11], recent research has revealed increasingly complex functions of complement proteins in the brain, ranging from synaptic pruning during development to involvement in various neurodegenerative conditions [12, 13]. Complement has well-established physiological functions in the CNS, with C1q and downstream components guiding synaptic pruning during development, ensuring refinement of neural circuits [14, 15]. Complement components also exert direct neuroprotective effects; C1q enhances neuronal viability and neurite outgrowth, modulates gene and microRNA expression, regulates neurotrophin levels and protects neurons from Aβ toxicity [16]. Understanding these diverse roles has become crucial for developing new therapeutic strategies for neurological disorders [17].

The complement cascade operates through three main activation pathways: classical, alternative, and lectin which are described in more detail in the next section. This cascade system provides a carefully regulated mechanism for immune response and tissue homeostasis, but its dysregulation can contribute to pathological conditions. Beyond the classical, alternative and lectin pathways, recent work has identified non-canonical routes of complement activation. Notably, granzyme K (GZMK), a serine protease released by cytotoxic lymphocytes, can cleave C2 and C4 to generate active convertases, thereby initiating full complement activation [18]. The role of complement dysregulation in neurological disorders has been extensively documented [19–23].

In AD, complement components participate in multiple pathological processes, including Aβ opsonization, chronic inflammation, and aberrant synaptic pruning [24, 25], driving neurodegeneration and cognitive decline. The clinical implications of complement research extend to both diagnostic and therapeutic applications, with some biomarkers like CLU, Factor I and terminal complement complex (TCC) able to predict disease progression in AD [26]. Understanding the precise mechanisms of complement dysregulation in different neurological conditions will be crucial for developing effective therapeutic strategies. As research continues to uncover new aspects of complement function in the CNS, the potential for therapeutic intervention grows. Future directions include the development of more specific inhibitors, improved delivery systems for crossing the blood-brain barrier (BBB), and a better understanding of the temporal aspects of complement activation in different disease states [12, 19]. The continued investigation of the role of the complement system in neurological disorders promises to yield new therapeutic strategies for these challenging conditions.

In this review, we report on the multifaceted role of complement activation in the pathogenesis and progression of AD. We discuss its evolving role in amyloid and tau pathology, synaptic loss, and neuroinflammation, as well as its contribution to cerebral amyloid angiopathy (CAA). We also highlight the genetic and epigenetic mechanisms regulating complement activity in the AD brain and consider peripheral influences, including gut–brain interactions. Finally, we review ongoing efforts to identify biomarkers of complement activation and evaluate the therapeutic potential of targeting complement components and regulators in AD.

## The complement system

### Overview of the complement system

It all started in the late 19th century when Jules Bordet was working in Élie Metchnikoff’s lab and discovered that serum contained a heat-labile component (inactivated at 56 °C for 30 min) that could lyse bacteria in the presence of specific antibodies [27, 28]. To characterize this bactericidal component, Bordet adopted the word ‘alexin’, introduced by Buchner in 1891 [29], which comes from the ancient Greek word *αλέξειν*, meaning ‘to defend’ [28, 30, 31]. In 1899, Paul Ehrlich was the first to use the now widely-known term *complement* [32] to describe how these newly-found components ‘complement’ the action of antibodies to fight pathogens. Over a century later, the complement system has been established as an evolutionarily conserved and sophisticated system [33] that plays a key role in innate immunity [34–38].

The complement system is recognized as one of the major effector mechanisms of the innate immune system and is composed of a complex network of plasma and membrane-associated serum proteins, which can robustly induce inflammatory and cytolytic immune responses against infection and injury [39]. Pepy’s seminal experiments in the 1970s revealed for the first time that complement also affects adaptive immunity [40, 41]. Researchers now define complement as a bridge between innate and adaptive immunity via regulating B- and T-cell responses, shaping the host’s ability to combat invasion from pathogens [39, 42]. More specifically, complement components such as C3a and C5a anaphylatoxins may interact with complement receptors on B- and T-lymphocytes, influencing processes like clonal expansion, memory cell formation, and antigen presentation [43, 44].

**Fig. 1:**
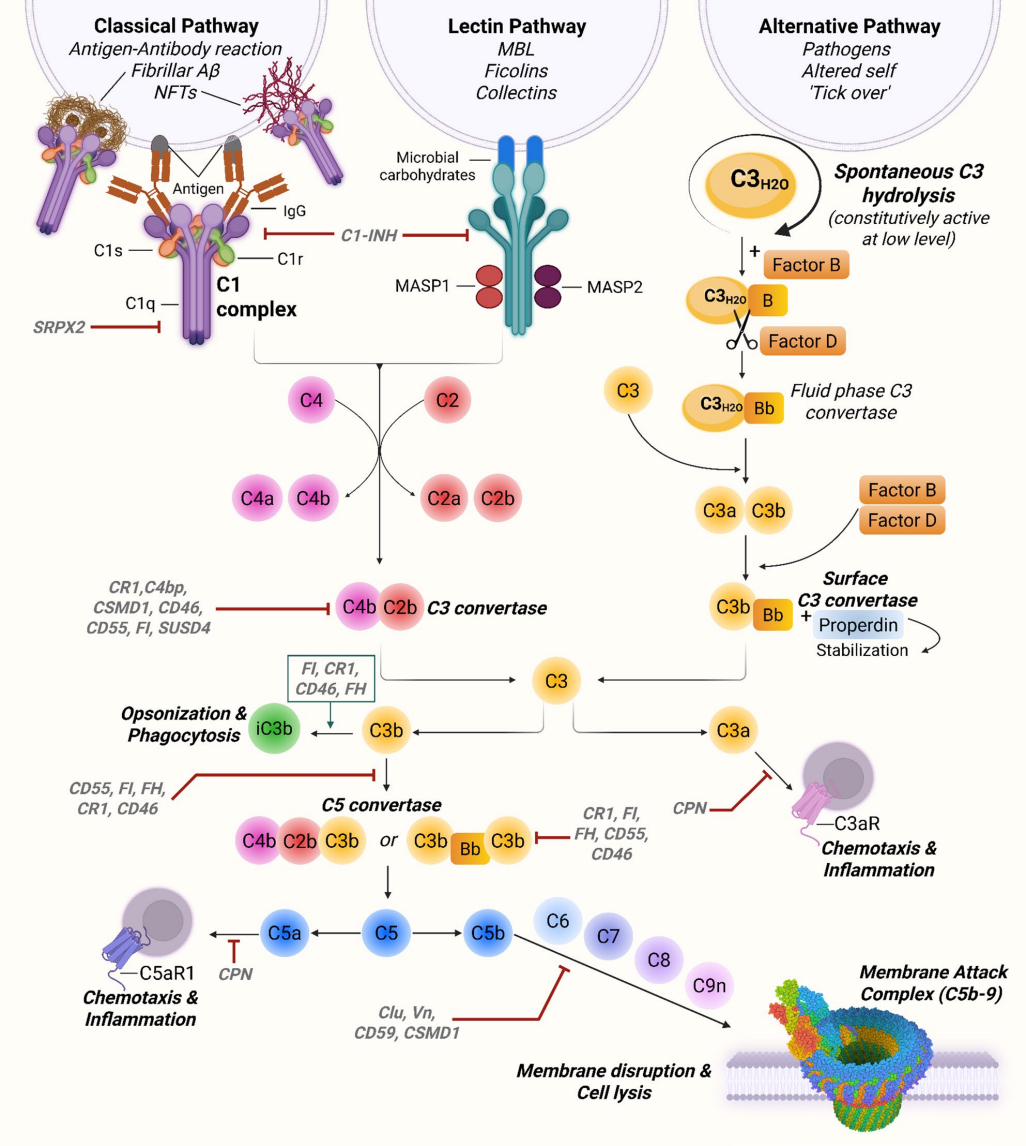
Overview of the complement cascade. (**A**) The complement system can be activated by three different pathways: the classical, lectin and alternative pathways. The classical pathway is initiated when complement component 1 (C1) complex binds to an activator surface, such as antibody complexes, fibrillar amyloid-β (Aβ), and neurofibrillary tangles (NFTs), and can be inhibited by SRPX2 (Sushi Repeat Containing Protein X-linked 2) via binding to C1q. The lectin pathway is activated when microbial carbohydrates bind mannan binding lectin (MBL) in complex with MASP1/2. C1-INH (C1 inhibitor) is the central regulator of the classical and lectin pathways, acting by inactivating C1r, C1s, MASP-1, and MASP-2. The alternative pathway is activated by spontaneous hydrolysis of C3 and formation of a C3 convertase. All three pathways converge at C3, which is cleaved by the C3 convertases into C3a and C3b. At the level of the classical/lectin C3 convertase (C4bC2b; historically known as C4bC2a), regulators including CR1 (Complement Receptor 1), C4bp (C4 binding protein), CSMD1 (CUB and Sushi Multiple Domains 1), CD46 (membrane cofactor protein), CD55 (decay-accelerating factor), FI (Factor I) and SUSD4 (Sushi Domain Containing 4) either accelerate convertase decay or act as cofactors for FI-mediated cleavage of C3b and/or C4b. C3a promotes chemotaxis and cell activation via C3a receptor (C3aR). C3b can be converted to iC3b, which opsonizes targets for microglial phagocytosis via complement receptor 3 (CR3). Conversion of C3b to iC3b requires FI together with cofactors including CR1, CD46, and FH (Factor H). C3b can also bind to C4bC2b or C3bBb to form the C5 convertases. C5 is then cleaved into C5a, which promotes chemotaxis and cell activation via C5aR1, and C5b, which binds to C6, C7, C8 and C9 to form the membrane attack complex (MAC; C5b–9), ultimately resulting in membrane disruption and cell lysis. As illustrated, C5 convertases are negatively regulated by CR1, CD55, and FH through decay-acceleration, as well as by CR1, CD46, and FH acting as cofactors for FI-mediated inactivation of C3b. C5a, as well as C3a, can be inactivated by CPN (carboxypeptidase N), which removes their C-terminal arginine, thereby reducing their ability to bind and signal through their respective receptors. Finally, terminal pathway regulators including clusterin (Clu), vitronectin (Vn), CD59 (protectin), and CSMD1, prevent MAC formation. MAC structure adapted from PDB ID: 6H04. Figure created with Biorender.com

Delving deeper, the complement cascade includes more than 50 soluble and membrane-bound proteins, regulators and receptors [45, 46]. Complement proteins comprise ~ 5% of total plasma protein [39], and are constitutively present in circulation in their inactive, soluble forms under homeostatic conditions [19]. Upon activation of the complement cascade, these proteins are proteolytically cleaved into fragments (such as iC3b and C3d), which are considered activation products and are not constitutively found in the blood [19]. Complement can be activated via three different pathways: the classical, lectin, and alternative complement pathways (Fig. 1). All three pathways converge on the cleavage of the C3 component, triggering downstream events leading to inflammation, opsonization, and target cell lysis. The classical pathway, the first to be discovered, is initiated when the C1 complex, composed of C1q and the serine proteases C1r and C1s, binds to an activator surface, such as antigen-antibody complexes, fibrillar Aβ, neurofibrillary tangles, or neuronal blebs [19]. This binding induces the cleavage of C4 and C2, resulting in the formation of the C3 convertase C4bC2b (historically known as C4bC2a) [47]. The lectin pathway is initiated by binding carbohydrates such as mannose-binding lectin (MBL), ficolins, and collectins on the surface of pathogens, activating two proteases, MASP1 and MASP2 (mannose-binding serine proteases 1 and 2), causing cleavage of C4 and then C2, whose fragments combine to form the same C3 convertase as in the classical pathway [48]. The alternative pathway is constitutively active at low levels in the blood via spontaneous C3 hydrolysis [49]. Upon pathogen recognition -in the case of e.g. viruses, fungi, bacteria, lipopolysaccharides (LPS), etc.- C3b binds Factor B, which is cleaved by Factor D, forming the alternative C3 convertase (C3bBb) [49]. Both C3 convertases cleave C3 into C3a and C3b. C3b can combine with the two different C3 convertases to form C5 convertase (C4bC2bC3b in the classical and lectin pathways and C3bBbC3b in the alternative pathway), which in turn cleaves C5 into C5a and C5b. Finally, C5b recruits C6, C7, C8, and multiple copies of C9 to form the membrane attack complex (MAC or C5b-9), which perforates target cell membranes [50]. Notably, a putative fourth pathway of complement activation has recently been described [18]. In this pathway, lymphocyte-derived Granzyme K acts as the initiator protease by directly cleaving C4 and C2, thereby propagating the entire cascade [18].

As mentioned above, complement activation exerts a range of effector functions. C3b and its inactivated Factor I-mediated cleavage product, iC3b, act as opsonins, tagging pathogens, apoptotic cells, or debris for clearance by phagocytes bearing complement receptors [44]. Specifically, C3b binds to complement receptor 1 (CR1) (Fig. 1), whereas iC3b engages complement receptor 3 (CR3) [44]. C3a and C5a are anaphylatoxins that bind their respective receptors, C3aR and C5aR, promoting chemotaxis, cytokine release, and immune cell activation [44]. Importantly, there are two forms of C5aR. C5aR1 (also known as CD88) is pro-inflammatory, while C5aR2 (formerly known as C5L2 or GPR77) may have regulatory or anti-inflammatory roles [51]. Beyond these core functions, complement also interacts with Toll-like receptor (TLR) signaling [52], Nod-like receptor family pyrin domain containing 3 (NLRP3) inflammasome activation [46], and coagulation pathways [53], amplifying inflammatory cascades and bridging innate and adaptive responses. The role of these pathways in the CNS is discussed later.

The tight regulation of complement activation is crucial for maintaining immune homeostasis, which is why the complement system is often described as “a double-edged sword”. Excessive or misdirected activation can result in severe host tissue damage. A complex network of fluid phase and membrane-bound regulatory proteins, including Factor H, CD55 (decay-accelerating factor), and CD59 (protectin) (Fig. 1), ensures precise control of complement attack, and adjusts its propagation and endpoints to the cellular target [44, 54]. More specifically, these regulators enable discrimination between self and non-self surfaces, thereby preventing the formation or stabilization of C3 and C5 convertases and inhibiting MAC assembly on host cells [54] (Fig. 1). Nevertheless, genetic variants, autoantibodies, or insufficient expression of complement regulators may cause dysregulation of the complement cascade, resulting in chronic inflammation, autoimmune disease, and tissue injury [54, 55]. These mechanisms play a central role in the pathogenesis of disorders such as systemic lupus erythematosus, age-related macular degeneration, cancer, and a range of neurodegenerative diseases, including AD, in which sustained or mislocalized complement activation exacerbates tissue damage and disease progression [56].

It should be emphasized that, over the past decade, there has been a fundamental change in our understanding of complement biology with the discovery of its crucial role in the intracellular space of a broad range of cellular populations and tissues [57, 58]. This intracellularly active complement system was defined as ‘the complosome’ [58]. From an evolutionary standpoint, the discovery of intracellular complement activity has formed the hypothesis that, in its most ancestral form, C3 may have been retained within the cell as a host defense protein, functioning to tag and neutralize microorganisms engulfed by primitive unicellular eukaryotes such as choanoflagellates [11, 45]. The role of the complosome has been shown to extend beyond traditional immune functions, serving as a central regulator of immune cell metabolism [59–61], autophagy [62–65], and gene transcription [66–70]. Its function is not limited within immune cells [57, 67, 68, 70–76], but expands across a variety of non-immune cell types and tissues [65, 77–84], orchestrating cellular homeostasis, modulating responses to both infectious and non-infectious stimuli, and controlling cell turnover and survival [46]. Altered complosome function has been implicated in the pathogenesis of various diseases, ranging from infection-related complications [66, 72, 85] to chronic inflammatory conditions [86, 87], cancer [88–90], and renal dysfunction [91, 92]. Although its role in the brain is only beginning to be understood, growing evidence suggests that its dysregulation may also contribute to neurodegenerative processes [93]. Thus, selectively modulating the complosome, potentially in combination with extracellular complement inhibition, could represent a promising therapeutic strategy for complement-mediated disorders, including those affecting the nervous system.

### Complement in the CNS

The hepatic origin of most complement components contributed to the long-standing assumption that the complement system operated exclusively in systemic immune responses. Over the years, it became progressively evident that complement proteins, as well as their receptors and regulators, are expressed throughout the CNS [94]. Currently, it is well established that CNS-resident cells, including neurons, astrocytes, microglia, and oligodendrocytes express complement proteins [95–97]. Notably, astrocytes are the primary source of C3 in the brain, while microglia are the dominant producers of C1q, particularly under inflammatory or neurodegenerative conditions [98]. Furthermore, astrocytes together with oligodendrocytes secrete C4, and neurons express C1q and C3, indicative of cell-intrinsic functions in synaptic regulation, beyond their classical immune roles [96]. Additionally, under stress or neurodegenerative conditions, astrocytes and mast cells have been shown to release C3a via connexin- and pannexin-based hemichannels, representing a non-canonical route of complement-mediated neuroinflammatory signaling [99].

The pivotal work of Stevens and colleagues [14] elucidated the fundamental role of the complement system in synaptic refinement during brain development. Following birth, excessive or weak synaptic terminals are eliminated by a process defined in 1982 as synaptic pruning [100], thereby effectively refining neural circuits. Early components of the classical complement pathway, such as C1q and C3, localize to synapses tagging them for removal by microglia expressing complement receptors, e.g. CR3/CD11b [14, 15, 101, 102]. During both development and adulthood, microglia prune synapses in an activity-dependent manner via CR3 recognition of synapse-bound iC3b [101]. In addition to CR3, CR1 has also been shown to modulate microglial reactivity. In vitro, pharmacological blockade of CR1 suppressed Aβ-induced microglial activation in primary rodent microglia, highlighting a functional role for CR1 in glial complement signaling [103]. In parallel, astrocytes contribute to synaptic engulfment through the C1q binding protein MEGF10 (multiple EGF-like domains 10) [104], as well as via MERTK (MER proto-oncogene, tyrosine kinase) and apolipoprotein E (APOE)-dependent phagocytic pathways, both of which operate in a C1q-dependent manner [105–107]. Notably, recent findings by Brott et al. revealed that C4-derived fragments engage human Leukocyte immunoglobulin-like receptor type B2 (LilrB2)/murine Paired immunoglobulin receptor B (PirB) with high affinity, linking complement activation to synaptic pruning in the AD brain [108].

Although C1q plays a well-established central role in synaptic pruning during both development and later in life, recent findings have revealed that it is dispensable for proper synaptic circuit refinement in the binocular zone of the primary visual cortex (V1b), pointing to region-specific mechanisms underlying synaptic pruning [109]. To this end, evidence regarding neuronally expressed complement regulators, including transmembrane proteins such as CSMD1 (CUB and Sushi Multiple Domains 1) (Fig. 1) [110–112], CSMD3 (CUB and Sushi Multiple Domains 3) [113, 114], and SUSD4 (Sushi Domain Containing 4) (Fig. 1) [115, 116], and the secreted protein SRPX2 (Sushi Repeat Containing Protein X-linked 2) (Fig. 1) [117, 118], has demonstrated their region-specific involvement in synaptic pruning [19, 119]. Importantly, aberrant synaptic pruning can lead to inappropriate synaptic retention or elimination, ultimately disrupting brain connectivity. Altered connectivity driven by complement dysregulation has been linked to neurodevelopmental and neurological disorders, including autism spectrum disorder [120], schizophrenia [121, 122], multiple sclerosis [123–125], TBI [126], stroke [127], and AD [128].

Taking a more detailed look at the implications of complement factors in the CNS, alterations in the expression or genetic regulation of C1q, C3, and C4 have been linked to aberrant synaptic pruning and dysfunctional connectivity in autism spectrum disorder and schizophrenia [120–122, 129, 130]. Complement components C3 and C3aR have been identified as key regulators of adult hippocampal neurogenesis [131, 132]. In multiple sclerosis, complement contributes to demyelination and lesion formation, with C1q, C3, and MAC deposition detected in both active and chronic plaques [22, 133, 134]. Following TBI or ischemic stroke, complement is rapidly activated at the site of injury, amplifying tissue damage through anaphylatoxin signaling and MAC-induced cell lysis [126, 135–137]. Over the past decades, data from human studies, in vivo and in vitro models, and omics approaches have elucidated the central and multifaceted role of complement in AD initiation, progression and exacerbation. This role spans from synaptic loss and glial activation to integration with genetic and epigenetic risk factors. Emerging evidence also implicates complement in anti-amyloid antibody-induced Amyloid-related Imaging Abnormalities (ARIA), the primary side effect of anti-amyloid immunotherapy in AD, in both nonclinical and clinical studies [138, 139]. Importantly, a recent retrospective case-control study of aducanumab-treated participants identified complement activation in regions corresponding to ARIA on magnetic resonance imaging (MRI) [140].

In light of these findings, the bidirectional crosstalk between peripheral and CNS complement players requires further investigation to better understand the progression of neuroinflammatory diseases. The BBB is a highly selective structure composed of tightly joined endothelial cells, vascular pericytes, and perivascular glia [141, 142]. Under physiological conditions, the BBB compartmentalizes the peripheral and central immune environments, effectively restricting circulating complement proteins from entering the brain parenchyma and enabling the CNS to regulate its own complement activity via local synthesis [13]. Nevertheless, BBB breakdown can occur with aging and sustained chronic inflammation [143], and is a hallmark of several neurological disorders, including multiple sclerosis, TBI, stroke, and AD [144–148]. This breach allows the infiltration of the brain by peripheral complement components, including C3, C5, and anaphylatoxins, amplifying neuroinflammation via the introduction of pre-activated or excessive complement proteins into an already vulnerable microenvironment [149, 150]. At the same time, brain-derived Damage-Associated Molecular Patterns (DAMPs), cytokines, or complement fragments may leak into the circulation and modulate systemic complement activation, creating a feedback loop that exacerbates immune responses on both sides of the barrier [151, 152]. Importantly, the choroid plexus has also been shown to undergo early complement-related proteomic changes in AD [153]. In the APP^NL−G−F^ mouse model, Delvenne and colleagues identified dysregulation of complement and immune signaling pathways in the choroid plexus as early as 7 weeks of age, with corresponding alterations in human cerebrospinal fluid (CSF) proteomes [153]. These findings reinforce the notion that peripheral-central immune crosstalk at barrier sites may drive early complement activation in AD.

This crosstalk has significant implications for therapeutic strategies: complement inhibitors targeting systemic components may have limited CNS efficacy when the BBB remains intact, whereas CNS-penetrant agents or dual-compartment approaches may be required in pathologies with documented barrier disruption [13]. Thus, it is of great significance to understand how complement flows and signals across this compartmental boundary in order to develop targeted and effective neuroimmune therapies. This is particularly relevant in AD, where both peripheral and central complement dysregulation converge, and where BBB dysfunction may further amplify complement-driven neurodegeneration. These insights provide a foundation for exploring the evolving understanding of complement in the context of AD.

## The evolution of complement in AD

### Evidence in human AD

Insights into complement involvement in AD began in the 1980s, when Eikelenboom and Stam reported the presence of complement proteins within amyloid plaques in late-stage AD postmortem brain tissue for the first time [154]. In the following years, these findings were further confirmed [155–159], sparking scientific interest towards the role of complement in AD pathophysiology. These studies revealed that early components of the classical pathway, such as C1q, C3, and C4, were particularly abundant in amyloid plaques and NFTs, whereas detection of terminal complement proteins was initially inconsistent [158, 160, 161]. By the late 1990s, however, the presence of MAC depositions was confirmed in neurons and plaques [162, 163], and complement component genes were shown to be upregulated in the AD brain [164]. The fact that complement activation progresses to the MAC stage indicates that the regulatory mechanisms of the complement system have been unable to halt the complement activation process [165]. Altogether, these findings led to the hypothesis that complement activation is not merely a consequence of neurodegeneration, but may actively contribute to disease progression. Nonetheless, several caveats should be noted when interpreting these early immunohistochemical studies. Although some antibodies, such as commonly used C1q reagents, have been validated in knockout mouse tissue, such validation cannot be performed in humans as complement knockout samples are not available. Moreover, the sticky nature of amyloid plaques and NFTs can lead to non-specific binding of both primary and secondary antibodies, raising the possibility of false-positive signals. Importantly, independent confirmation of these findings comes from proteomic and transcriptomic studies, which consistently identify complement changes in the human AD brain.

With the turn of the millennium, a new wave of studies sought to unravel the ‘mystery’ of complement and neuroinflammation in AD [165–167]. For instance, the use of Down’s syndrome brains as a temporal model for AD revealed that the classical complement activation follows the compaction of Aβ42 deposits and it may culminate in neuronal MAC expression in response to Aβ plaque maturation [168]. Seminal studies demonstrated that complement component C1q binds fibrillar Aβ, triggering activation of the C1 complex and inhibiting Aβ uptake by microglia [169–171]. Similarly, tau, as another antibody-independent activator of C1q, was shown to activate complement through C1q binding [172]. Notably, in patients with CAA, cerebrovascular Aβ colocalized with C3d and C9 [173]. Rogers and colleagues showed that in the periphery, Aβ is cleared by C3b-dependent adherence to CR1 on erythrocytes [174], highlighting the involvement of complement in Aβ trafficking and clearance beyond the CNS.

These initial findings laid the foundation for a series of studies over the past two decades exploring the involvement of complement in human AD. Studies examining complement proteins in CSF have reported increased levels of C3 and complement regulators such as Factor H and CR1 in patients with AD compared to controls [175, 176]. In line with these fluid biomarker findings, CR1 expression is now well documented in human brain tissue and induced pluripotent stem cell (iPSC)-derived microglia [177], and recent work has demonstrated a functional role for CR1 in mediating phagocytosis [178]. Furthermore, Wu et al. demonstrated increased levels of C3 in postmortem AD brains, especially at synapses, as well as in CSF, where it correlated with tau pathology [179]. In the aging human brain, C1q levels rise substantially and are predominantly localized near hippocampal synapses, suggesting a potential role in age-related synaptic vulnerability [180]. Similarly, C1q has been found to increase and associate with synapses in human AD brains [181]. Additionally, C1q-ApoE complexes were found in plaques and the choroid plexus, suggesting a role for APOE genotype in modulating C1q function at key neuroimmune interfaces [182, 183]. Chatterjee and colleagues reported elevated C1q in extracellular vesicles derived from the CSF of AD patients, further supporting its potential as a biomarker for AD [184]. These data reinforce the clinical relevance of complement activation in AD and suggest its potential utility as a biomarker of disease progression and synaptic dysfunction.

### Complement dysregulation in CAA in AD

CAA is characterized by the deposition of Aβ protein in the vascular walls of the meninges and vessels, predominantly affecting the cortical and meningeal vessels, with involvement of cortical structures like white matter, thalamus, basal ganglia and brainstem [185]. CAA is commonly linked to AD and Lewy body dementia, and, to a lesser extent, to frontotemporal lobar degeneration, corticobasal degeneration and supranuclear palsy [186]. Notably, approximately 90% of AD cases exhibit comorbid CAA pathology [187] with 25% having severe vascular amyloidosis [188], indicating overlap between both pathologies [189]. While extracellular Aβ deposition promotes tau pathology, CAA vasculopathy is associated with vascular inflammation, bleeds and stroke risk [190]. The deposition of complement fragments in vascular amyloid deposits has been reported [191–193]. These vascular amyloid deposits have also been associated with accumulation of many inflammatory mediators [194], including CLU (clusterin; Apo J), a potent complement regulator found at significantly higher levels in CAA patients than in those with AD or healthy controls, based on laser-captured cortical tissue proteome analysis [194].

Recent proteomic studies identified multiple proteins involved in CAA pathology. Proteome data from the leptomeningeal and large cortical vessels isolated from cryopreserved postmortem brain specimens of CAA cases identified an enrichment for apoE and CLU in the vessels of CAA patients compared to non-CAA patients. Also, in silico analysis identified enrichment for classical complement and extracellular matrix remodelling pathways in the differentially expressed vascular proteome [195, 196]. Furthermore, a recent large scale proteomics study on capillary microvessels in patients with confirmed capillary CAA, AD and healthy controls showed distinct proteomic profile in CAA cases independent of AD, with microvascular proteome from CAA brain showing enrichment for several proteins as reported in above studies including accumulation of complement C1qb, C1qc, C3 and complement regulator CLU, indicating the role of complement cascade in CAA-associated pathology [197]. This could overall influence Aβ aggregation or CAA clearance through a peri-arterial drainage process. In another proteomic study, comparison between CAA(+) vessels with neighbouring CAA(-) vessels in patients suffering from mild cognitive impairment (MCI) and AD showed proteins that are associated with vascular matrix reorganization and BBB breakdown [198].

CAA proteomics meta-analysis from some of the above studies indicated vascular upregulation of proteins like C3 and CLU in CAA cases with plaque amyloid pathology compared to vascular proteome of plaque-positive AD patients without any vascular amyloid [199]. Additionally, immunohistochemical studies of AD brains revealed expression of complement proteins and activated complement protein fragments C1q, C3c, C4d, C5b-9 in plaques [154] as well as in CAA vessels [191–193]. Altogether, these discoveries redefined the role of complement in AD and CAA expanding its function from a participant in inflammation to a critical regulator of synaptic and vascular health.

### Complement modulation in AD mouse models

Mouse models have emerged as a valuable tool in understanding the molecular mechanisms underlying the role of the complement cascade in AD. Thus, numerous investigations have leveraged AD mouse models to dissect the complex, context-dependent functions of complement. As mentioned previously, the discovery of the critical role of complement in mediating synaptic pruning during development [14], utilizing mouse models, constituted a cornerstone in our understanding of complement’s function in the pathogenesis of AD. This led scientists to hypothesise that this physiological mechanism could be aberrantly reactivated in neurodegenerative diseases, including AD. The importance of this hypothesis is reflected in the fact that there are no currently approved treatments for synaptic loss or neuronal death and dysfunction, which is the cause of cognitive loss in AD and the most impactful clinical presentation of this disease.

Evidence from mouse models has strongly supported this hypothesis. Hong et al. observed elevated C1q levels in the brains of J20 AD model mice as early as one month of age [200], mirroring findings of C1q mRNA upregulation at two months in the 3xTg AD model [201]. Notably, this increase occurred prior to detectable amyloid plaque deposition [200]. Early synapse loss, preceding plaque formation, was documented in both J20 and APP/PS1 mice at 3–4 months of age, particularly within the CA1, CA3, and dentate gyrus regions [200]. This loss was associated with increased C1q and C3 deposition at synapses and was absent in APP/PS1 mice genetically deficient for C3. In the same study, injection of oligomeric Aβ into wild-type mice resulted in synapse loss and enhanced microglial engulfment of synaptic material within 72 h [200]. These effects were not observed in C1q-deficient mice, in mice co-injected with anti-C1q antibodies, or in those lacking the C3b/iC3b receptor CR3 [200]. Together, these findings established a direct mechanistic link between complement tagging and early synapse loss in AD. They also identified CR3, the microglial receptor for iC3b and to a lesser degree C3b, as a key effector of synaptic phagocytosis in AD.

Others have explored the role of C1q in Alzheimer’s pathology using genetic deletion strategies. A foundational study by Fonseca et al. in APP transgenic mice, revealed that C1q deficiency significantly reduced microgliosis and neuritic damage, suggesting a detrimental role of the classical complement pathway in AD progression [202]. In Tg2576 mice, C1q deficiency reduced C4 deposition but unexpectedly increased astrocytic C3 expression and overall C3 deposition, likely via alternative pathway activation [203]. C1q-deficient mice showed reduced gliosis and neuronal loss despite elevated C3, suggesting that alternative pathway activation alone is insufficient to reproduce the detrimental effects associated with classical pathway activity [203]. In line with these findings, genetic deletion of C1q or C4 in PS2APP mice rescued synapse loss without altering cortical amyloid plaque burden, further implicating classical pathway activation as a direct driver of synaptic vulnerability [204]. In Arctic APP transgenic mice with microglia-specific C1qa deletion (via *Cx3cr1*-CreERT2), microglia were shown to be the primary source of brain C1q, while deletion reduced C1q levels and altered downstream complement activation [205]. In a tauopathy context, global C1q knockout in TauP301S (PS19) mice rescued synapse density by preventing both microglial and astrocytic engulfment of synapses [107]. More recently, Petrisko and colleagues reported that global C1q deficiency in Arctic and Tg2576 models altered gut microbiota composition, suggesting a potential role for C1q in mediating peripheral-central interactions during neurodegeneration [206]. In addition to genetic deletion, pharmacological inhibition of C1q has also been investigated. Administration of a C1q-blocking antibody in TauP301S tauopathy mice preserved synaptic density by preventing microglial-mediated synapse engulfment [181]. Furthermore, Wang et al. showed that antisense oligonucleotide (ASO)-mediated knockdown of C1r, C1s, or C4 protected synapses in PS2APP mice, demonstrating that targeted inhibition of the classical pathway can dose-dependently rescue synapse loss [204].

Beyond genetic and pharmacological targeting of C1q, recent studies have uncovered a diverse set of upstream and downstream mechanisms that govern its synaptic actions in AD mouse models. In APP/PS1 mice, inhibition of metabotropic glutamate receptor 1 (mGluR1) signaling mitigated microglial phagocytosis of the glutamatergic synapses and restored synaptic strength and cognition [207], while mGluR5 inhibition prevented C1q synaptic tagging and reversed synapse loss in both APP/PS1 and APP^NL−G−F^/hMAPT mice [208]. Other work has linked synaptic vulnerability to broader cellular and systemic changes. In APP/PS1 mice, synaptic mitochondrial dysfunction and septin accumulation promoted C1q-mediated pruning [209], while periodontal infection in APP^NL−G−F^ mice increased microglial C1q expression and exacerbated synapse loss [210].

Importantly, several endogenous regulators of C1q have also been identified. In aged TauP301S mice, neuronal pentraxin 2 (Nptx2) overexpression ameliorated synapse loss, potentially via C1q inhibition [211]. In 5xFAD mice, triggering receptor expressed on myeloid cells 2 (TREM2) was shown to bind C1q and prevent excessive pruning, suggesting a microglial-mediated protective role of TREM2-C1q complex formation in the pathogenesis of AD [212]. Recent transcriptomic and proteomic analyses in APP^NL−G−F^ mice further revealed that TREM2 deficiency reduces microglial expression of *C1qa*, *C1qb*, and *C1qc*, disrupts plaque engagement, and exacerbates dystrophic neurite formation, reinforcing TREM2’s regulatory role in complement-mediated synaptic vulnerability [213]. ApoE was also identified as a key endogenous regulator of classical complement activation through its ability to bind C1q and return complement signaling to homeostasis [182]. This interaction was reduced in mice expressing the human APOE ε4 allele, resulting in sustained complement activity [182]. Tyrosine kinase binding protein (TYROBP) deficiency markedly reduced C1q expression in both APP/PS1 and TauP301S mice, indicating shared upstream regulation across AD models [214, 215]. Moreover, in the FAD^4T^ model, microglial upregulation of C1qA was associated with synapse loss and cognitive decline [216]. Finally, in 5xFAD mice, microglial CD2-associated protein (CD2AP) deficiency reduced C1q-mediated pruning [217], while physical exercise inhibited C1q-mediated microglial engulfment through downregulation of transmembrane protein 9 (Tmem9), ultimately improving cognitive function [218].

Extending beyond C1q-driven synaptic tagging, C3 and its downstream signaling components have emerged as central effectors of complement-mediated dysfunction in AD mouse models. Shi and colleagues reported a region- and age-dependent decline in hippocampal synaptic puncta density, dendritic spine density, neurons, long-term potentiation (LTP), and cognitive performance in wild-type mice, deficits that were markedly attenuated or absent in age-matched C3 knockout mice [219]. This was further supported by a second study by Shi et al., who demonstrated that C3 deficiency in APP/PS1 mice not only preserved synaptic integrity and cognitive performance in aged mice but also attenuated microgliosis, astrogliosis, and proinflammatory cytokine expression, despite ongoing amyloid pathology [220]. Wu et al. showed that C3 deletion protected against neurodegeneration in both PS2APP and TauP301S mouse models, establishing C3 as a central pathological driver [179]. In addition, Carpanini and colleagues reported elevated levels of C3b/iC3b activation fragments in the brains of aged APP^NL−G−F^ and 3xTg mice [221]. Furthermore, C3aR deletion in TauP301S mice attenuated tau pathology and normalized immune gene networks [222], while C3aR knockout in APP^NL−G−F^ mice enhanced microglial Aβ clearance and improved cognition [223]. Importantly, the findings in TauP301S mice were not replicated in a more recent study, which reported that C3aR deletion did not attenuate neurodegeneration or alter acute inflammation-induced gene expression changes in the brain [224]. Beyond neuronal pathology, endothelial C3aR has been shown to mediate vascular inflammation and BBB permeability during aging [225], as well as hippocampal pathology and cognitive impairment in VCID (vascular contributions to cognitive impairment and dementia) mouse models [226]. Pharmacological C3aR inhibition reduced tau pathology and improved cognition in TauP301S mice [227]. Nonetheless, significant caveats accompany these findings as the compound SB290157 has consistently been shown to display partial agonist activity, off-target effects, and poor pharmacokinetic properties, which limits its suitability as a tool for probing C3aR inhibition in vivo [228, 229]. Notably, overexpression of a soluble variant of the rodent-specific complement regulator *Crry* (complement receptor 1-related gene/protein y), which inhibits C3 activation by acting as a Factor I cofactor and by accelerating the decay of classical and alternative pathway C3 convertases, reduced hyperphosphorylated tau levels in aged P301L/sCrry double-transgenic mice [230]. However, it was also reported that local silencing of membrane-bound Crry by lentiviral injection in the cortex and hippocampi of TauP301S mice reduced neuroinflammatory cytokines and complement C3 components, leading to improved neuronal integrity and attenuated disease progression [231]. Taken together, these studies underscore that C3 and its downstream signaling components orchestrate key pathological processes beyond initiation, contributing to both neurodegeneration and impaired vascular integrity in AD.

Several recent studies have highlighted “complementary” mechanisms that modulate complement activity and microglial responses in AD rodent models. For example, Factor H, known as a key regulator of the alternative pathway, has emerged as a promising therapeutic target, with evidence suggesting that enhancing its function may restrain complement overactivation in APP/PS1 mice [232]. At the level of microglial checkpoints, loss of SIRPα (Signal Regulatory Protein alpha), a ‘don’t eat me’ signal, led to exaggerated synaptic pruning in APP/PS1 mice [233]. Vascular dysfunction has also emerged as a contributor to complement-mediated pathology. Fibrinogen leakage into the brain parenchyma induces microglia-mediated dendritic spine elimination via CD11b (part of CR3), leading to synapse loss and cognitive impairment in 5XFAD mice [234]. Similarly, chronic cerebral hypoperfusion was shown to activate both coagulation and complement cascades in APP23 mice, linking vascular stress to innate immune activation [235]. Complement receptors have additionally been implicated in the clearance of pathological proteins: complement receptor 4 (CR4) was recently shown to mediate the uptake of extracellular tau fibrils by microglia, suggesting that complement receptor signaling may facilitate aggregate removal downstream of iC3b opsonization [236]. Such studies expand our understanding of how complement activity is fine-tuned in the AD brain, bridging upstream activation with cellular responses.

The C5a–C5aR1 signaling axis has emerged as a critical driver of neuroinflammation and synaptic dysfunction in AD mouse models. Pharmacological inhibition of C5aR1 using the antagonist PMX205 has consistently demonstrated therapeutic benefit in transgenic AD models such as Tg2576 and Arctic48, where it reduced amyloid burden, gliosis, and cognitive decline [111, 237]. Similarly, active immunization against C5a improved memory and reduced inflammatory markers in Tg2576 mice [238]. In contrast, a study using intermittent administration of EP67, a conformationally biased analog of C5a (C5aR1 agonist) engineered to preferentially activate mononuclear phagocytes over neutrophils [239], reported enhanced microglial phagocytosis of Aβ and preserved memory in 5xFAD mice [240]. However, EP67 is not a selective C5aR1 agonist, as it can also activate C3aR and C5aR2, the latter of which has been associated with anti-inflammatory signaling [241]. Therefore, the beneficial effects observed in [240] may have resulted from this broader receptor activity or from the specific intermittent dosing schedule, rather than direct, sustained C5aR1 activation. Together, these considerations highlight that the outcomes of complement receptor agonism depend not only on dose but also on ligand specificity and dosing context [239, 242]. Supporting a pathogenic role for sustained C5a–C5aR1 signaling, Carvalho and colleagues showed that C5a overexpression in Arctic mice accelerated cognitive decline and inflammatory gene expression, whereas C5aR1 deletion delayed these changes [243]. Additional studies demonstrated that C5aR1 antagonism promotes a neuroprotective microglial phenotype [244], dampens inflammatory glial signaling [111], and protects against region- and age-dependent synaptic loss in Tg2576 and Arctic48 models [245]. Notably, C5aR1 deletion has also been associated with age-dependent shifts in gut microbiota composition in both wild-type and AD mouse models, suggesting a potential role for peripheral immune signaling in broader neuroimmune dynamics [206]. Collectively, these findings establish C5aR1 as a critical effector of complement-mediated neurotoxicity and a promising target for disease modification in AD.

Mounting evidence implicates the terminal complement pathway, and in particular, MAC formation, as a critical effector of neurodegeneration in AD. Genetic deletion of CD59a, the endogenous inhibitor of MAC, in tauopathy models exacerbated tau pathology and neuronal loss, underscoring the detrimental effects of unrestrained MAC activation [230]. In 3xTg-AD mice, genetic deletion of C6 significantly reduced synaptic loss, supporting a pathogenic role for MAC formation in synapse degeneration [221]. In the APP^NL−G−F^ model, pharmacological inhibition of the terminal pathway using an anti-C7 antibody preserved dendritic spine density near amyloid plaques, further implicating MAC in amyloid-associated synaptic vulnerability [221]. More recent studies by Zelek et al. showed that both genetic deletion of C7 and systemic administration of an anti-C7 antibody in APP^NL−G−F^ mice led to reduced complement activation, decreased amyloid burden, preserved synaptic density, and improved cognitive function, demonstrating that targeting the terminal pathway can mitigate multiple AD-related pathologies [246]. Moreover, a brain-penetrant anti-C7 antibody was reported to cross the BBB, inhibit MAC formation, and protect against neurodegeneration in APP^NL−G−F^ mice [247]. Extending the relevance of MAC activation beyond parenchymal pathology, Hu et al. demonstrated that macrophage-derived migrasomes (large extracellular vesicles formed during cell migration) accumulate along cerebral vessels and induce complement-dependent cytotoxicity and BBB disruption in a model of CAA (Tg-SwDI/B mice), highlighting the role of terminal complement activation in cerebrovascular injury [248]. Together, the data support terminal complement inhibition as a promising strategy to counteract synaptic, cognitive, and vascular decline in AD.

Recent advances in spatial transcriptomics have provided a powerful new perspective on how complement activation unfolds in a neuroanatomical context. In APP^NL−G−F^ mice, Chen et al. used in situ sequencing to identify a plaque-induced gene (PIG) network enriched for complement components, including C1q and C4, that were specifically upregulated in microenvironments surrounding amyloid plaques [249]. This spatially restricted expression pattern was also observed in human AD brain tissue, reinforcing the concept that complement activation in AD is not diffuse but instead confined to local inflammatory niches [249]. These findings underscore the importance of spatial resolution in dissecting complement-mediated neuroinflammation and synaptic vulnerability.

While the studies discussed above emphasize the crucial roles of complement activation in AD, it is important to recognize that these effects can vary depending on the disease model. Fonseca and colleagues investigated the impact of C1q, C3, and C5 deficiency in 3xTg-AD mice and found that the contribution of individual complement components to amyloid pathology and neuroinflammation differed in other transgenic models (Tg2576 and Arc48) [250]. Similarly, Maier et al., found detrimental effects of germline C3 deficiency in J20 APP transgenic mice [251], whereas Shi et al. found neuroprotective effects of germline C3 deficiency in APP/PS1 mice [220]. These findings highlight that the consequences of complement modulation are not universally consistent and may be influenced by factors such as the underlying transgenic background, disease stage, and pathological burden. This variability reinforces the importance of context when interpreting nonclinical data and suggests that complement-targeted therapies should be tailored to model- and disease stage-specific dynamics. Nevertheless, a mega-analysis of transcriptomic data across 10 AD mouse studies identified *C1qa*, *C1qb*, and *C1qc* as among the most consistently upregulated genes during the early disease stages [252]. These transcriptional changes preceded major pathological shifts and were not solely attributable to gliosis, suggesting that classical complement activation may represent a common and early hallmark of AD pathogenesis across models [252]. Proteomic and lipidomic analyses in APP/PS1 mice further supported this, revealing early upregulation of complement and coagulation components, consistent with complement activation as an early molecular event in AD [253]. Furthermore, computational analyses have reinforced the cross-species relevance of complement involvement in AD. Lee et al. identified conserved molecular signatures between AD mouse models and human brain tissue, including complement cascade activation, TYROBP-associated innate immune responses, and Tyro3/Axl/MerTK (TAM) receptor agonists, distinct from changes attributable to aging [254]. Varma et al. found age- and sex-specific changes in CSF complement proteins (C1qa, C3, C9, Serping1, CFI) in Caribbean vervet non-human primates that develop cerebral amyloidosis with aging, noting elevations in males and reductions in females [255].

At the cellular level, complement-dependent signaling between microglia and astrocytes plays a pivotal role in shaping Aβ dynamics and driving AD progression. This bidirectional communication is increasingly recognized as a core component of AD-related neuroinflammation. The role of microglia as complement-dependent phagocytes in the diseased brain marked a major advance in our understanding of AD pathophysiology. Nonetheless, their role expands beyond phagocytosis. As AD pathology progresses, microglia produce pro-inflammatory cytokines, including interleukin 1 alpha (IL-1α), tumor necrosis factor alpha (TNF-α), and C1q, which act on nearby astrocytes to induce a neurotoxic phenotype [256]. These neurotoxic, reactive astrocytes are unable to promote neuroprotective functions, synaptogenesis, and phagocytosis, and lead to neuronal and oligodendrocyte death [256]. Moreover, neuronal Aβ activates nuclear factor-κΒ (NF-κB) signaling in astrocytes, leading to the extracellular release of complement C3a [257, 258]. Released C3a binds to C3aR expressed on microglia and neurons, amplifying Aβ pathology through a ‘feedforward’ loop. Pharmacological blockade of this signaling cascade using a C3aR antagonist attenuates microglial activation and reduces Aβ accumulation. These findings highlight the NF-κB/C3/C3aR axis as a key mediator of astrocyte-microglia crosstalk in the context of Aβ-driven pathology [257, 258]. Of particular note, C1q has also been suggested to act as an AD-specific modulator of the cellular crosstalk between microglia and astrocytes [259], as well as a potential regulator of astrocyte-mediated Aβ clearance [260]. Adding to this multicellular complement-signaling axis, recent work has shown that perivascular macrophages can induce a complement-expressing, phagocytic state in microglia via secretion of SPP1 (secreted phosphoprotein 1/osteopontin) [261]. In APP^NL−F^ mice, SPP1-driven upregulation of *C1qa* in microglia led to enhanced synaptic engulfment, while genetic ablation of *Spp1* prevented synapse loss despite ongoing amyloid deposition [261].

### Emerging cellular models of complement in AD

Translating insights from animal models to human disease remains a key challenge in AD and complement research. Human iPSC-derived models, particularly when paired with Clustered Regularly Interspaced Short Palindromic Repeats (CRISPR)-based gene editing and co-culture approaches, now provide a platform to investigate complement-driven processes such as synaptic pruning and neuroinflammation. Although still limited in maturity and complexity, these systems represent a promising tool to bridge complement findings from animal models to human disease.

Protocols for generating microglia-like cells from human iPSCs (iMGLs) now allow for functional studies of innate immune signaling in a human context [262]. These iMGLs express complement-related genes and have been used to investigate AD risk variants such as TREM2, which modulates microglial activation, and may shape complement responses [263]. TREM2 variants have been shown to alter the functional properties of iPSC-derived microglia, including their response to chemotactic cues such as complement component C5a [264]. Importantly, CR1 expression has been demonstrated in iPSC-derived microglia, providing a platform to connect human complement genetics with cellular mechanisms relevant to AD [177]. In parallel, iPSC-derived astrocytes have been used to model inflammatory activation and neurotoxicity, with reactive astrocytes serving as a major source of C3 and potentially contributing to synapse elimination [265]. Collectively, these studies highlight how stem cell–derived systems can be leveraged to probe complement mechanisms relevant to AD.

CRISPR-based strategies are emerging as powerful approaches to dissect complement mechanisms in human cellular models. For example, a CRISPR interference and activation (CRISPRi/a) platform in iPSC-derived microglia was used to identify regulators of inflammatory activation, survival, and phagocytosis, processes that intersect with complement signaling in neurodegeneration [266]. In parallel, CRISPR/Cas9 editing has been employed to generate human cell lines with targeted knockouts of complement regulatory proteins. Thielen and colleagues established human cell lines lacking CD46, CD55, and CD59 (Fig. 1), providing robust tools to study complement activation and regulation directly in a human context [267]. These innovative approaches highlight how gene editing can be applied to uncover complement-specific pathways and identify potential therapeutic targets in AD.

Beyond monocultures, co-culture and organoid systems offer more physiologically relevant platforms to study complement across interacting human brain cell types. For instance, cellular specificity within the complement system could be explored by selectively manipulating complement genes in defined cell types using CRISPR, and examining the resulting effects within co-culture systems. Notably, a tri-culture model comprising iPSC-derived neurons, astrocytes, and microglia demonstrated that microglia-astrocyte communication induces robust astrocytic C3 expression, especially in the presence of *APP* mutations, modeling complement-driven inflammatory signaling in an AD-relevant context [259]. In three-dimensional systems, brain organoids containing innately developed microglia responded more effectively to β-amyloid challenge than those with externally added microglia, suggesting that microglial ontogeny may influence complement-related responses [268]. In addition, long-term adhesion organoids with integrated iPSC-derived microglia showed reduced tau pathology, preserved synaptic density, and neuronal protection, underscoring the potential of these systems to recapitulate complement-associated neurodegeneration [269]. These models broaden the capacity of iPSC-based systems to investigate complement signaling within complex multicellular environments relevant to AD.

While iPSC-derived systems provide powerful tools to study complement in a human context, they face important limitations, including developmental immaturity, lack of vasculature and aging, and limited capacity to model chronic, progressive inflammation [270, 271]. Nevertheless, emerging approaches based on direct differentiation (transdifferentiation or direct reprogramming) are beginning to address the limitation of developmental immaturity observed in iPSC-derived systems [272, 273]. Differences in complement gene expression between iPSC-derived cells and adult human brain tissue further constrain translational interpretation, particularly in the context of aging and AD [274]. Even so, these systems remain uniquely genetically tractable and, when combined with genome-editing and co-culture approaches, are poised to yield critical insights into complement dysregulation and to accelerate therapeutic discovery in AD.

### Non-canonical roles of complement

Beyond its well-established extracellular functions, complement activity has also been detected inside cells [60, 275]. Complement proteins C3 and C5 have been found intracellularly where they regulate fundamental processes including metabolic reprogramming [57, 61, 67, 276], mitochondrial ROS production and stress signaling [275, 277], as well as autophagy regulation [278]. For example, intracellular C3 sustains the mechanistic target of rapamycin (mTOR)-dependent metabolic pathways in T cells [57], while C3 and C5 signaling within subcellular compartments cross-talks with innate sensors such as the NLRP3 inflammasome and the mitochondrial antiviral signaling protein (MAVS), linking complement activity to mitochondrial stress responses and inflammasome activation [275, 277]. Although the existence of a functional intracellular convertase remains unproven and is biochemically implausible under endosomal conditions, these findings suggest that complement proteins can exert non-canonical, cell-intrinsic roles independent of the traditional cascade [279].

Within the CNS, complement proteins have also been implicated in non-canonical intracellular and synaptic functions. In particular, C1q and C3 have been suggested to modulate synaptic communication and neurotransmission (reviewed in [280]). Additionally, microglia-derived C1q has been detected within neuronal ribosomes in the aging mouse brain, raising the possibility of intracellular complement activity relevant to neuronal homeostasis [281]. Together, these findings point to a growing body of evidence that complement functions extend beyond classical immunity to encompass synaptic modulation, proteostasis, and cellular stress responses.

Although direct evidence in AD models is limited, non-canonical complement functions could plausibly intersect with pathways central to neurodegeneration, including energy metabolism, mitochondrial dysfunction, inflammasome activation, and neuronal stress responses. As tools for high-resolution subcellular profiling and genetic manipulation advance, systematic investigation of these alternative roles may uncover novel mechanisms linking innate immunity to AD pathology.

Decades of research have reshaped our view of complement in AD from an amyloid-associated bystander to a central regulator of synapse loss, inflammation, and vascular dysfunction. Animal models and human-iPSC systems have uncovered diverse, context-dependent roles for key components such as C1q, C3, and C5aR1, while emerging work on intracellular complement points to additional, underexplored mechanisms. These findings establish complement as a dynamic contributor to AD pathogenesis. The next section explores how genetic and epigenetic factors shape this system and modulate disease risk.

## Genetic & epigenetic implications of complement in AD

### Genetic associations between complement and AD risk

For over 16 years (1993–2009), *APOE* remained the only major genetic risk factor identified in sporadic AD. Numerous studies consistently confirmed that the ɛ4 allele increases AD risk, while the ɛ2 allele appears to confer a protective effect [282, 283]. Early genome-wide association studies (GWAS) in AD, conducted between 2007 and 2009, were constrained by limited cohort sizes, often fewer than 1,000 individuals per group, which reduced their ability to detect risk variants for such a genetically complex disorder [284–289]. This changed in 2009, when two landmark GWAS studies identified novel susceptibility loci for late-onset AD (LOAD), for the first time since the discovery of APOE. Lambert and colleagues reported genome-wide significant associations for *CLU* and *CR1* [9], while Harold et al. independently confirmed the *CLU* signal and identified *PICALM* (phosphatidylinositol binding clathrin assembly protein) as an additional risk gene [7]. Unlike rare mutations in *APP*, *PSEN1*, or *PSEN2* that cause early-onset familial AD, the variants identified in these studies contribute to risk for the more common, polygenic form of late-onset, sporadic AD. The strength of GWAS lies in their ability to scan hundreds of thousands of variants in an unbiased, hypothesis-free manner. Therefore, the fact that both studies independently identified *CLU* in a genome-wide context, further reinforced its association with AD [290]. These findings demonstrated that sufficiently powered GWAS could uncover biologically meaningful variants, many of which implicate immune pathways, endocytic trafficking, and lipid metabolism in AD pathogenesis.

The *CLU* gene encodes clusterin which is also known as apolipoprotein J (ApoJ) [291], and has long been regarded as a risk gene in AD, leading to the accumulation of substantial functional evidence over the years (Table 1). For instance, in 2004, DeMattos and colleagues observed that ApoE and clusterin cooperatively regulate extracellular Aβ metabolism in vivo [292]. Genetic deletion of either protein led to increased Aβ accumulation, while combined deletion resulted in an even greater burden, supporting their complementary roles in Aβ clearance and deposition suppression [292]. It is now known that CLU, a ~ 75 kDa extracellular chaperone protein, regulates the complement system by inhibiting MAC formation and facilitating the clearance of cellular debris and misfolded proteins, including Aβ [293, 294]. It is highly expressed in astrocytes and upregulated in the AD brain, where it may exert both protective and potentially detrimental effects, depending on the disease state and molecular context [294–296]. CLU has been shown to reduce Aβ aggregation, gliosis, and neurotoxicity, and to support synaptic integrity by modulating astrocyte and microglial activity [295, 296]. Notably, recent work also demonstrated that clusterin, while protective against Aβ aggregation, can paradoxically enhance tau seeding and propagation in vivo [297]. Earlier studies further suggest that CLU may stabilize toxic Aβ oligomers, promote plaque formation, or exacerbate neuritic damage under certain pathological conditions [298, 299]. Although the precise mechanisms by which *CLU* variants influence AD risk remain unresolved, studies suggest that genetic variation near the *CLU* locus affects both its expression and alternative splicing, potentially modifying its neuroprotective functions and complement-modulating capacity [300, 301].

The *CR1* gene encodes a membrane-bound receptor that primarily binds C3b and C4b to mediate immune complex clearance [8], and has consistently emerged as a significant genetic risk factor in AD (Table 1). GWAS and meta-analyses have identified robust associations between LOAD and single nucleotide polymorphisms (SNPs) within *CR1*, particularly rs6656401 and rs3818361, across European and Chinese populations [9, 302–307]. In Han Chinese individuals, additional missense variants have been linked to increased AD risk, underscoring ethnic-specific genetic susceptibility [308]. Structural polymorphisms, including intragenic duplications that generate the *CR1-S* isoform, increase C3b/C4b binding sites but may impair Aβ clearance due to altered receptor trafficking and reduced peripheral clearance, especially via erythrocytes [8, 309–311]. Although minimally expressed under normal conditions, CR1 is upregulated in AD microglia and astrocytes and has been linked to greater amyloid burden, CAA, cognitive decline, and depression [177, 312–315]. Recent functional work further implicates CR1 in the regulation of microglial phagocytosis [178]. CR1 also appears to interact with APOE-ε4 to accelerate cognitive decline [316]. Notably, the rs3818361 CR1 variant has been shown to modulate the relationship between plasma ApoE levels and cortical amyloid deposition, indicating that *CR1* genotype may influence amyloid accumulation through ApoE-dependent mechanisms [317]. Functional assays have shown that AD-associated CR1 variants modestly increase C3b and C1q binding, suggesting altered complement clearance dynamics in risk carriers [315]. Altogether, these studies suggest that CR1 contributes to AD pathogenesis through combined effects on immune complex handling, amyloid processing, and neuroinflammatory responses.

While *CR1* and *CLU* represent the most robust and consistently replicated complement-related genetic risk loci in AD, several additional complement genes have been suggested by candidate gene studies, rare variant analyses, or pathway-level enrichment analyses. These include *C1S*, a serine protease of the classical pathway [204], and complement Factor H (*CFH*), a regulator of the alternative pathway more strongly associated with age-related macular degeneration but functionally relevant to neuroinflammation [318–321] (Table 1). Another interesting candidate is *CSMD1*, encoding a transmembrane protein that inhibits classical complement activation and is highly expressed in the brain, where it may regulate synaptic refinement and modulate neuroimmune responses [112, 322, 323]. Though associations involving these genes have not consistently reached genome-wide significance or have shown limited replication, they highlight the broader involvement of the complement cascade in AD susceptibility (Table 1).

In summary, common variants in complement genes, particularly *CR1* and *CLU*, act as risk modifiers in sporadic AD, showcasing complement’s relevance in the development of LOAD.

### Beyond the genome: epigenetic mechanisms governing complement in AD

Genetic variation alone cannot fully account for complement dysregulation in AD. Over the past 20 years, increasing evidence points to epigenetic regulation as a key mechanism shaping complement gene expression in the aging and diseased brain [324, 325]. Processes such as DNA methylation, histone modifications, chromatin remodeling, and non-coding RNA activity dynamically influence gene accessibility and transcription in response to intrinsic and extrinsic cues. These layers of regulation may unravel how complement activity varies by age, sex, brain region, or inflammatory state, and how it contributes to disease progression even in the absence of strong genetic drivers. This subsection examines the emerging evidence linking epigenetic mechanisms to complement pathway dysregulation in AD.

DNA methylation is a fundamental epigenetic mechanism that involves the addition of methyl groups to cytosine residues predominantly occurring in cytosine-phosphate‐guanine (CpG) dinucleotides [326]. It plays a critical role in regulating gene expression across development, aging, and disease. Over the past decade, major technological advances have enabled the first epigenome‐wide association studies (EWAS) of DNA methylation in AD brain tissue [327, 328]. Such studies have reported altered methylation at complement-related loci, suggesting that genes such as *CLU* and *CR1* may be epigenetically modulated in AD (Table 1). For instance, hypomethylation at CpG island shores (regions flanking CpG-rich promoter sequences that are particularly susceptible to age- and disease-related methylation changes) has been observed at *CLU*, *CR1* and *PICALM* in the blood of Japanese AD patients [329]. In the same study, *CLU* methylation levels, in combination with APOE genotype, could distinguish AD patients from cognitively healthy elderly individuals with high sensitivity and specificity [329]. Thus, methylation differences at the CpG island shores of AD-associated genes may have diagnostic value in AD, however, further studies are required to exclude ethnic-specificity. In postmortem AD brain tissue, DNA methylation at specific CpG sites within *CLU* and *CR1* has also been associated with neuritic plaque burden, independently of genetic variation [330]. Importantly, at the *CR1* locus, the direction of this association was modified by rs6656401 genotype, highlighting a gene-epigenome interaction that may influence complement-related pathology in AD [330].

Additional evidence suggests that *CLU* methylation may also relate to cognitive function independently of disease status. In a cohort of healthy and schizophrenia subjects, Alfimova et al. found that DNA methylation within a regulatory region of the *CLU* gene predicted performance on episodic verbal memory tasks, suggesting that epigenetic variation at this AD-associated locus may influence cognition beyond genetic background [331]. Consistent with this, a recent multi-omics Mendelian randomization study identified *CLU* as an AD-causal gene whose brain expression is influenced by DNA methylation in the context of mitochondrial dysfunction and inflammation, further supporting its epigenetic sensitivity in AD [332]. Supporting its epigenetic plasticity, the *CLU* promoter contains several binding sites for stress-responsive transcription factors, including AP-1 (activator protein 1) and HSF (heat-shock factor) [333], and lies within a CpG-rich domain susceptible to methylation [334–336]. Epigenetic regulation of *CLU* has also been observed in multiple cell types, including prostate cancer cells, neural cells, and retinal pigment epithelial cells [334–336], suggesting that *CLU* expression is epigenetically modulated by factors such as aging, stress, and inflammation, known contributors to AD pathology. Further supporting this, Gasparoni et al. performed a cell type-specific DNA methylation analysis in purified human neurons and glia, identifying differential *CLU* methylation in neurons, reinforcing the idea that complement gene regulation in AD may differ across cell types [337].

Methylation patterns may also differ significantly by sex. Recent analyses have revealed sex-specific methylation changes in AD brains, potentially affecting immune and complement-related genes [338]. In particular, Zhang and colleagues observed that in male samples, differentially methylated genes were significantly enriched for complement system-related processes, suggesting sex-dependent epigenetic regulation of complement activity [338]. Collectively, these findings support DNA methylation as a mechanism linking complement risk loci to disease-specific gene expression patterns in AD.

In addition to DNA methylation, histone modifications are central to epigenetic regulation, influencing gene expression by altering chromatin structure and transcriptional accessibility. Post-translational modifications such as histone acetylation (e.g., H3K27ac) and methylation (e.g., H3K27me3) act as dynamic markers of gene activity or repression [339, 340]. In AD, aberrant patterns of histone modifications have been observed, particularly in glial cells, and are believed to contribute to neuroinflammation and immune dysregulation [341]. Direct evidence of histone-mediated regulation of complement genes in AD is limited but growing. Notably, a histone acetylome-wide association study by Marzi and colleagues identified differential H3K27ac enrichment in the entorhinal cortex of AD patients, including at the *CR1* locus [342]. These acetylation changes were associated with altered gene expression, supporting a role for histone modifications in modulating complement activity in AD. *CLU* has also been shown to be responsive to chromatin state. In retinal pigment epithelial cells, histone deacetylase (HDAC) inhibition upregulated *CLU* expression, indicating that increased histone acetylation can promote *CLU* transcription [336]. Similarly, Nuutinen et al. demonstrated that treatment with various HDAC inhibitors induced *CLU* mRNA and protein expression in neural cells, and that this effect was further enhanced by co-treatment with a DNA methylation inhibitor, highlighting a synergistic relationship between histone acetylation and DNA methylation in the transcriptional regulation of *CLU* [335]. More broadly, enhancer remodeling has been implicated in immune gene regulation in AD. Gjoneska et al. reported conserved enhancer signatures marked by H3K27ac and H3K4me1 in microglia from mouse models of neurodegeneration, with overlapping patterns in human AD cortex [341]. These enhancer landscapes were enriched for immune and phagocytic genes, providing indirect support for complement-associated regulation [341]. Extending this work, Nott et al. demonstrated elevated H3K27ac at microglia-specific enhancers in AD brain tissue [343].

Transcriptional regulation plays a central role in the context-dependent expression of complement components in the brain, influencing the epigenetic signature. Transcription factors such as PU.1 (SPI1) and IRF8 are essential for microglial development and immune-related gene expression [344]. While complement genes like *C1qa* are highly expressed in microglia, and PU.1 is known to shape the microglial enhancer landscape [345], direct transcriptional regulation of specific complement components remains to be fully elucidated. Nonetheless, emerging in vivo evidence suggests that PU.1 modulation alters AD-related phenotypes and immune transcriptomes, including complement pathways, further supporting its role in disease-context regulation [346].

MicroRNAs (miRNAs), a class of non-coding RNAs, are considered epigenetic regulators owing to their ability to post-transcriptionally silence gene expression without altering the underlying DNA sequence [347]. They are increasingly recognized as modulators of immune and complement-related pathways in AD. Among them, miR-146a is one of the most consistently upregulated in AD brain and cellular models, acting in an NF-κB-dependent manner to suppress inflammatory signaling and directly target *CFH* [348–352] (Table 1). This suppression of *CFH* appears particularly prominent in microglia, where miRNA-146a expression is robustly induced by Aβ42 and TNF-α exposure, as shown in primary human neural and glial cell cultures [353]. In the hippocampal CA1 region of sporadic AD cases, miR-146a and miR-34a were both significantly upregulated and have been shown to downregulate a number of target mRNAs involved in immune and synaptic regulation, including *CFH*, *SHANK3*, and *TREM2*, further implicating miRNA dysregulation in both complement activation and synaptic vulnerability in AD [354]. miR-155, a pro-inflammatory miRNA also implicated in neurodegeneration, has been associated with decreased *CFH* expression and altered glial activation in both AD and Down syndrome brain tissues [355, 356]. Other miRNAs, such as miR-132 and miR-124, have been linked to synaptic regulation and complement-mediated neuroinflammation, including reported interactions with C1q family members and related signaling pathways [357–359]. A recent systematic review and meta-analysis further confirmed the dysregulation of miRNAs including miR-146a, miR-132, and miR-155 across multiple AD brain regions, strengthening their relevance as epigenetic regulators and candidate biomarkers [360]. Recent network-based and pathway analyses have identified additional miRNAs with potential roles in AD-related neuroinflammation and immune regulation, though direct links to complement components remain to be clarified [361]. Broad alterations in miRNA expression profiles have been reported in both AD brain tissue and CSF, further supporting their relevance as upstream regulators and potential biomarkers [362].

Extracellular vesicles may further contribute to miRNA-based complement regulation by facilitating intercellular RNA transfer, including between astrocytes and neurons [363, 364]. These vesicles, particularly exosomes, not only mediate neuroinflammatory signaling but are also emerging as potential therapeutic vehicles and biomarkers in AD [365]. Although less characterized, long non-coding RNAs (lncRNAs) and circular RNAs (circRNAs) are also emerging as potential regulators of neuroimmune signaling and complement gene expression in the aging brain [366]. Intriguingly, studies have also suggested a potential reverse signaling effect, whereby complement proteins such as C1q may influence miRNA expression in neurons, hinting at complex bidirectional communication between complement and non-coding RNA networks [16]. These findings suggest that non-coding RNAs form an epigenetic regulatory layer that may fine-tune complement activity in AD in a context- and cell type–specific manner.

### Integrative models and environmental triggers in complement regulation

Complement activation in AD reflects the convergence of genetic predisposition, epigenetic modulation, and environmental influences. As discussed above, while GWAS-identified variants in complement-related genes such as *CR1* and *CLU* establish foundational susceptibility, these effects are dynamically shaped by epigenetic mechanisms including DNA methylation, histone modifications, and non-coding RNAs. Environmental exposures, such as viral infection, chronic stress, and aging, may trigger or amplify epigenetic remodeling, thereby shifting glial activation thresholds and altering complement expression profiles. For instance, Licastro and colleagues proposed that AD-associated genetic risk loci, including *CR1* and *CLU*, may represent components of an immune gene network that influences the brain’s ability to respond to neurotropic viral infections, particularly herpesviruses, suggesting that viral exposure may act as an environmental trigger interacting with complement-related genetic susceptibility [367].

Recent multi-omics studies have further highlighted how mitochondrial function, epigenetic modifications, and inflammation converge in AD pathogenesis. Zhang et al. employed Mendelian randomization to identify mitochondrial dysfunction-related genes such as *CLU*, *MAPT* (Microtubule Associated Protein Tau), and *ACE* (Angiotensin I Converting Enzyme), whose expression levels are influenced by DNA methylation and are genetically linked to inflammatory cytokines, including IL-17 C and IL-18 [332]. These findings suggest that epigenetic regulation of mitochondrial and nuclear gene expression may jointly influence the inflammatory environment in AD, potentially interacting with complement and immune signaling cascades. In support of this, proteomic analyses have also implicated complement dysregulation in AD progression. Bai et al. identified upregulation of complement components in postmortem brain tissue, with supporting evidence from the 5xFAD mouse model [368].

Sex-based differences in neuroinflammatory processes have also emerged as key modulators of complement activity in AD. Chromosomal and hormonal factors, such as X chromosome inactivation and sex hormone–dependent epigenetic modulation, can shape immune signaling trajectories [369]. Consistent with this, Casali et al. showed that both sex chromosomes and gonadal hormones independently modulate microglial activation and plaque burden in 5xFAD mice, underscoring the complex genetic and hormonal influences on neuroinflammation [370]. Recent single-cell transcriptomic analysis in the THY-Tau22 mouse model revealed sex-dependent dysregulation of complement genes such as *C1qa*, *C1qb*, and *C1qc*, with more pronounced alterations in females [371]. Supporting this, a longitudinal study in 3xTg-AD mice reported male-specific upregulation of complement genes, including *C1qa-c* and *C5ar1*, mirroring findings in postmortem male AD brains [372]. These differences may underlie observed disparities in disease prevalence, progression, and response to treatment between sexes.

In addition to genetic and hormonal factors, lifestyle-related environmental influences such as diet and exercise have been proposed to affect brain health via epigenetic mechanisms. Nutrients like polyphenols, B vitamins, and omega 3 fatty acids have been shown to modulate DNA methylation and histone modifications, potentially impacting neuroimmune signaling and glial responses [373]. Although their effects on complement regulation remain to be clarified, these findings support the broader view that environmental exposures can shape the epigenetic landscape in AD. Diet may also directly impact complement-mediated neuroinflammation. A recent study demonstrated that even a short-term high-fat diet impaired memory and enhanced synaptic degradation in 3xTg-AD mice via a complement-dependent mechanism, highlighting the rapid sensitivity of complement pathways to environmental triggers [374]. Similarly, Poxleitner and colleagues found that a Western diet disrupted glucose and fatty acid metabolism and induced adaptive immune responses in the brain of APP/PS1 mice, emphasizing the role of diet in modulating brain homeostasis [375]. Epidemiological studies have also supported gene-diet interactions in AD risk, with evidence suggesting that protective effects of omega 3 fatty acid intake may be modulated by APOE genotype and complement-related genes such as *CLU* and *CR1* [376].

Beyond dietary influences, physical exercise appears to exert protective effects against complement-mediated neuroinflammation. Remarkably, plasma from exercising mice was shown to reduce neuroinflammation and improve cognition in AD models, an effect linked to increased levels of *CLU* [377]. Yang et al. reported that aerobic exercise regulates the GPR81 signaling pathway (a lactate-sensitive GPCR pathway) and maintains complement-microglia axis homeostasis, promoting synaptic protection in early-stage AD [378]. Li et al. demonstrated that physical exercise decreases complement-mediated synaptic loss and protects against cognitive impairment by inhibiting microglial Tmem9-ATP6V0D1 signaling [218]. Moreover, combining exercise with postbiotic treatment resulted in synergistic improvements in mitochondrial function and reduced AD-related gene expression, suggesting a multifaceted approach to mitigating neuroinflammation [379].

Together, these studies reinforce the need for integrative models of complement dysregulation that extend beyond static genetic risk to include dynamic, context-sensitive modifiers. Multi-omic approaches combining genetics, epigenomics, transcriptomics, proteomics, and exposome data will be critical for unraveling the complex regulatory networks underlying complement activity in the aging and diseased brain. Understanding how factors such as age, sex, lifestyle, and systemic inflammation converge on the regulation of key complement genes, including *CR1*,* CLU*, and others, will be essential to define risk trajectories and identify precision-targeted interventions. As research moves forward, emphasis should be placed on cell type-specific regulatory dynamics and longitudinal modeling to fully capture the evolving landscape of complement biology in AD.

**Table 1:** Genetic and epigenetic regulation of complement in AD

Gene	Type of Regulation	Mechanism	Proposed Role in AD	CNS Expression
***CLU*** [291, 333, 335] (Clusterin or ApoJ)	Genetic, Epigenetic, Environmental Factors [7, 9, 290, 329–332, 337, 367, 376, 377]	GWAS risk locus [7,9,290]; methylation [329–332, 337]	Chaperone for Aβ [292]; regulator of MAC formation [293, 294]; modulates glial inflammation and BBB function [294–296]; context-dependent neuroprotection [294–301]	Astrocytes (primary), neurons, microglia, endothelial cells [294]
***CR1*** (Complement Receptor 1)	Genetic, Epigenetic, Environmental Factors [8, 9, 302–311, 317, 329, 330, 367, 376]	GWAS risk locus (multiple SNPs and structural variants identified) [8, 9, 302–311, 316, 317]; methylation [329, 330]; histone acetylation [342]	Mediates immune complex clearance via C3b/C4b binding [8, 309, 311]; modulates amyloid burden and neuroinflammation [312, 313]; regulates microglial phagocytosis [178]; associated with depression [314]	Minimal in CNS parenchyma, primarily expressed in peripheral immune cells [177, 310, 311, 315]
***CFH*** (Complement Factor H)	Genetic, Epigenetic [319–321, 348–351, 353, 354, 356]	Suggestive genetic association [319–321]; suppression by miR-125b, miR-146a, miR-155 [348–351, 353, 354, 356]	Downregulated by miRNAs [348–351, 353, 354, 356]; impaired regulation exacerbates complement-mediated neuroinflammation [349, 353, 356]	Microglia (primary), astrocytes (moderate, inducible in AD), neurons (low) [349, 350]
***C1S*** (Complement component 1 subcomponent S)	Limited genetic evidence [204]	Classical pathway serine protease	Mediator of classical pathway activation; AD relevance under investigation	Low in brain, primarily expressed in liver

## Gut-complement link in AD neuroinflammation

The gut microbiome is a complex ecology composed of billions of microorganisms, predominantly bacteria, that live in the intestinal lining [380]. Emerging evidence suggests that the microbiome in the intestinal lining plays a major role in modulating the immune response in health and disease and, as such, can also be involved in neuroinflammatory and neurodegenerative conditions such as AD [381]. Complement proteins such as C3 and factor B, along with receptors like C3aR and C5aR, are present in the mucosal and epithelial tissues of the intestine under normal conditions, and also influence the immune response and contribute to the brain-gut axis communication relevant to AD [86, 382]. This intestinal homeostasis is partially maintained by the presence of complement regulators CD46, CD59, and CD55 on the apical and basolateral sides of the intestinal epithelial cells, secreted by the mucosal cells [383, 384], and their dysregulation can contribute to systemic inflammation, a known risk factor of AD. The exact role of intestinal complement activity in AD is not yet fully understood. However, these findings highlight the necessity of exploring gut–complement–brain interactions within the framework of neurodegeneration.

Emerging evidence suggests that bidirectional communication between the gut microbiome and CNS through various pathways, including immune, entero-endocrine, vagus nerve, and microbial metabolic pathways [385–387], is influenced by microbial dysbiosis caused by factors like LPS, aging, diet, drug intake, and diseases [388, 389]. Disruption of gut immune homeostasis caused by microbial dysbiosis can result in increased levels of complement proteins and TLRs, leading to heightened proinflammatory cytokines in the systemic circulation [390–392]. The integrity of the BBB can be disrupted by these elevated proinflammatory cytokines and facilitate neuroinflammation, which is one of the hallmarks of AD [390–392]. In addition, recent studies have highlighted that specific interventions, such as dietary intake, prebiotics/probiotics, fecal microbiota transplantation, as well as factors like age and sex, can modulate the composition of the microbiome, which can influence neuroinflammation and the pathogenesis of AD [393–395].

In 5xFAD mice, fecal transplants from healthy mice (C57BL/6) dramatically improved cognitive function, lower neuroinflammation, and lessened the pathology of AD. On the other hand, in healthy mice, fecal microbiota transplants from both 5xFAD and healthy mice worsened systemic inflammation and cognitive impairments [396]. Hao and his team found that germ-free mice exhibited elevated serum C3 and C3/C3R signaling after receiving a microbiome from the feces of mice with chronic unpredictable mild stress (CUMS), which resulted in abnormal microglia-dependent synapse pruning and signs of depression in those mice [370]. This result signifies that the intestinal microbial components can influence both peripheral and central complement systems, which play a major role in the pathogenesis of neurological diseases like AD. Research indicates that the maturation, regulation, and mitochondrial function of microglia are regulated by short-chain fatty acids (SCFAs) secreted by the gut microbiome under homeostatic conditions [397–399]. Prebiotic and probiotic intake increases SCFAs in the gut microbiome and plays an important role in neuroprotective effects [400]. It has been shown that early intervention with sodium butyrate in 5xFAD mice inhibits Aβ deposition [401, 402]. However, it is very intriguing to note that in the germ-free AD mouse model, SCFAs have detrimental effects by inducing glial activation and worsening Aβ pathology [403]. These mixed findings indicate the more beneficial and physiological balance effect of endogenous SCFAs produced by gut microbiome than the external administered SCFAs. Furthermore, after LPS treatment, germ-free mice showed deficits in their microglial cell maturation and functioning, and after SCFAs supplements, their microglia were able to recover and mature [398, 404]. Altogether, these studies emphasize how crucial the gut microbiome is for keeping balance and ensuring glial cells function properly in the brain, considering that dysfunctional microglia and aberrant complement activation contribute to AD pathology [405].

**Fig. 2:**
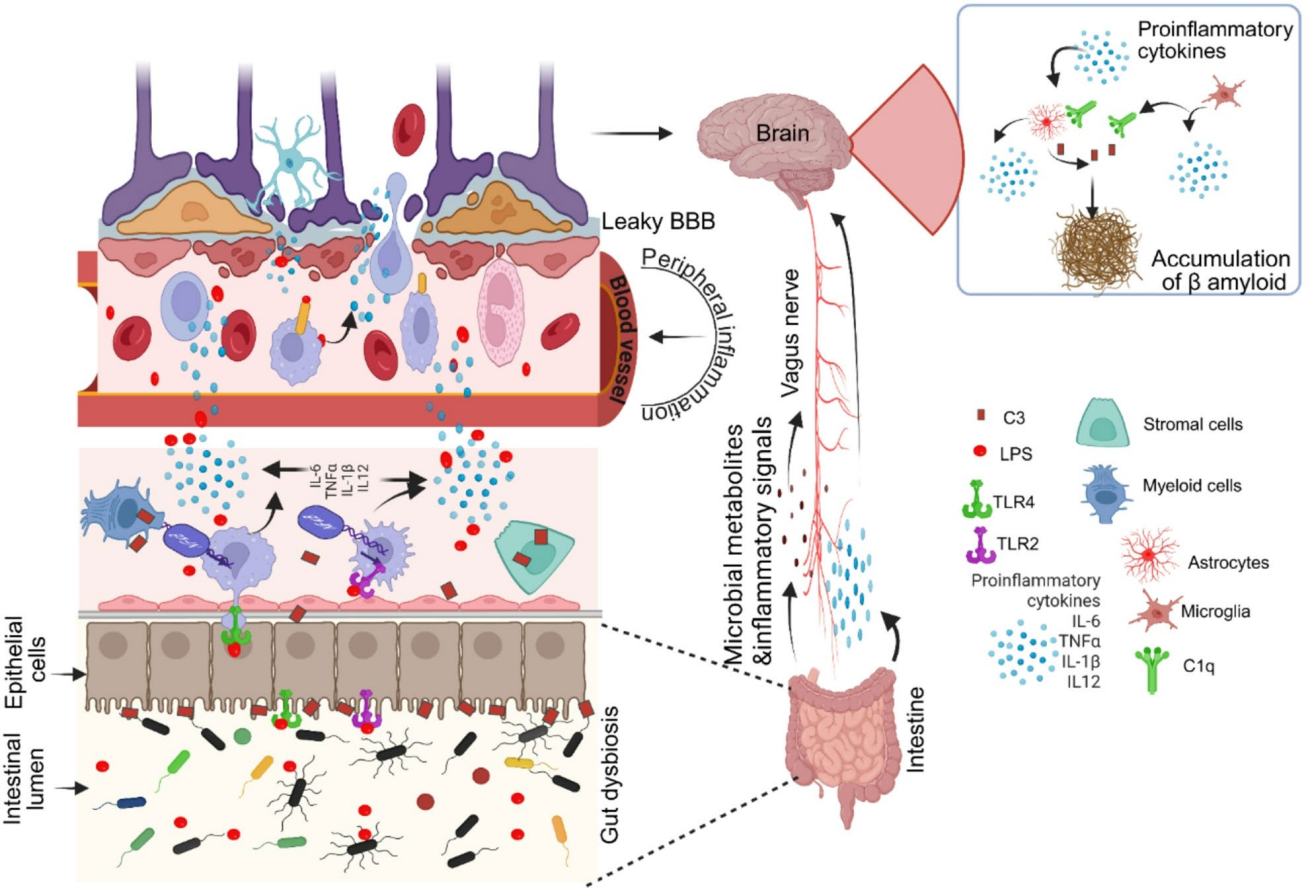
Schematic representation of gut-brain axis-mediated complement dysregulation and neuroinflammation in Alzheimer’s disease. Gut dysbiosis results in the disruption of the intestinal barrier, which is linked to altered complement C3 signaling and an increased release of bacterial lipopolysaccharides (LPS) and metabolites into the bloodstream. These factors cause widespread inflammation by activating immune cells and increasing proinflammatory cytokines (like IL-6, TNF-α, IL-1β, IL-12), which may compromise the blood-brain barrier (BBB). Simultaneously, the compromised BBB permits peripheral inflammatory signals to infiltrate the brain, resulting in the activation of microglia and increasing the production of C1q, which subsequently activates the astrocytes and thereby exacerbates neuroinflammation. The inflammatory environment facilitates or hastens the accumulation of amyloid-β (Aβ), a defining characteristic of Alzheimer’s disease pathology. Figure created with Biorender.com

In the brain, astrocytes and microglia are the main sources of C3 and C1q components of the complement system [388, 389]. The sustained presence of dysbiotic bacteria has the potential to disrupt the homeostasis of the gut microbiome, leading to gut leakiness (Fig. 2). This condition makes the metabolite LPS enter into blood circulation, thereby triggering systemic inflammation, eventually resulting in neuroinflammation within the brain [390–392]. The activation of the complement pathway, particularly the release of C3 from activated astrocytes, may further stimulate microglial activity, promoting the release of C1q and various cytokines [406, 407]. Such processes can exacerbate Aβ toxicity and plaque formation while also activating macrophages, which may contribute to synaptic loss and neurodegeneration, characteristic of neurodegenerative disorders such as AD [251, 408].

A growing body of studies recently reported the alteration of the gut microbiome in AD patients compared with healthy non-AD individuals. For example, the bacteria *Alistipes*,* Actinomyces viscosus*,* Bacteroides*,* Bilophila*,* Clostridium_XlVa*,* Flavobacterium*,* Lactobacillus*,* Moheibacter*,* Neorhizobium*,* Phascolarctobacterium*,* Solobacterium*, and *Weissella* were found to be more common in people with AD and less common in healthy individuals [409, 410]. In addition, recent studies also highlighted the age- and sex-specific difference in microbiome modulating the neuroimmune response towards AD pathology [395, 411]. Beneficial gut microbiota such as *Firmicutes* and *Actinobacteria*, including *Bifidobacterium* spp., *Blautia* spp., and members of *Lachnospirales*, were predominant in people below 75 years of age, while people above 75 years of age displayed a microbial community enriched in *Bacteroidota*, particularly *Bacteroidia* and *Bacteroides* spp., known to be associated with proinflammatory cytokines [412]. LPS and similar molecules can damage the gut barrier and enter the bloodstream. These microbial products are detected by pattern recognition receptors (PRRs), like TLRs, and can trigger the complement system, especially the alternative and lectin pathways, resulting in inflammation throughout the body and in the brain. Alterations in the gut microbiome associated with AD have also been observed in AD mouse models, including 5XFAD mice, which exhibit distinct fecal microbiome profiles after 9 months [412] and in 3xTg mice at 12 weeks of age [413] in comparison to normal wild-type mice. These distinct microbial profiles in two different studies might be due to differences in strain, diet and environmental exposure. Another important longitudinal study also highlighted the significant influence of distinct microbiome composition including reduced *Turicibacter* abundance (a potential mediator of gut-brain axis) and altered serotonin metabolism in 5xFAD mice indicative of gut-driven dysregulation of signaling molecules and their potential influence on AD [414]. Liang et al. demonstrated that different AD mouse models at different ages have different microbiome compositions, which shows how these microbiomes have the potential role in exacerbating amyloid burden in these models [415].

Certain dysbiotic Gram-negative bacteria generate extracellular proteins resembling amyloid, and elicit inflammatory reactions similar to those caused by amyloid aggregates in the CNS [416–418]. These secreted amyloid-like proteins can contribute to activation of the immune response and complement pathways [416, 417]. Long-term and ongoing disruption of gut microbiome integrity results in the continuous activation of the complement pathway, which may influence amyloid aggregation and contribute to the pathogenesis of AD.

## Biomarkers indicate complement system dysregulation in AD

AD is defined by pathological features including the accumulation of amyloid plaques, tau protein aggregation, synaptic degeneration, and cognitive decline [419, 420]. According to the Alzheimer’s Association, 7.2 million Americans over 65 have AD dementia in 2025, and this number is expected to grow to more than 13.8 million individuals by 2060 [421]. A study published in Lancet in 2018 suggested that amyloid plaques begin to form at least 22 years before AD symptoms show up, and tau protein tangles start to develop 20 years after the amyloid plaques accumulate, before mature tau tangles form in AD [422]. Additionally, glucose hypometabolism and brain atrophy start 18 and 13 years before the onset of clinical symptoms becomes evident, respectively [423, 424].

Before 2012, postmortem imaging of Aβ buildup and tau tangles was considered a criterion for diagnosing AD. In 2012, the National Institute on Aging (NIA) - Reagan Institute formulated the diagnostic criteria for AD, which involves a substantial accumulation of amyloid and tau in the brain [425]. The AT(N) system, proposed by the NIA–Alzheimer’s Association (NIA-AA), categorizes AD biomarkers into three primary groups: A (amyloid deposition, e.g., CSF/plasma Aβ42, amyloid PET), T (tau pathology, e.g., CSF/plasma p-tau, tau PET), and N (neurodegeneration, e.g., total tau, NfL, GFAP, FDG-PET, MRI) [426–428]. Currently, CSF and blood-based biomarkers are gaining attention as accessible and minimally invasive tools for the detection and monitoring of AD [429, 430]. Aβ42/Aβ40, p-tau, and NfL can be detected in blood samples of AD patients and are now considered potent blood-based biomarkers [431, 432].

Recent evidence suggests that the biomarkers of the complement system may have added value in diagnosing AD, by revealing neuroinflammatory processes that A, T, or N biomarkers do not completely show. Specifically, complement proteins such as C1q, C3, C4, and their activated forms, exhibit alterations in AD and are detectable in blood plasma, offering a convenient method for assessing neuroinflammation [433, 434]. The identification of significant complement components in blood may be beneficial for early screening and large-scale population research in AD. Higher levels of C1q, C3a, C4d, and factor B in the blood are also associated with memory issues, amyloid accumulation, and the progression from MCI to AD [184, 435, 436]. Complement dysregulation in AD is further supported by human biomarker studies of CSF and plasma, with consistent changes in C3, C4, C1q, TCC, and Factor H confirming the translational significance of complement-targeted mechanisms [176, 321, 437]. Studies have found a significant elevation of plasma clusterin in patients with AD compared to the control group [438–440]. This signifies that clusterin, one of the complement analytes, may have potential as an AD biomarker. Overall, complement activation products and inhibitors show potential as biomarkers that could complement the AT(N) profile and serve as indicators of disease progression and treatment response.

## Complement and complement regulatory proteins as potential therapeutic targets

As discussed in the previous sections, complement activation plays a central role in vascular disorders and neurodegenerative diseases such as AD and CAA. While the underlying pathology and triggers vary, inflammation is a common factor, and particularly dysregulated activation of complement cascade is a common driver of cellular damage [441]. Therefore, targeting complement cascade either to upregulate or downregulate the effector function presents a therapeutic opportunity.

In early disease stages of AD or CAA, the complement pathway can exert protective effects by clearing debris, removing antigen-immune complexes, facilitating immune cell trafficking and supporting cell signalling. However, as the disease worsens, excessive complement activation can contribute to brain and vascular injury. This duality underscores the need for stage-specific therapeutic strategies based on disease progression as no single complement-targeted therapy is likely to be effective across all disease stages. Given the chronic nature linked to their progressive pathology, AD and CAA may require long-term modulation of complement activity due to sustained low-grade activation over many years [19]. Importantly, because the complement pathway plays a significant role in the defense against microbes and infections, blocking the complement cascade carries risks of infection or weakened immunity. Histochemical and genetic data [20, 442] have confirmed the role of complement in driving inflammation during the development and course of AD. Therefore, identifying reliable complement biomarkers could aid in tracking disease progression from the early diagnosis of MCI to AD, supporting disease stratification and patient selection [440, 442]. This may help to determine the optimal time window for complement-targeted interventions [442].

Multiplex assays have identified increased levels of clusterin in plasma, differentiating AD from controls, and modeling with relevant co-variables indicated clusterin, Complement Factor I, and MAC to be different in MCI-converters from non-converters, while plasma Complement Factor I levels were shown to be associated with brain atrophy [440]. These markers underscore the importance of predicting disease progression, identifying the right complement target and optimizing the therapeutic window. Also, understanding which complement proteins to target, including upstream (C1q, C1s, C4), central (C3, C3a/C3aR), and downstream (C5a/C5aR, C5-C9) or their regulators is also crucial [443, 444].

In the context of the complex pathology of AD, thus far, there are no complement-based therapeutics available. Most of the data investigating potential therapeutic interventions comes from animal models of amyloidosis and tauopathy, where modulating the complement cascade yielded neuroprotective effects. A sampling of the studies categorized as per the complement target are referred to in Table 2. In AD mouse models, intracranial administration of antisense oligonucleotides directed against C1r, C1s and C4 showed neuroprotection and rescued spine loss in the PS2APP amyloid mouse model [204], highlighting the use of nucleic acid-based medicine as a potential therapeutic approach for neurodegenerative diseases. C5aR1 antagonism with a cyclic hexapeptide, PMX205, also decreased amyloid load by 49–62%, and glial activation by 42–68% in AD mouse models [237]. In another AD mouse model, i.e. making use of APP^NL−G−F^ mice, both C7 deficiency and C3 deletion were shown to protect against spine loss and improve cognition [179, 246]. In models of tauopathy, deletion of C3aR and C3 inhibition ameliorated tau pathology and lowered inflammation and neuronal loss [179, 222]. A clone 73D1 recombinant monoclonal antibody, a strong inhibitor for C7, fused with nanobody Nb62 targeting the transferrin receptor, was shown to penetrate the BBB and further reduce complement activation, synapse loss and amyloid levels in the APP^NL−G−F^ mouse model [247].

Overall, these studies suggest that complement and its regulatory proteins serve as promising therapeutic targets in AD. Notably, there are several commercially available complement therapeutics that have demonstrated efficacy in other human diseases as shown in Table 3. Drug repurposing to lower complement activation in neurodegenerative diseases may hold potential, but, further studies are required to assess their efficacy and potential side effects.

**Table 2:** Drug development targeting complement in AD mouse models

Therapeutic Target	Disease Model	Intervention	Key Outcomes
C1r, C1s, C4	Amyloidosis (PS2APP)	Antisense Oligonucleotides [204]	Significant rescue of synapse loss
C5aR	Amyloidosis (Tg2576); Amyloid and Tau (3xTg)	PMX205 (C5a receptor peptide antagonist) [111, 237, 244]	Reduced amyloid deposits by 49–62%, glia by 42–68% and improved cognitive function
C3aR	Amyloidosis (APP/TTA)	SB290157 (C3aR antagonist) [258]	Reduced amyloid load, microgliosis
Membrane Attack Complex	Amyloidosis (APP^NL−G−F^)	mAb 73D1 (C7 blocker) [247]	Reduced synapse loss, amyloid burden, improved cognition

**Table 3:** Complement therapeutics currently available for other indications

Complement drugs	Approved drugs/class	Disease	Pathology	Symptoms
Plasma-derived C1 esterase inhibitors (C1INH)	Cinryze™; Berinert™ (Biologics)	Hereditary angioedema [445]	Deficiency or dysfunction of complement C1 inhibitor	Episodes of swelling in hands, feet, face, airways, intestines
C1s inhibitor	Sutimlimab (Monoclonal antibody)	Cold agglutinin disease [446]	IgM auto antibodies targeting RBCs causing hemolysis	Cold induced symptoms including anemia, pallor, muscle weakness
C3 inhibitor	Pegcetacoplan (small peptide inhibitor)	PNH-Paroxysmal nocturnal hemoglobinuria (intra & extravascular hemolysis) [447] Age-related macular degeneration, [448]	Lack of regulatory proteins CD55 and CD59 on the surface of red blood cells (RBCs) causes complement activation and hemolysis Overactivation of complement system int the eye causing subretinal deposits, death of retinal pigment epithelial cells	Hemoglobinuria (hemoglobin in the urine) Central vision loss
C5 inhibitor	Eculizumab™ (Monoclonal antibody)	PNH-Paroxysmal nocturnal hemoglobinuria (intravascular hemolysis) [449] Aquaporin-4–positive Neuromyelitis optica spectrum disorder (NMOSD) [450]	Hemolysis Complement (MAC) deposition around blood vessels	Hemoglobinuria Severe optic neuritis and transverse myelitis causing vision loss, paralysis, and bladder/bowel dysfunction
C5 inhibitor	ULTOMIRIS^®^ (ravulizumab-cwvz) ; anti-C5 IgG2/4 (Monoclonal antibody)	generalised myasthenia gravis (gMG) [451] atypical haemolytic uraemic syndrome (aHUS) [452] aquaporin-4–positiveNeuromyelitis optica spectrum disorder (NMOSD) [449, 451]	Activation of the complement system at the neuromuscular junction Dysregulation of the complement leading to blood vessel damage Complement (MAC) deposition around blood vessels	Muscle weakness, fatigue kidney damage, blood clots, and anemia Severe optic neuritis and transverse myelitis causing vision loss, paralysis, and bladder/bowel dysfunction
C5 inhibitor	Pozelimab (Monoclonal antibody) Crovalimab (Monoclonal antibody) Avacinacptad pegol (RNA aptamer)	CD55-deficient protein-losing enteropathy (PLE) or CHAPLE disease [453] PNH [454] Geographic atrophy (advanced age related macular degeneration)	Mutations in CD55 gene causing overactivation of complement system causing damage to blood, lymph vessels along upper digestive tract Hemolysis Excessive complement activation in the eye	Enteropathy Hemoglobinuria Central vision loss, reduced color perception
C5 inhibitor	Zilucoplan (macrocylic peptide)	generalised myasthenia gravis (gMG) in patients who are anti-AChR antibody positivity [455]	Activation of the complement system at the neuromuscular junction	Muscle weakness, fatigue
C5a inhibitor	Vilobelimab (Monoclonal antibody)	COVID-19, acute respiratory distress syndrome [456]	Swelling in the lungs	Difficulty in breathing
C5aR1 inhibitor	Avacopan (small molecule)	Polyangiitis [457]	Autoimmune disease with anti-neutrophil cytoplasmic autoantibody associated vasculitis	Swelling and irritation in blood vessels
Factor B inhibitor	Iptacopan (small molecule)	PNH [458]	Intravascular & extravascular hemolysis	Hemoglobinuria
Factor D inhibor	Danicopan (small molecule)	PNH [459]	Extravascular hemolysis	Hemoglobinuria

## Future directions

As our understanding of complement biology in AD deepens, future research should prioritize clarifying the cell type- and brain region-specific roles of complement components across disease stages. Longitudinal multi-omic studies integrating genomics, epigenomics, proteomics, spatial transcriptomics, and the exposome will be essential for mapping temporal dynamics and functional outcomes. Further efforts should investigate sex-specific regulatory mechanisms, the interplay between peripheral and CNS complement systems, especially at barrier sites like the BBB and choroid plexus, and the influence of systemic factors such as microbiota and metabolism. Functional validation of GWAS and EWAS findings using iPSC-based model systems and CRISPR-based tools remains a key priority. Moreover, expanding our knowledge of intracellular complement signaling may uncover novel immunometabolic interactions. Therapeutically, identifying context-sensitive complement targets that can be engaged in combination with strategies supporting synaptic resilience and modulating microglial responses in late-stage AD models [460], may offer synergistic benefits. Finally, the discovery of reliable AD subtype- and stage-specific diagnostic and prognostic biomarkers of complement activity will be critical for advancing precision complement-modulating interventions in AD.

## Conclusion

The complement system has emerged as a central player in the pathogenesis of AD, exerting diverse and context-dependent effects on synaptic function, neuroinflammation, and neurodegeneration. While genetic studies have highlighted key susceptibility loci such as *CR1* and *CLU*, epigenetic and environmental factors increasingly appear to modulate complement activity in cell type-, sex-, and disease stage-specific ways. From developmental synaptic pruning to maladaptive activation in the aging and diseased brain, complement signaling represents a dynamic and multi-layered process. Advances in spatial transcriptomics, iPSC modeling, and systems-level analyses are now revealing a more nuanced view of complement as a regulator of both CNS and peripheral immune networks. Unraveling these complexities will be essential for the development of precision therapies aimed at harnessing the beneficial roles of complement while curbing its deleterious consequences in AD.

## Data Availability

No datasets were generated or analysed during the current study.

## Abbreviations

Aβ: Amyloid-β
ACE: Angiotensin Iconverting enzyme
AD: Alzheimer’s disease
ALS: Amyotrophic lateral sclerosis
AP-1: Activator protein 1
Apo E: Apolipoprotein E
Apo J: Apolipoprotein J
ARIA: Amyloid-related imaging abnormalities
AT(N): Amyloid, Tau, and Neurodegeneration biomarker classification system
BBB: Blood–brain barrier
CAA: Cerebral amyloid angiopathy
CD: Cluster of differentiation
CFH: Complement factor H
circRNAs: circular RNAs
CLU: Clusterin (also known as Apo J)
C3aR: Complement C3a receptor
C5aR: Complement C5a receptor
CNS: Central nervous system
CpG: Cytosine-phosphate‐Guanine
CRISPR: Clustered Regularly Interspaced Short Palindromic Repeats
CR1: Complement receptor 1
CR3: Complement receptor 3
CR4: Complement receptor 4
Crry: Complement receptor 1-related gene/protein y
CSF: Cerebrospinal fluid
CSMD1: CUB and Sushi Multiple Domains 1
CSMD3: CUB and Sushi Multiple Domains 3
CUMS: Chronic unpredictable mild stress
DAMPs: Damage-Associated Molecular Patterns
EWAS: Epigenome-wide association study
GZMK: Granzyme K
GWAS: Genome-wide association study
HD: Huntington’s disease
HDAC: histone deacetylase
HSF: heat-shock factor
iC3b: Inactivated C3b
IL-1α: Interleukin-1 alpha
IL-1β: Interleukin-1 beta
IL-6: Interleukin-6
IL-12: Interleukin-12
IL-17C: Interleukin-17C
IL-18: Interleukin-18
iMGLs: induced pluripotent stem cell-derived microglia-like cells
iPSC: induced pluripotent stem cell
lncRNAs: long non-coding RNAs
LOAD: late-onset Alzheimer's disease
LPS: Lipopolysaccharides
LTP: Long-term potentiation
MAC: Membrane attack complex
MAPT: Microtubule Associated Protein Tau
MASP: Mannose-binding serine protease
MAVS: Mitochondrial antiviral signaling protein
MBL: Mannose-binding lectin
MCI: Mild cognitive impairment
MEGF10: Multiple EGF-like domains 10
MERTK: MER Proto-Oncogene, Tyrosine Kinase
mGluR: Metabotropic glutamate receptor
miRNAs: microRNAs
MRI: Magnetic resonance imaging
mTOR: mechanistic target of rapamycin
NF-κB: Nuclear factor kappa B
NFT: Neurofibrillary tangle
NIA-AA: National Institute on Aging–Alzheimer’s Association
NLRP3: Nod-like receptor family pyrin domain containing 3
Nptx2: Neuronal pentraxin 2
PD: Parkinson’s disease
PET: Positron Emission Tomography
PICALM: Phosphatidylinositol binding clathrin assembly protein
PRRs: Pattern recognition receptors
p-Tau: Phosphorylated tau
SCFAs: Short-chain fatty acids
SIRPα: Signal Regulatory Protein alpha
SNPs: Single nucleotide polymorphisms
SPP1: Secreted phosphoprotein 1/osteopontin
SRPX2: Sushi Repeat Containing Protein X-linked 2
SUSD4: Sushi Domain Containing 4
TAM: Tyro3/Axl/MerTK
TBI: Traumatic brain injury
TCC: Terminal complement complex
TLR: Toll-like receptor
Tmem9: Transmembrane protein 9
TNF-α: Tumor necrosis factor-alpha
TREM2: Triggering receptor expressed on myeloid cells 2
TYROBP: Tyrosine kinase binding protein
VCID: Vascular contributions to cognitive impairment and dementia

## References

[1] Zhang W, Xiao D, Mao Q, Xia H. Role of neuroinflammation in neurodegeneration development. Signal Transduct Target Ther. 2023;8:267. 37433768 10.1038/s41392-023-01486-5 PMC10336149

[2] Vijayan M, Reddy PH. Stroke, vascular dementia, and alzheimer’s disease: molecular links. J Alzheimers Dis. 2016;54:427–43. 27567871 10.3233/JAD-160527 PMC5793908

[3] Golde TE. Alzheimer’s disease – the journey of a healthy brain into organ failure. Mol Neurodegeneration. 2022;17:1–19. 10.1186/s13024-022-00523-1 PMC8898417 35248124

[4] Young-Pearse TL, Lee H, Hsieh Y-C, Chou V, Selkoe DJ. Moving beyond amyloid and Tau to capture the biological heterogeneity of alzheimer’s disease. Trends Neurosci. 2023;46:426–44. 37019812 10.1016/j.tins.2023.03.005 PMC10192069

[5] Zheng Q, Wang X. Alzheimer’s disease: insights into pathology, molecular mechanisms, and therapy. Protein Cell. 2025;16:83–120. 38733347 10.1093/procel/pwae026 PMC11786724

[6] Kunkle BW, Grenier-Boley B, Sims R, Bis JC, Damotte V, Naj AC, et al. Genetic meta-analysis of diagnosed alzheimer’s disease identifies new risk loci and implicates Aβ, tau, immunity and lipid processing. Nat Genet. 2019;51:414–30. 30820047 10.1038/s41588-019-0358-2 PMC6463297

[7] Harold D, Abraham R, Hollingworth P, Sims R, Gerrish A, Hamshere ML, et al. Genome-wide association study identifies variants at CLU and PICALM associated with alzheimer’s disease. Nat Genet. 2009;41:1088–93. 19734902 10.1038/ng.440 PMC2845877

[8] Brouwers N, Van Cauwenberghe C, Engelborghs S, Lambert J-C, Bettens K, Le Bastard N, et al. Alzheimer risk associated with a copy number variation in the complement receptor 1 increasing C3b/C4b binding sites. Mol Psychiatry. 2012;17:223–33. 21403675 10.1038/mp.2011.24 PMC3265835

[9] Lambert J-C, Heath S, Even G, Campion D, Sleegers K, Hiltunen M, et al. Genome-wide association study identifies variants at CLU and CR1 associated with alzheimer’s disease. Nat Genet. 2009;41:1094–9. 19734903 10.1038/ng.439

[10] Schartz ND, Tenner AJ. The good, the bad, and the opportunities of the complement system in neurodegenerative disease. J Neuroinflammation. 2020;17:354. 33239010 10.1186/s12974-020-02024-8 PMC7690210

[11] Elvington M, Liszewski MK, Atkinson JP. Evolution of the complement system: from defense of the single cell to guardian of the intravascular space. Immunol Rev. 2016;274:9–15. 27782327 10.1111/imr.12474 PMC5108576

[12] Morgan BP, Gommerman JL, Ramaglia V. An outside-in and inside-out consideration of complement in the multiple sclerosis brain: lessons from development and neurodegenerative diseases. Front Cell Neurosci. 2020;14:600656. 33488361 10.3389/fncel.2020.600656 PMC7817777

[13] Carpanini SM, Torvell M, Morgan BP. Therapeutic Inhibition of the complement system in diseases of the central nervous system. Front Immunol. 2019;10:362. 30886620 10.3389/fimmu.2019.00362 PMC6409326

[14] Stevens B, Allen NJ, Vazquez LE, Howell GR, Christopherson KS, Nouri N, et al. The classical complement cascade mediates CNS synapse elimination. Cell. 2007;131:1164–78. 18083105 10.1016/j.cell.2007.10.036

[15] Stephan AH, Barres BA, Stevens B. The complement system: an unexpected role in synaptic pruning during development and disease. Annu Rev Neurosci. 2012;35:369–89. 22715882 10.1146/annurev-neuro-061010-113810

[16] Benoit ME, Tenner AJ. Complement protein C1q-mediated neuroprotection is correlated with regulation of neuronal gene and MicroRNA expression. J Neurosci. 2011;31:3459–69. 21368058 10.1523/JNEUROSCI.3932-10.2011 PMC3080046

[17] Bohlson SS, Tenner AJ. Complement in the brain: contributions to Neuroprotection, neuronal Plasticity, and neuroinflammation. Annu Rev Immunol. 2023;41:431–52. 36750318 10.1146/annurev-immunol-101921-035639

[18] Donado CA, Theisen E, Zhang F, Nathan A, Fairfield ML, Rupani KV, et al. Granzyme K activates the entire complement cascade. Nature. 2025;641:211–21. 39914456 10.1038/s41586-025-08713-9 PMC12180478

[19] Tenner AJ, Petrisko TJ. Knowing the enemy: strategic targeting of complement to treat alzheimer disease. Nat Rev Neurol. 2025;21(5):250–64. 40128350 10.1038/s41582-025-01073-y PMC12243624

[20] Zelek WM, Tenner AJ. Complement therapeutics in neurodegenerative diseases. Immunobiology. 2025;230:153089. 40544661 10.1016/j.imbio.2025.153089

[21] Daskoulidou N, Carpanini SM, Zelek WM, Morgan BP. Involvement of complement in alzheimer’s disease: from genetics through pathology to therapeutic strategies. Curr Top Behav Neurosci. 2025;69:3–24. 39455500 10.1007/7854_2024_524

[22] Nimmo J, Byrne RAJ, Daskoulidou N, Watkins LM, Carpanini SM, Zelek WM, et al. The complement system in neurodegenerative diseases. Clin Sci (Lond). 2024;138:387–412. 38505993 10.1042/CS20230513 PMC10958133

[23] Batista AF, Khan KA, Papavergi MT, Lemere CA. The importance of Complement-Mediated immune signaling in alzheimer’s disease pathogenesis. Int J Mol Sci. 2024;25(2):817. 38255891 10.3390/ijms25020817 PMC10815224

[24] Brennan FH, Lee JD, Ruitenberg MJ, Woodruff TM. Therapeutic targeting of complement to modify disease course and improve outcomes in neurological conditions. Semin Immunol. 2016;28:292–308. 27049459 10.1016/j.smim.2016.03.015

[25] Pasinetti GM. Inflammatory mechanisms in neurodegeneration and alzheimer’s disease: the role of the complement system. Neurobiol Aging. 1996;17:707–16. 8892343 10.1016/0197-4580(96)00113-3

[26] Hakobyan S, Harding K, Aiyaz M, Hye A, Dobson R, Baird A, Liu B, Harris CL, Lovestone S, Morgan BP. Complement biomarkers as predictors of disease progression in alzheimer’s disease. J Alzheimers Dis. 2016;54(2):707–16. 27567854 10.3233/JAD-160420

[27] Bordet J. Les leucocytes et les proprietes actives du serum Chez les vaccines. Ann Inst Pasteur. 1895:462–506.

[28] Schmalstieg FC Jr, Goldman AS, Jules Bordet. (1870–1961): a Bridge between early and modern immunology. J Med Biogr. 2009;17:217–24. 10.1258/jmb.2009.009061 20029083

[29] Buchner H. Zur nomenklatur der schutzenden Eiweisskorper. Centr Bakteriol Parasitenk. 1891;10:699–701.

[30] Nesargikar PN, Spiller B, Chavez R. The complement system: history, pathways, cascade and inhibitors. Eur J Microbiol Immunol (Bp). 2012;2:103–11. 24672678 10.1556/EuJMI.2.2012.2.2 PMC3956958

[31] Cavaillon J-M, Sansonetti P, Goldman M. 100th anniversary of Jules bordet’s nobel prize: tribute to a founding father of immunology. Front Immunol. 2019;10:2114. 31572361 10.3389/fimmu.2019.02114 PMC6749103

[32] Ehrlich P, Morgenroth J. Zweite mittheilung. Zweite mittheilung Berliner klinische Wochenschrift. 1899;36:481–6.

[33] Nonaka M, Kimura A. Genomic view of the evolution of the complement system. Immunogenetics. 2006;58:701–13. 16896831 10.1007/s00251-006-0142-1 PMC2480602

[34] Hadding U, Müller-Eberhard HJ. The ninth component of human complement: isolation, description and mode of action. Immunology. 1969;16:719–35. 4182367 PMC1409673

[35] Nilsson U. Separation and partial purification of the sixth, seventh and eighth components of human haemolytic complement. Acta Pathol Microbiol Scand. 1967;70:469–80. 6083390 10.1111/j.1699-0463.1967.tb01315.x

[36] Nilsson UR, Mueller-Eberhard HJ, ISOLATION OF BETA IF-GLOBULIN FROM, HUMAN SERUM AND ITS CHARACTERIZATION AS THE FIFTH COMPONENT OF COMPLEMENT. J Exp Med. 1965;122:277–98. 14316946 10.1084/jem.122.2.277 PMC2138056

[37] Mueller-Eberhard HJ, Biro CE. Isolation and description of the fourth component of human complement. J Exp Med. 1963;118:447–66. 14078003 10.1084/jem.118.3.447 PMC2137649

[38] Pillemer L, Ecker EE. The terminology of the components of complement. Science. 1941;94:437. 17758314 10.1126/science.94.2445.437

[39] Dunkelberger JR, Song W-C. Complement and its role in innate and adaptive immune responses. Cell Res. 2010;20:34–50. 20010915 10.1038/cr.2009.139

[40] Pepys MB. Role of complement in the induction of immunological responses. Transpl Rev. 1976;32:93–120. 10.1111/j.1600-065x.1976.tb00230.x 790690

[41] Pepys MB. Role of complement in induction of antibody production in vivo. Effect of Cobra factor and other C3-reactive agents on thymus-dependent and thymus-independent antibody responses. J Exp Med. 1974;140:126–45. 4545894 10.1084/jem.140.1.126 PMC2139695

[42] Morgan BP, Marchbank KJ, Longhi MP, Harris CL, Gallimore AM. Complement: central to innate immunity and bridging to adaptive responses. Immunol Lett. 2005;97:171–9. 15752555 10.1016/j.imlet.2004.11.010

[43] Carroll MC. The complement system in regulation of adaptive immunity. Nat Immunol. 2004;5:981–6. 15454921 10.1038/ni1113

[44] Ricklin D, Hajishengallis G, Yang K, Lambris JD. Complement: a key system for immune surveillance and homeostasis. Nat Immunol. 2010;11:785–97. 20720586 10.1038/ni.1923 PMC2924908

[45] Mastellos DC, Hajishengallis G, Lambris JD. A guide to complement biology, pathology and therapeutic opportunity. Nat Rev Immunol. 2024;24:118–41. 37670180 10.1038/s41577-023-00926-1

[46] West EE, Kemper C. Complosome - the intracellular complement system. Nat Rev Nephrol. 2023;19:426–39. 37055581 10.1038/s41581-023-00704-1 PMC10100629

[47] Walport MJ. Complement. First of two parts. N Engl J Med. 2001;344:1058–66. 11287977 10.1056/NEJM200104053441406

[48] Dobó J, Kocsis A, Farkas B, Demeter F, Cervenak L, Gál P. The lectin pathway of the complement system-activation, regulation, disease connections and interplay with other (proteolytic) systems. Int J Mol Sci. 2024;25:1566. 38338844 10.3390/ijms25031566 PMC10855846

[49] Liszewski MK, Atkinson JP. Alternative pathway activation: ever ancient and ever new. Immunol Rev. 2023;313:60–3. 36089772 10.1111/imr.13132 PMC9973499

[50] Bubeck D. The making of a macromolecular machine: assembly of the membrane attack complex. Biochemistry. 2014;53:1908–15. 24597946 10.1021/bi500157z

[51] Ho BHT, Spicer BA, Dunstone MA. Action of the terminal complement pathway on cell membranes. J Membr Biol. 2025;258(4):269–304. 40122920 10.1007/s00232-025-00343-6 PMC12313776

[52] Song W-C. Crosstalk between complement and toll-like receptors. Toxicol Pathol. 2012;40:174–82. 22109714 10.1177/0192623311428478

[53] Conway EM. Reincarnation of ancient links between coagulation and complement. J Thromb Haemost. 2015;13(Suppl 1):S121–32. 26149013 10.1111/jth.12950

[54] Zipfel PF, Skerka C. Complement regulators and inhibitory proteins. Nat Rev Immunol. 2009;9:729–40. 19730437 10.1038/nri2620

[55] Morgan BP, Harris CL. Complement, a target for therapy in inflammatory and degenerative diseases. Nat Rev Drug Discov. 2015;14:857–77. 26493766 10.1038/nrd4657 PMC7098197

[56] Holers VM. Complement and its receptors: new insights into human disease. Annu Rev Immunol. 2014;32:433–59. 24499275 10.1146/annurev-immunol-032713-120154

[57] Liszewski MK, Kolev M, Le Friec G, Leung M, Bertram PG, Fara AF, et al. Intracellular complement activation sustains T cell homeostasis and mediates effector differentiation. Immunity. 2013;39:1143–57. 24315997 10.1016/j.immuni.2013.10.018 PMC3865363

[58] Kolev M, Le Friec G, Kemper C. Complement–tapping into new sites and effector systems. Nat Rev Immunol. 2014;14:811–20. 25394942 10.1038/nri3761

[59] Hess C, Kemper C. Complement-mediated regulation of metabolism and basic cellular processes. Immunity. 2016;45:240–54. 27533012 10.1016/j.immuni.2016.08.003 PMC5019180

[60] Kolev M, Kemper C. Keeping it all going-complement Meets metabolism. Front Immunol. 2017;8:1. 28149297 10.3389/fimmu.2017.00001 PMC5241319

[61] Liszewski MK, Elvington M, Kulkarni HS, Atkinson JP. Complement’s hidden arsenal: new insights and novel functions inside the cell. Mol Immunol. 2017;84:2–9. 28196665 10.1016/j.molimm.2017.01.004 PMC5373558

[62] King BC, Renström E, Blom AM. Intracellular cytosolic complement component C3 regulates cytoprotective autophagy in pancreatic beta cells by interaction with ATG16L1. Autophagy. 2019;15:919–21. 30741587 10.1080/15548627.2019.1580515 PMC6526805

[63] Sorbara MT, Foerster EG, Tsalikis J, Abdel-Nour M, Mangiapane J, Sirluck-Schroeder I, et al. Complement C3 drives autophagy-dependent restriction of cyto-invasive bacteria. Cell Host Microbe. 2018;23:644–e525. 29746835 10.1016/j.chom.2018.04.008

[64] Viret C, Rozières A, Duclaux-Loras R, Boschetti G, Nancey S, Faure M. Regulation of anti-microbial autophagy by factors of the complement system. Microb Cell. 2020;7:93–105. 32274388 10.15698/mic2020.04.712 PMC7136756

[65] Li Y, Sha Y, Wang H, He L, Li L, Wen S, et al. Intracellular C3 prevents hepatic steatosis by promoting autophagy and very-low-density lipoprotein secretion. FASEB J. 2021;35:e22037. 34762761 10.1096/fj.202100856R

[66] Le Friec G, Sheppard D, Whiteman P, Karsten CM, Shamoun SA-T, Laing A, et al. The CD46-Jagged1 interaction is critical for human TH1 immunity. Nat Immunol. 2012;13:1213–21. 23086448 10.1038/ni.2454 PMC3505834

[67] Kolev M, Dimeloe S, Le Friec G, Navarini A, Arbore G, Povoleri GA, et al. Complement regulates nutrient influx and metabolic reprogramming during Th1 cell responses. Immunity. 2015;42:1033–47. 26084023 10.1016/j.immuni.2015.05.024 PMC4518498

[68] Arbore G, West EE, Rahman J, Le Friec G, Niyonzima N, Pirooznia M, et al. Complement receptor CD46 co-stimulates optimal human CD8 + T cell effector function via fatty acid metabolism. Nat Commun. 2018;9:4186. 30305631 10.1038/s41467-018-06706-z PMC6180132

[69] Zeng J, Xu H, Huang C, Sun Y, Xiao H, Yu G, et al. CD46 splice variant enhances translation of specific mRNAs linked to an aggressive tumor cell phenotype in bladder cancer. Mol Ther Nucleic Acids. 2021;24:140–53. 33767911 10.1016/j.omtn.2021.02.019 PMC7972933

[70] Kremlitzka M, Nowacka AA, Mohlin FC, Bompada P, De Marinis Y, Blom AM. Interaction of serum-derived and internalized C3 with DNA in human B cells-A potential involvement in regulation of gene transcription. Front Immunol. 2019;10:493. 30941132 10.3389/fimmu.2019.00493 PMC6433827

[71] Paiano J, Harland M, Strainic MG, Nedrud J, Hussain W, Medof ME. Follicular B2 cell activation and class switch recombination depend on autocrine C3ar1/C5ar1 signaling in B2 cells. J Immunol. 2019;203:379–88. 31217324 10.4049/jimmunol.1900276 PMC7299189

[72] Kolev M, West EE, Kunz N, Chauss D, Moseman EA, Rahman J, et al. Diapedesis-induced integrin signaling via LFA-1 facilitates tissue immunity by inducing intrinsic complement C3 expression in immune cells. Immunity. 2020;52:513–e278. 32187519 10.1016/j.immuni.2020.02.006 PMC7111494

[73] Niyonzima N, Rahman J, Kunz N, West EE, Freiwald T, Desai JV, et al. Mitochondrial C5aR1 activity in macrophages controls IL-1β production underlying sterile inflammation. Sci Immunol. 2021;6:eabf2489. 34932384 10.1126/sciimmunol.abf2489 PMC8902698

[74] Benoit ME, Clarke EV, Morgado P, Fraser DA, Tenner AJ. Complement protein C1q directs macrophage polarization and limits inflammasome activity during the uptake of apoptotic cells. J Immunol. 2012;188:5682–93. 22523386 10.4049/jimmunol.1103760 PMC3358549

[75] Baudino L, Sardini A, Ruseva MM, Fossati-Jimack L, Cook HT, Scott D, et al. C3 opsonization regulates endocytic handling of apoptotic cells resulting in enhanced T-cell responses to cargo-derived antigens. Proc Natl Acad Sci U S A. 2014;111:1503–8. 24474777 10.1073/pnas.1316877111 PMC3910597

[76] Martin M, Leffler J, Smoląg KI, Mytych J, Björk A, Chaves LD, et al. Factor H uptake regulates intracellular C3 activation during apoptosis and decreases the inflammatory potential of nucleosomes. Cell Death Differ. 2016;23:903–11. 26768663 10.1038/cdd.2015.164 PMC4832108

[77] Schäfer N, Rasras A, Ormenisan DM, Amslinger S, Enzmann V, Jägle H, et al. Complement factor H-related 3 enhanced inflammation and complement activation in human RPE cells. Front Immunol. 2021;12:769242. 34819935 10.3389/fimmu.2021.769242 PMC8606654

[78] Ishii M, Beeson G, Beeson C, Rohrer B. Mitochondrial C3a receptor activation in oxidatively stressed epithelial cells reduces mitochondrial respiration and metabolism. Front Immunol. 2021;12:628062. 33746964 10.3389/fimmu.2021.628062 PMC7973370

[79] Kulkarni HS, Elvington ML, Perng Y-C, Liszewski MK, Byers DE, Farkouh C, et al. Intracellular C3 protects human airway epithelial cells from stress-associated cell death. Am J Respir Cell Mol Biol. 2019;60:144–57. 30156437 10.1165/rcmb.2017-0405OC PMC6376412

[80] Mahajan S, Jacob A, Kelkar A, Chang A, Mcskimming D, Neelamegham S, et al. Local complement factor H protects kidney endothelial cell structure and function. Kidney Int. 2021;100:824–36. 34139209 10.1016/j.kint.2021.05.033

[81] Friščić J, Böttcher M, Reinwald C, Bruns H, Wirth B, Popp S-J, et al. The complement system drives local inflammatory tissue priming by metabolic reprogramming of synovial fibroblasts. Immunity. 2021;54:1002–e2110. 33761330 10.1016/j.immuni.2021.03.003

[82] Datta D, Leslie SN, Morozov YM, Duque A, Rakic P, van Dyck CH, et al. Classical complement cascade initiating C1q protein within neurons in the aged rhesus macaque dorsolateral prefrontal cortex. J Neuroinflammation. 2020;17:8. 31906973 10.1186/s12974-019-1683-1 PMC6945481

[83] Golec E, Ekström A, Noga M, Omar-Hmeadi M, Lund P-E, Villoutreix BO, et al. Alternative splicing encodes functional intracellular CD59 isoforms that mediate insulin secretion and are down-regulated in diabetic Islets. Proc Natl Acad Sci U S A. 2022;119:e2120083119. 35666870 10.1073/pnas.2120083119 PMC9214515

[84] King BC, Kulak K, Krus U, Rosberg R, Golec E, Wozniak K, et al. Complement component C3 is highly expressed in human pancreatic Islets and prevents β cell death via ATG16L1 interaction and autophagy regulation. Cell Metab. 2019;29:202–e106. 30293775 10.1016/j.cmet.2018.09.009

[85] Yan B, Freiwald T, Chauss D, Wang L, West E, Mirabelli C, et al. SARS-CoV-2 drives JAK1/2-dependent local complement hyperactivation. Sci Immunol. 2021;6:eabg0833. 33827897 10.1126/sciimmunol.abg0833 PMC8139422

[86] Sünderhauf A, Skibbe K, Preisker S, Ebbert K, Verschoor A, Karsten CM, et al. Regulation of epithelial cell expressed C3 in the intestine - Relevance for the pathophysiology of inflammatory bowel disease? Mol Immunol. 2017;90:227–38. 28843904 10.1016/j.molimm.2017.08.003

[87] Satyam A, Kannan L, Matsumoto N, Geha M, Lapchak PH, Bosse R, et al. Intracellular activation of complement 3 is responsible for intestinal tissue damage during mesenteric ischemia. J Immunol. 2017;198:788–97. 27913632 10.4049/jimmunol.1502287

[88] Sünderhauf A, Raschdorf A, Hicken M, Schlichting H, Fetzer F, Brethack A-K, et al. GC1qR cleavage by caspase-1 drives aerobic Glycolysis in tumor cells. Front Oncol. 2020;10:575854. 33102234 10.3389/fonc.2020.575854 PMC7556196

[89] Daugan MV, Revel M, Thouenon R, Dragon-Durey M-A, Robe-Rybkine T, Torset C, et al. Intracellular factor H drives tumor progression independently of the complement cascade. Cancer Immunol Res. 2021;9:909–25. 34039652 10.1158/2326-6066.CIR-20-0787

[90] Daugan MV, Revel M, Russick J, Dragon-Durey M-A, Gaboriaud C, Robe-Rybkine T, et al. Complement C1s and C4d as prognostic biomarkers in renal cancer: emergence of noncanonical functions of C1s. Cancer Immunol Res. 2021;9:891–908. 34039653 10.1158/2326-6066.CIR-20-0532

[91] Portilla D, Xavier S. Role of intracellular complement activation in kidney fibrosis. Br J Pharmacol. 2021;178:2880–91. 33555070 10.1111/bph.15408

[92] Tziastoudi M, Theoharides TC, Nikolaou E, Efthymiadi M, Eleftheriadis T, Stefanidis I. Key genetic components of fibrosis in diabetic nephropathy: an updated systematic review and meta-analysis. Int J Mol Sci. 2022;23:15331. 36499658 10.3390/ijms232315331 PMC9736240

[93] Negro-Demontel L, Maleki AF, Reich DS, Kemper C. The complement system in neurodegenerative and inflammatory diseases of the central nervous system. Front Neurol. 2024;15:1396520. 39022733 10.3389/fneur.2024.1396520 PMC11252048

[94] Coulthard LG, Hawksworth OA, Woodruff TM. Complement: the emerging architect of the developing brain. Trends Neurosci. 2018;41:373–84. 29606485 10.1016/j.tins.2018.03.009

[95] Pan J, Ma N, Yu B, Zhang W, Wan J. Transcriptomic profiling of microglia and astrocytes throughout aging. J Neuroinflammation. 2020;17:97. 32238175 10.1186/s12974-020-01774-9 PMC7115095

[96] Ximerakis M, Lipnick SL, Innes BT, Simmons SK, Adiconis X, Dionne D, et al. Single-cell transcriptomic profiling of the aging mouse brain. Nat Neurosci. 2019;22:1696–708. 31551601 10.1038/s41593-019-0491-3

[97] Clarke LE, Liddelow SA, Chakraborty C, Münch AE, Heiman M, Barres BA. Normal aging induces A1-like astrocyte reactivity. Proc Natl Acad Sci U S A. 2018;115:E1896–905. 29437957 10.1073/pnas.1800165115 PMC5828643

[98] Petrisko TJ, Gomez-Arboledas A, Tenner AJ. Complement as a powerful influencer in the brain during development, adulthood and neurological disorders. Alt FW, Murphy KM, editors. Adv Immunol. 2021;152:157–222. 10.1016/bs.ai.2021.09.003 34844709

[99] Harcha PA, Garcés P, Arredondo C, Fernández G, Sáez JC, van Zundert B. Mast cell and astrocyte hemichannels and their role in alzheimer’s disease, ALS, and harmful stress conditions. Int J Mol Sci. 2021;22:1924. 33672031 10.3390/ijms22041924 PMC7919494

[100] Feinberg I. Schizophrenia: caused by a fault in programmed synaptic elimination during adolescence? J Psychiatr Res. 1982;17:319–34. 7187776 10.1016/0022-3956(82)90038-3

[101] Schafer DP, Lehrman EK, Kautzman AG, Koyama R, Mardinly AR, Yamasaki R, et al. Microglia sculpt postnatal neural circuits in an activity and complement-dependent manner. Neuron. 2012;74:691–705. 22632727 10.1016/j.neuron.2012.03.026 PMC3528177

[102] Schafer DP, Lehrman EK, Stevens B. The quad-partite synapse: microglia-synapse interactions in the developing and mature CNS. Glia. 2013;61:24–36. 22829357 10.1002/glia.22389 PMC4082974

[103] Crehan H, Hardy J, Pocock J. Blockage of CR1 prevents activation of rodent microglia. Neurobiol Dis. 2013;54:139–49. 23454195 10.1016/j.nbd.2013.02.003

[104] Iram T, Ramirez-Ortiz Z, Byrne MH, Coleman UA, Kingery ND, Means TK, et al. Megf10 is a receptor for C1Q that mediates clearance of apoptotic cells by astrocytes. J Neurosci. 2016;36:5185–92. 27170117 10.1523/JNEUROSCI.3850-15.2016 PMC4863057

[105] Chung W-S, Clarke LE, Wang GX, Stafford BK, Sher A, Chakraborty C, et al. Astrocytes mediate synapse elimination through MEGF10 and MERTK pathways. Nature. 2013;504:394–400. 24270812 10.1038/nature12776 PMC3969024

[106] Chung W-S, Verghese PB, Chakraborty C, Joung J, Hyman BT, Ulrich JD, et al. Novel allele-dependent role for APOE in controlling the rate of synapse pruning by astrocytes. Proc Natl Acad Sci U S A. 2016;113:10186–91. 27559087 10.1073/pnas.1609896113 PMC5018780

[107] Dejanovic B, Wu T, Tsai M-C, Graykowski D, Gandham VD, Rose CM, et al. Complement C1q-dependent excitatory and inhibitory synapse elimination by astrocytes and microglia in alzheimer’s disease mouse models. Nat Aging. 2022;2:837–50. 37118504 10.1038/s43587-022-00281-1 PMC10154216

[108] Brott BK, Raissi AJ, Micheva KD, Vielmetter J, Mendes MS, Baccus CJ, et al. C4d, a high-affinity LilrB2 ligand, is elevated in alzheimer’s disease and mediates synapse pruning. Proc Natl Acad Sci U S A. 2025;122:e2519253122. 40966293 10.1073/pnas.2519253122 PMC12478167

[109] Welsh CA, Stephany C-É, Sapp RW, Stevens B. Ocular dominance plasticity in binocular primary visual cortex does not require C1q. J Neurosci. 2020;40:769–83. 31801811 10.1523/JNEUROSCI.1011-19.2019 PMC6975301

[110] Kraus DM, Elliott GS, Chute H, Horan T, Pfenninger KH, Sanford SD, et al. CSMD1 is a novel multiple domain complement-regulatory protein highly expressed in the central nervous system and epithelial tissues. J Immunol. 2006;176:4419–30. 16547280 10.4049/jimmunol.176.7.4419

[111] Schartz ND, Liang HY, Carvalho K, Chu S-H, Mendoza-Arvilla A, Petrisko TJ, et al. C5aR1 antagonism suppresses inflammatory glial responses and alters cellular signaling in an alzheimer’s disease mouse model. Nat Commun. 2024;15:7028. 39147742 10.1038/s41467-024-51163-6 PMC11327341

[112] Byrne RAJ, Nimmo J, Torvell M, Carpanini SM, Daskoulidou N, Hughes TR, et al. The schizophrenia-associated gene CSMD1 encodes a complement classical pathway inhibitor predominantly expressed by astrocytes and at synapses in mice and humans. Brain Behav Immun. 2025;127:287–302. 40112933 10.1016/j.bbi.2025.03.026

[113] Shimizu A, Asakawa S, Sasaki T, Yamazaki S, Yamagata H, Kudoh J, et al. A novel giant gene CSMD3 encoding a protein with CUB and Sushi multiple domains: a candidate gene for benign adult Familial myoclonic epilepsy on human chromosome 8q23.3-q24.1. Biochem Biophys Res Commun. 2003;309:143–54. 12943675 10.1016/s0006-291x(03)01555-9

[114] Song W, Li Q, Wang T, Li Y, Fan T, Zhang J, et al. Putative complement control protein CSMD3 dysfunction impairs synaptogenesis and induces neurodevelopmental disorders. Brain Behav Immun. 2022;102:237–50. 35245678 10.1016/j.bbi.2022.02.027

[115] Tu Z, Cohen M, Bu H, Lin F. Tissue distribution and functional analysis of Sushi domain-containing protein 4. Am J Pathol. 2010;176:2378–84. 20348246 10.2353/ajpath.2010.091036 PMC2861102

[116] Holmquist E, Okroj M, Nodin B, Jirström K, Blom AM. Sushi domain-containing protein 4 (SUSD4) inhibits complement by disrupting the formation of the classical C3 convertase. FASEB J. 2013;27:2355–66. 23482636 10.1096/fj.12-222042

[117] Royer B, Soares DC, Barlow PN, Bontrop RE, Roll P, Robaglia-Schlupp A, et al. Molecular evolution of the human SRPX2 gene that causes brain disorders of the Rolandic and Sylvian speech areas. BMC Genet. 2007;8:72. 17942002 10.1186/1471-2156-8-72 PMC2151080

[118] Cong Q, Soteros BM, Wollet M, Kim JH, Sia G-M. The endogenous neuronal complement inhibitor SRPX2 protects against complement-mediated synapse elimination during development. Nat Neurosci. 2020;23:1067–78. 32661396 10.1038/s41593-020-0672-0 PMC7483802

[119] Cong Q, Soteros BM, Huo A, Li Y, Tenner AJ, Sia GM. C1q and SRPX2 regulate microglia mediated synapse elimination during early development in the visual thalamus but not the visual cortex. Glia. 2022;70:451–65. 34762332 10.1002/glia.24114 PMC8732326

[120] Brown AS. Epidemiologic studies of exposure to prenatal infection and risk of schizophrenia and autism. Dev Neurobiol. 2012;72:1272–6. 22488761 10.1002/dneu.22024 PMC3435457

[121] Sekar A, Bialas AR, de Rivera H, Davis A, Hammond TR, Kamitaki N, et al. Schizophrenia risk from complex variation of complement component 4. Nature. 2016;530:177–83. 26814963 10.1038/nature16549 PMC4752392

[122] Yilmaz M, Yalcin E, Presumey J, Aw E, Ma M, Whelan CW, et al. Overexpression of schizophrenia susceptibility factor human complement C4A promotes excessive synaptic loss and behavioral changes in mice. Nat Neurosci. 2021;24:214–24. 33353966 10.1038/s41593-020-00763-8 PMC8086435

[123] Ponath G, Lincoln MR, Levine-Ritterman M, Park C, Dahlawi S, Mubarak M, et al. Enhanced astrocyte responses are driven by a genetic risk allele associated with multiple sclerosis. Nat Commun. 2018;9:5337. 30559390 10.1038/s41467-018-07785-8 PMC6297228

[124] Fitzgerald KC, Kim K, Smith MD, Aston SA, Fioravante N, Rothman AM, et al. Early complement genes are associated with visual system degeneration in multiple sclerosis. Brain. 2019;142:2722–36. 31289819 10.1093/brain/awz188 PMC6776113

[125] Werneburg S, Jung J, Kunjamma RB, Ha S-K, Luciano NJ, Willis CM, et al. Targeted complement Inhibition at synapses prevents microglial synaptic engulfment and synapse loss in demyelinating disease. Immunity. 2020;52:167–e827. 31883839 10.1016/j.immuni.2019.12.004 PMC6996144

[126] Krukowski K, Chou A, Feng X, Tiret B, Paladini M-S, Riparip L-K, et al. Traumatic brain injury in aged mice induces chronic microglia activation, synapse loss, and complement-dependent memory deficits. Int J Mol Sci. 2018;19:3753. 30486287 10.3390/ijms19123753 PMC6321529

[127] Siegel JS, Ramsey LE, Snyder AZ, Metcalf NV, Chacko RV, Weinberger K, et al. Disruptions of network connectivity predict impairment in multiple behavioral domains after stroke. Proc Natl Acad Sci U S A. 2016;113:E4367–76. 27402738 10.1073/pnas.1521083113 PMC4968743

[128] Sun Y, Yin Q, Fang R, Yan X, Wang Y, Bezerianos A, et al. Disrupted functional brain connectivity and its association to structural connectivity in amnestic mild cognitive impairment and alzheimer’s disease. PLoS ONE. 2014;9:e96505. 24806295 10.1371/journal.pone.0096505 PMC4013022

[129] Nardone S, Sams DS, Reuveni E, Getselter D, Oron O, Karpuj M, et al. DNA methylation analysis of the autistic brain reveals multiple dysregulated biological pathways. Transl Psychiatry. 2014;4:e433. 25180572 10.1038/tp.2014.70 PMC4203003

[130] Mansur F, Teles E, Silva AL, Gomes AKS, Magdalon J, de Souza JS, Griesi-Oliveira K, et al. Complement C4 is reduced in iPSC-derived astrocytes of autism spectrum disorder subjects. Int J Mol Sci. 2021;22:7579. 34299197 10.3390/ijms22147579 PMC8305914

[131] Rahpeymai Y, Hietala MA, Wilhelmsson U, Fotheringham A, Davies I, Nilsson A-K, et al. Complement: a novel factor in basal and ischemia-induced neurogenesis. EMBO J. 2006;25:1364–74. 16498410 10.1038/sj.emboj.7601004 PMC1422160

[132] Westacott LJ, Haan N, Evison C, Marei O, Hall J, Hughes TR, et al. Dissociable effects of complement C3 and C3aR on survival and morphology of adult born hippocampal neurons, pattern separation, and cognitive flexibility in male mice. Brain Behav Immun. 2021;98:136–50. 34403734 10.1016/j.bbi.2021.08.215

[133] Michailidou I, Willems JGP, Kooi E-J, van Eden C, Gold SM, Geurts JJG, et al. Complement C1q-C3-associated synaptic changes in multiple sclerosis hippocampus: complement and synapses in MS. Ann Neurol. 2015;77:1007–26. 25727254 10.1002/ana.24398

[134] Watkins LM, Neal JW, Loveless S, Michailidou I, Ramaglia V, Rees MI, et al. Complement is activated in progressive multiple sclerosis cortical grey matter lesions. J Neuroinflammation. 2016;13:161. 27333900 10.1186/s12974-016-0611-x PMC4918026

[135] Alawieh A, Langley EF, Tomlinson S. Targeted complement Inhibition salvages stressed neurons and inhibits neuroinflammation after stroke in mice. Sci Transl Med. 2018;10:eaao6459. 29769288 10.1126/scitranslmed.aao6459 PMC6689196

[136] Stahel PF, Morganti-Kossmann MC, Kossmann T. The role of the complement system in traumatic brain injury. Brain Res Brain Res Rev. 1998;27:243–56. 9729408 10.1016/s0165-0173(98)00015-0

[137] Fluiter K, Opperhuizen AL, Morgan BP, Baas F, Ramaglia V. Inhibition of the membrane attack complex of the complement system reduces secondary neuroaxonal loss and promotes neurologic recovery after traumatic brain injury in mice. J Immunol. 2014;192:2339–48. 24489093 10.4049/jimmunol.1302793

[138] Bathini P, Schilling S, Lemere CA. Anti-Amyloid immunotherapy for AD: A potential link between ARIA and complement. Alzheimer’s Dement. 2025;20:e093183.

[139] van Olst L, Simonton B, Edwards AJ, Forsyth AV, Boles J, Jamshidi P, et al. Microglial mechanisms drive amyloid-β clearance in immunized patients with alzheimer’s disease. Nat Med. 2025;31:1604–16. 40050704 10.1038/s41591-025-03574-1 PMC12092304

[140] Boon BDC, Piura YD, Moloney CM, Chalk JL, Lincoln SJ, Rutledge MH, et al. Neuropathological changes and amyloid-related imaging abnormalities in alzheimer’s disease treated with aducanumab versus untreated: a retrospective case-control study. Lancet Neurol. 2025;24:931–44. 41109234 10.1016/S1474-4422(25)00313-8 PMC12550255

[141] Ueno M, Chiba Y, Murakami R, Matsumoto K, Kawauchi M, Fujihara R. Blood-brain barrier and blood-cerebrospinal fluid barrier in normal and pathological conditions. Brain Tumor Pathol. 2016;33:89–96. 26920424 10.1007/s10014-016-0255-7

[142] Segarra M, Aburto MR, Acker-Palmer A. Blood-brain barrier dynamics to maintain brain homeostasis. Trends Neurosci. 2021;44:393–405. 33423792 10.1016/j.tins.2020.12.002

[143] Huang X, Hussain B, Chang J. Peripheral inflammation and blood-brain barrier disruption: effects and mechanisms. CNS Neurosci Ther. 2021;27:36–47. 33381913 10.1111/cns.13569 PMC7804893

[144] Zlokovic BV. The blood-brain barrier in health and chronic neurodegenerative disorders. Neuron. 2008;57:178–201. 18215617 10.1016/j.neuron.2008.01.003

[145] del Zoppo GJ, Mabuchi T. Cerebral microvessel responses to focal ischemia. J Cereb Blood Flow Metab. 2003;23:879–94. 12902832 10.1097/01.WCB.0000078322.96027.78

[146] Kaur C, Ling EA. Blood brain barrier in hypoxic-ischemic conditions. Curr Neurovasc Res. 2008;5:71–81. 18289024 10.2174/156720208783565645

[147] Lee JD, Coulthard LG, Woodruff TM. Complement dysregulation in the central nervous system during development and disease. Semin Immunol. 2019;45:101340. 31708347 10.1016/j.smim.2019.101340

[148] Erickson MA, Banks WA. Blood-brain barrier dysfunction as a cause and consequence of alzheimer’s disease. J Cereb Blood Flow Metab. 2013;33:1500–13. 23921899 10.1038/jcbfm.2013.135 PMC3790938

[149] Jacob A, Alexander JJ. Complement and blood-brain barrier integrity. Mol Immunol. 2014;61:149–52. 25041699 10.1016/j.molimm.2014.06.039

[150] Alexander JJ. Blood-brain barrier (BBB) and the complement landscape. Mol Immunol. 2018;102:26–31. 30007547 10.1016/j.molimm.2018.06.267

[151] Iadecola C, Buckwalter MS, Anrather J. Immune responses to stroke: mechanisms, modulation, and therapeutic potential. J Clin Invest. 2020;130:2777–88. 32391806 10.1172/JCI135530 PMC7260029

[152] Brandl S, Reindl M. Blood-Brain barrier breakdown in neuroinflammation: current in vitro models. Int J Mol Sci. 2023;24(16):12699. 37628879 10.3390/ijms241612699 PMC10454051

[153] Delvenne A, Vandendriessche C, Gobom J, Burgelman M, Dujardin P, De Nolf C, et al. Involvement of the choroid plexus in alzheimer’s disease pathophysiology: findings from mouse and human proteomic studies. Fluids Barriers CNS. 2024;21:58. 39020361 10.1186/s12987-024-00555-3 PMC11256635

[154] Eikelenboom P, Stam FC. Immunoglobulins and complement factors in senile plaques. An immunoperoxidase study. Acta Neuropathol. 1982;57:239–42. 6812382 10.1007/BF00685397

[155] Ishii T, Haga S. Immuno-electron-microscopic localization of complements in amyloid fibrils of senile plaques. Acta Neuropathol. 1984;63:296–300. 6382906 10.1007/BF00687336

[156] McGeer PL, Rogers J. Anti-inflammatory agents as a therapeutic approach to alzheimer’s disease. Neurology. 1992;42:447–9. 1736183 10.1212/wnl.42.2.447

[157] McGeer PL, McGeer EG. The inflammatory response system of brain: implications for therapy of alzheimer and other neurodegenerative diseases. Brain Res Brain Res Rev. 1995;21:195–218. 8866675 10.1016/0165-0173(95)00011-9

[158] McGeer PL, Akiyama H, Itagaki S, McGeer EG. Activation of the classical complement pathway in brain tissue of alzheimer patients. Neurosci Lett. 1989;107:341–6. 2559373 10.1016/0304-3940(89)90843-4

[159] Afagh A, Cummings BJ, Cribbs DH, Cotman CW, Tenner AJ. Localization and cell association of C1q in alzheimer’s disease brain. Exp Neurol. 1996;138:22–32. 8593893 10.1006/exnr.1996.0043

[160] Veerhuis R, van der Valk P, Janssen I, Zhan SS, Van Nostrand WE, Eikelenboom P. Complement activation in amyloid plaques in alzheimer’s disease brains does not proceed further than C3. Virchows Arch. 1995;426:603–10. 7655742 10.1007/BF00192116

[161] Veerhuis R, Janssen I, Hack CE, Eikelenboom P. Early complement components in alzheimer’s disease brains. Acta Neuropathol. 1996;91:53–60. 8773146 10.1007/s004019570001

[162] Itagaki S, Akiyama H, Saito H, McGeer PL. Ultrastructural localization of complement membrane attack complex (MAC)-like immunoreactivity in brains of patients with alzheimer’s disease. Brain Res. 1994;645:78–84. 8062101 10.1016/0006-8993(94)91640-3

[163] Webster S, Lue LF, Brachova L, Tenner AJ, McGeer PL, Terai K, et al. Molecular and cellular characterization of the membrane attack complex, C5b-9, in alzheimer’s disease. Neurobiol Aging. 1997;18:415–21. 9330973 10.1016/s0197-4580(97)00042-0

[164] Yasojima K, Schwab C, McGeer EG, McGeer PL. Up-regulated production and activation of the complement system in alzheimer’s disease brain. Am J Pathol. 1999;154:927–36. 10079271 10.1016/S0002-9440(10)65340-0 PMC1866427

[165] Shen Y, Meri S. Yin and yang: complement activation and regulation in alzheimer’s disease. Prog Neurobiol. 2003;70:463–72. 14568360 10.1016/j.pneurobio.2003.08.001

[166] Akiyama H, Barger S, Barnum S, Bradt B, Bauer J, Cole GM, et al. Inflammation and alzheimer’s disease. Neurobiol Aging. 2000;21:383–421. 10858586 10.1016/s0197-4580(00)00124-x PMC3887148

[167] Tenner AJ. Complement in alzheimer’s disease: opportunities for modulating protective and pathogenic events. Neurobiol Aging. 2001;22:849–61. 11754992 10.1016/s0197-4580(01)00301-3

[168] Stoltzner SE, Grenfell TJ, Mori C, Wisniewski KE, Wisniewski TM, Selkoe DJ, et al. Temporal accrual of complement proteins in amyloid plaques in down’s syndrome with alzheimer’s disease. Am J Pathol. 2000;156:489–99. 10666378 10.1016/S0002-9440(10)64753-0 PMC1850044

[169] Webster S, Bonnell B, Rogers J. Charge-based binding of complement component C1q to the alzheimer amyloid beta-peptide. Am J Pathol. 1997;150:1531–6. 9137079 PMC1858209

[170] Webster SD, Yang AJ, Margol L, Garzon-Rodriguez W, Glabe CG, Tenner AJ. Complement component C1q modulates the phagocytosis of Abeta by microglia. Exp Neurol. 2000;161:127–38. 10683279 10.1006/exnr.1999.7260

[171] Tacnet-Delorme P, Chevallier S, Arlaud GJ. Beta-amyloid fibrils activate the C1 complex of complement under physiological conditions: evidence for a binding site for A beta on the C1q globular regions. J Immunol. 2001;167:6374–81. 11714802 10.4049/jimmunol.167.11.6374

[172] Shen Y, Lue L, Yang L, Roher A, Kuo Y, Strohmeyer R, et al. Complement activation by neurofibrillary tangles in alzheimer’s disease. Neurosci Lett. 2001;305:165–8. 11403931 10.1016/s0304-3940(01)01842-0

[173] Tanskanen M, Lindsberg PJ, Tienari PJ, Polvikoski T, Sulkava R, Verkkoniemi A, et al. Cerebral amyloid angiopathy in a 95 + cohort: complement activation and Apolipoprotein E (ApoE) genotype. Neuropathol Appl Neurobiol. 2005;31:589–99. 16281907 10.1111/j.1365-2990.2005.00652.x

[174] Rogers J, Li R, Mastroeni D, Grover A, Leonard B, Ahern G, et al. Peripheral clearance of amyloid beta peptide by complement C3-dependent adherence to erythrocytes. Neurobiol Aging. 2006;27:1733–9. 16290270 10.1016/j.neurobiolaging.2005.09.043

[175] Wang Y, Hancock AM, Bradner J, Chung KA, Quinn JF, Peskind ER, et al. Complement 3 and factor h in human cerebrospinal fluid in parkinson’s disease, alzheimer’s disease, and multiple-system atrophy. Am J Pathol. 2011;178:1509–16. 21435440 10.1016/j.ajpath.2011.01.006 PMC3078443

[176] Daborg J, Andreasson U, Pekna M, Lautner R, Hanse E, Minthon L, et al. Cerebrospinal fluid levels of complement proteins C3, C4 and CR1 in alzheimer’s disease. J Neural Transm (Vienna). 2012;119:789–97. 22488444 10.1007/s00702-012-0797-8

[177] Daskoulidou N, Shaw B, Torvell M, Watkins L, Cope EL, Carpanini SM, et al. Complement receptor 1 is expressed on brain cells and in the human brain. Glia. 2023;71:1522–35. 36825534 10.1002/glia.24355 PMC10953339

[178] Daskoulidou N, Shaw B, Zelek WM, Morgan BP. The alzheimer’s disease-associated complement receptor 1 variant confers risk by impacting glial phagocytosis. Alzheimers Dement. 2025;21:e70458. 40631443 10.1002/alz.70458 PMC12238831

[179] Wu T, Dejanovic B, Gandham VD, Gogineni A, Edmonds R, Schauer S, et al. Complement C3 is activated in human AD brain and is required for neurodegeneration in mouse models of amyloidosis and tauopathy. Cell Rep. 2019;28:2111–e236. 31433986 10.1016/j.celrep.2019.07.060

[180] Stephan AH, Madison DV, Mateos JM, Fraser DA, Lovelett EA, Coutellier L, et al. A dramatic increase of C1q protein in the CNS during normal aging. J Neurosci. 2013;33:13460–74. 23946404 10.1523/JNEUROSCI.1333-13.2013 PMC3742932

[181] Dejanovic B, Huntley MA, De Mazière A, Meilandt WJ, Wu T, Srinivasan K, et al. Changes in the synaptic proteome in tauopathy and rescue of Tau-induced synapse loss by C1q antibodies. Neuron. 2018;100:1322–e367. 30392797 10.1016/j.neuron.2018.10.014

[182] Yin C, Ackermann S, Ma Z, Mohanta SK, Zhang C, Li Y, et al. ApoE attenuates unresolvable inflammation by complex formation with activated C1q. Nat Med. 2019;25:496–506. 30692699 10.1038/s41591-018-0336-8 PMC6420126

[183] Bonham LW, Desikan RS, Yokoyama JS, Alzheimer’s Disease Neuroimaging Initiative. The relationship between complement factor C3, APOE ε4, amyloid and Tau in alzheimer’s disease. Acta Neuropathol Commun. 2016;4:65. 27357286 10.1186/s40478-016-0339-y PMC4928261

[184] Chatterjee M, Özdemir S, Kunadt M, Koel-Simmelink M, Boiten W, Piepkorn L, et al. C1q is increased in cerebrospinal fluid-derived extracellular vesicles in alzheimer’s disease: A multi-cohort proteomics and immuno-assay validation study. Alzheimers Dement. 2023;19:4828–40. 37023079 10.1002/alz.13066

[185] Attems J, Jellinger K, Thal DR, Van Nostrand W. Review: sporadic cerebral amyloid angiopathy. Neuropathol Appl Neurobiol. 2011;37:75–93. 20946241 10.1111/j.1365-2990.2010.01137.x

[186] De Reuck J. The impact of cerebral amyloid angiopathy in various neurodegenerative dementia syndromes: A neuropathological study. Neurol Res Int. 2019;2019:7247325. 30792924 10.1155/2019/7247325 PMC6354160

[187] Premkumar DR, Cohen DL, Hedera P, Friedland RP, Kalaria RN. Apolipoprotein E-epsilon4 alleles in cerebral amyloid angiopathy and cerebrovascular pathology associated with alzheimer’s disease. Am J Pathol. 1996;148:2083–95. 8669492 PMC1861657

[188] Charidimou A, Gang Q, Werring DJ. Sporadic cerebral amyloid angiopathy revisited: recent insights into pathophysiology and clinical spectrum. J Neurol Neurosurg Psychiatry. 2012;83:124–37. 22056963 10.1136/jnnp-2011-301308

[189] Ullah R, Lee EJ. Advances in Amyloid-β clearance in the brain and periphery: implications for neurodegenerative diseases. Exp Neurobiol. 2023;32:216–46. 37749925 10.5607/en23014 PMC10569141

[190] Bruce SS, Zhang C, Liberman AL, Merkler AE, Navi BB, Chiang GC, Iadecola C, Kamel H, Murthy SB. Prevalence of cerebral amyloid angiopathy and associated risk of subsequent ischemic and hemorrhagic stroke and mortality in a nationwide cohort. Ann Neurol. 2025;98(2):249–57. 40309957 10.1002/ana.27253 PMC12629278

[191] Matsuo K, Shindo A, Niwa A, Tabei K-I, Akatsu H, Hashizume Y, et al. Complement activation in capillary cerebral amyloid angiopathy. Dement Geriatr Cogn Disord. 2017;44:343–53. 29421784 10.1159/000486091

[192] Fan R, DeFilippis K, Van Nostrand WE. Induction of complement proteins in a mouse model for cerebral microvascular Aβ deposition. J Neuroinflamm. 2007;4:1–8. 10.1186/1742-2094-4-22 PMC2099424 17877807

[193] Verbeek MM, Otte-Höller I, Veerhuis R, Ruiter DJ, De Waal RM. Distribution of A beta-associated proteins in cerebrovascular amyloid of alzheimer’s disease. Acta Neuropathol. 1998;96:628–36. 9845293 10.1007/s004010050944

[194] Hondius DC, Eigenhuis KN, van der Morrema THJ, van Nierop P, Bugiani M, et al. Proteomics analysis identifies new markers associated with capillary cerebral amyloid angiopathy in alzheimer’s disease. Acta Neuropathol Commun. 2018;6:1–19. 29860944 10.1186/s40478-018-0540-2 PMC5985582

[195] Manousopoulou A, Gatherer M, Smith C, Nicoll JAR, Woelk CH, Johnson M, et al. Systems proteomic analysis reveals that clusterin and tissue inhibitor of metalloproteinases 3 increase in leptomeningeal arteries affected by cerebral amyloid angiopathy. Neuropathol Appl Neurobiol. 2017;43:492–504. 27543695 10.1111/nan.12342 PMC5638106

[196] Endo Y, Hasegawa K, Nomura R, Arishima H, Kikuta K-I, Yamashita T, et al. Apolipoprotein E and clusterin inhibit the early phase of amyloid-β aggregation in an in vitro model of cerebral amyloid angiopathy. Acta Neuropathol Commun. 2019;7:12. 30691533 10.1186/s40478-019-0662-1 PMC6348632

[197] Zellner A, Müller SA, Lindner B, Beaufort N, Rozemuller AJM, Arzberger T, et al. Proteomic profiling in cerebral amyloid angiopathy reveals an overlap with CADASIL highlighting accumulation of HTRA1 and its substrates. Acta Neuropathol Commun. 2022;10:6. 35074002 10.1186/s40478-021-01303-6 PMC8785498

[198] Leitner D, Kavanagh T, Kanshin E, Balcomb K, Pires G, Thierry M, et al. Differences in the cerebral amyloid angiopathy proteome in alzheimer’s disease and mild cognitive impairment. Acta Neuropathol. 2024;148:1–20. 39039355 10.1007/s00401-024-02767-1 PMC11263258

[199] Varma C, Lemere CA. CAA proteomics meta-analysis reveals novel targets, key players, and the effects of sex, APOE, and brain region in humans. Acta Neuropathol. 2025;149:40. 40299065 10.1007/s00401-025-02886-3 PMC12041165

[200] Hong S, Beja-Glasser VF, Nfonoyim BM, Frouin A, Li S, Ramakrishnan S, et al. Complement and microglia mediate early synapse loss in alzheimer mouse models. Science. 2016;352:712–6. 27033548 10.1126/science.aad8373 PMC5094372

[201] Benoit ME, Hernandez MX, Dinh ML, Benavente F, Vasquez O, Tenner AJ. C1q-induced LRP1B and GPR6 proteins expressed early in alzheimer disease mouse models, are essential for the C1q-mediated protection against amyloid-β neurotoxicity. J Biol Chem. 2013;288:654–65. 23150673 10.1074/jbc.M112.400168 PMC3537064

[202] Fonseca MI, Zhou J, Botto M, Tenner AJ. Absence of C1q leads to less neuropathology in Transgenic mouse models of alzheimer’s disease. J Neurosci. 2004;24:6457–65. 15269255 10.1523/JNEUROSCI.0901-04.2004 PMC6729885

[203] Zhou J, Fonseca MI, Pisalyaput K, Tenner AJ. Complement C3 and C4 expression in C1q sufficient and deficient mouse models of alzheimer’s disease. J Neurochem. 2008;106:2080–92. 18624920 10.1111/j.1471-4159.2008.05558.x PMC2574638

[204] Wang Y, Wu T, Gogineni A, Tsai M-C, Kielpinski L, Mahajan A, et al. Therapeutically targeting the classical complement pathway with antisense oligonucleotides in alzheimer’s disease. BioRxiv. 2024. 2024.08.23.609240.

[205] Fonseca MI, Chu S-H, Hernandez MX, Fang MJ, Modarresi L, Selvan P, et al. Cell-specific deletion of C1qa identifies microglia as the dominant source of C1q in mouse brain. J Neuroinflammation. 2017;14:48. 28264694 10.1186/s12974-017-0814-9 PMC5340039

[206] Petrisko TJ, Gargus M, Chu S-H, Selvan P, Whiteson KL, Tenner AJ. Influence of complement protein C1q or complement receptor C5aR1 on gut microbiota composition in wildtype and alzheimer’s mouse models. J Neuroinflammation. 2023;20:211. 37726739 10.1186/s12974-023-02885-9 PMC10507976

[207] Bie B, Wu J, Foss JF, Naguib M. Activation of mGluR1 mediates C1q-dependent microglial phagocytosis of glutamatergic synapses in alzheimer’s rodent models. Mol Neurobiol. 2019;56:5568–85. 30652266 10.1007/s12035-019-1467-8 PMC6615956

[208] Spurrier J, Nicholson L, Fang XT, Stoner AJ, Toyonaga T, Holden D, et al. Reversal of synapse loss in alzheimer mouse models by targeting mGluR5 to prevent synaptic tagging by C1Q. Sci Transl Med. 2022;14:eabi8593. 35648810 10.1126/scitranslmed.abi8593 PMC9554345

[209] Györffy BA, Tóth V, Török G, Gulyássy P, Kovács RÁ, Vadászi H, et al. Synaptic mitochondrial dysfunction and Septin accumulation are linked to complement-mediated synapse loss in an alzheimer’s disease animal model. Cell Mol Life Sci. 2020;77:5243–58. 32034429 10.1007/s00018-020-03468-0 PMC7671981

[210] Hao X, Li Z, Li W, Katz J, Michalek SM, Barnum SR, et al. Periodontal infection aggravates C1q-mediated microglial activation and synapse pruning in alzheimer’s mice. Front Immunol. 2022;13:816640. 35178049 10.3389/fimmu.2022.816640 PMC8845011

[211] Zhou J, Wade SD, Graykowski D, Xiao M-F, Zhao B, Giannini LAA, et al. The neuronal pentraxin Nptx2 regulates complement activity and restrains microglia-mediated synapse loss in neurodegeneration. Sci Transl Med. 2023;15:eadf0141. 36989373 10.1126/scitranslmed.adf0141 PMC10467038

[212] Zhong L, Sheng X, Wang W, Li Y, Zhuo R, Wang K, et al. TREM2 receptor protects against complement-mediated synaptic loss by binding to complement C1q during neurodegeneration. Immunity. 2023;56:1794–e8088. 37442133 10.1016/j.immuni.2023.06.016

[213] Lin D, Kaye S, Chen M, Lyanna A, Ye L, Hammond LA, et al. Transcriptome and proteome profiling reveals TREM2-dependent and -independent glial response and metabolic perturbation in an alzheimer’s mouse model. J Biol Chem. 2024;300:107874. 39395805 10.1016/j.jbc.2024.107874 PMC11570940

[214] Haure-Mirande J-V, Wang M, Audrain M, Fanutza T, Kim SH, Heja S, et al. Integrative approach to sporadic alzheimer’s disease: deficiency of TYROBP in cerebral Aβ amyloidosis mouse normalizes clinical phenotype and complement subnetwork molecular pathology without reducing Aβ burden. Mol Psychiatry. 2019;24:431–46. 30283032 10.1038/s41380-018-0255-6 PMC6494440

[215] Audrain M, Haure-Mirande J-V, Wang M, Kim SH, Fanutza T, Chakrabarty P, et al. Integrative approach to sporadic alzheimer’s disease: deficiency of TYROBP in a Tauopathy mouse model reduces C1q and normalizes clinical phenotype while increasing spread and state of phosphorylation of Tau. Mol Psychiatry. 2019;24:1383–97. 30283031 10.1038/s41380-018-0258-3 PMC6447470

[216] Zhang C, Qi H, Jia D, Zhao J, Xu C, Liu J, et al. Cognitive impairment in alzheimer’s disease FAD4T mouse model: synaptic loss facilitated by activated microglia via C1qA. Life Sci. 2024;340:122457. 38266812 10.1016/j.lfs.2024.122457

[217] Zhang L, Huang L, Zhou Y, Meng J, Zhang L, Zhou Y, et al. Microglial CD2AP deficiency exerts protection in an alzheimer’s disease model of amyloidosis. Mol Neurodegener. 2024;19:95. 39695808 10.1186/s13024-024-00789-7 PMC11658232

[218] Li S, Li M, Li G, Li L, Yang X, Zuo Z et al. Physical exercise decreases complement-mediated synaptic loss and protects against cognitive impairment by inhibiting microglial Tmem9-ATP6V0D1 in alzheimer’s disease. Aging Cell. 2025;e14496. 10.1111/acel.14496 PMC12073899 39871402

[219] Shi Q, Colodner KJ, Matousek SB, Merry K, Hong S, Kenison JE, et al. Complement C3-deficient mice fail to display age-related hippocampal decline. J Neurosci. 2015;35:13029–42. 26400934 10.1523/JNEUROSCI.1698-15.2015 PMC6605437

[220] Shi Q, Chowdhury S, Ma R, Le KX, Hong S, Caldarone BJ, Stevens B, Lemere CA. Complement C3 deficiency protects against neurodegeneration in aged plaque-rich APP/PS1 mice. Sci Transl Med. 2017;9(392):eaaf6295. 28566429 10.1126/scitranslmed.aaf6295 PMC6936623

[221] Carpanini SM, Torvell M, Bevan RJ, Byrne RAJ, Daskoulidou N, Saito T, et al. Terminal complement pathway activation drives synaptic loss in alzheimer’s disease models. Acta Neuropathol Commun. 2022;10:99. 35794654 10.1186/s40478-022-01404-w PMC9258209

[222] Litvinchuk A, Wan Y-W, Swartzlander DB, Chen F, Cole A, Propson NE, et al. Complement C3aR inactivation attenuates Tau pathology and reverses an immune network deregulated in Tauopathy models and alzheimer’s disease. Neuron. 2018;100:1337–e535. 30415998 10.1016/j.neuron.2018.10.031 PMC6309202

[223] Gedam M, Comerota MM, Propson NE, Chen T, Jin F, Wang MC, Zheng H. Complement C3aR depletion reverses HIF-1α-induced metabolic impairment and enhances microglial response to Aβ pathology. J Clin Invest. 2023;133(12):e167501. 37317973 10.1172/JCI167501 PMC10266793

[224] Wang Y, Pandey S, Weber M, Choy MK, Wu T, Chernov-Rogan T, et al. Complement C3aR deletion does not attenuate neurodegeneration in a tauopathy model or alter acute inflammation-induced gene expression changes in the brain. bioRxiv. 2025. p. 2025.06.16.660026. 10.1016/j.celrep.2026.117313 42033726

[225] Propson NE, Roy ER, Litvinchuk A, Köhl J, Zheng H. Endothelial C3a receptor mediates vascular inflammation and blood-brain barrier permeability during aging. J Clin Invest. 2021;131(1):e140966. 32990682 10.1172/JCI140966 PMC7773352

[226] Bhatia K, Kindelin A, Nadeem M, Khan MB, Yin J, Fuentes A, et al. Complement C3a receptor (C3aR) mediates vascular dysfunction, hippocampal pathology, and cognitive impairment in a mouse model of VCID. Transl Stroke Res. 2022;13:816–29. 35258803 10.1007/s12975-022-00993-x

[227] Yao Y, Chang Y, Li S, Zhu J, Wu Y, Jiang X, et al. Complement C3a receptor antagonist alleviates Tau pathology and ameliorates cognitive deficits in P301S mice. Brain Res Bull. 2023;200:110685. 37330021 10.1016/j.brainresbull.2023.110685

[228] Woodruff TM, Tenner AJ. A commentary on: NFκB-activated astroglial release of complement C3 compromises neuronal morphology and function associated with alzheimer’s disease. A cautionary note regarding C3aR. Front Immunol. 2015;6:220. 25999955 10.3389/fimmu.2015.00220 PMC4423305

[229] Lee JD, Taylor SM, Woodruff TM. Is the C3a receptor antagonist SB290157 a useful Pharmacological tool? Br J Pharmacol. 2020;177:5677–8. 33073351 10.1111/bph.15264 PMC7707084

[230] Britschgi M, Takeda-Uchimura Y, Rockenstein E, Johns H, Masliah E, Wyss-Coray T. Deficiency of terminal complement pathway inhibitor promotes neuronal Tau pathology and degeneration in mice. J Neuroinflammation. 2012;9:220. 22989354 10.1186/1742-2094-9-220 PMC3511294

[231] Zhu X-C, Liu L, Dai W-Z, Ma T. Crry Silencing alleviates alzheimer’s disease injury by regulating neuroinflammatory cytokines and the complement system. Neural Regen Res. 2022;17:1841–9. 35017447 10.4103/1673-5374.332160 PMC8820699

[232] Hasantari I, Nicolas N, Alzieu P, Leval L, Shalabi A, Grolleau S, et al. Factor h’s control of complement activation emerges as a significant and promising therapeutic target for alzheimer’s disease treatment. Int J Mol Sci. 2024;25:2272. 38396950 10.3390/ijms25042272 PMC10889136

[233] Ding X, Wang J, Huang M, Chen Z, Liu J, Zhang Q, et al. Loss of microglial SIRPα promotes synaptic pruning in preclinical models of neurodegeneration. Nat Commun. 2021;12:2030. 33795678 10.1038/s41467-021-22301-1 PMC8016980

[234] Merlini M, Rafalski VA, Rios Coronado PE, Gill TM, Ellisman M, Muthukumar G, et al. Fibrinogen induces microglia-mediated spine elimination and cognitive impairment in an alzheimer’s disease model. Neuron. 2019;101:1099–e1086. 30737131 10.1016/j.neuron.2019.01.014 PMC6602536

[235] Shi X, Ohta Y, Liu X, Shang J, Morihara R, Nakano Y, et al. Chronic cerebral hypoperfusion activates the coagulation and complement cascades in alzheimer’s disease mice. Neuroscience. 2019;416:126–36. 31394196 10.1016/j.neuroscience.2019.07.050

[236] Yoo CJ, Choi Y, Bok E, Lin Y, Cheon M, Lee Y-H, et al. Complement receptor 4 mediates the clearance of extracellular Tau fibrils by microglia. FEBS J. 2024;291:3499–520. 38715400 10.1111/febs.17150

[237] Fonseca MI, Ager RR, Chu S-H, Yazan O, Sanderson SD, LaFerla FM, et al. Treatment with a C5aR antagonist decreases pathology and enhances behavioral performance in murine models of alzheimer’s disease. J Immunol. 2009;183:1375–83. 19561098 10.4049/jimmunol.0901005 PMC4067320

[238] Landlinger C, Oberleitner L, Gruber P, Noiges B, Yatsyk K, Santic R, et al. Active immunization against complement factor C5a: a new therapeutic approach for alzheimer’s disease. J Neuroinflammation. 2015;12:150. 26275910 10.1186/s12974-015-0369-6 PMC4537556

[239] Alshammari AM, Smith DD, Parriott J, Stewart JP, Curran SM, McCulloh RJ, et al. Targeted amino acid substitution overcomes scale-up challenges with the human C5a-derived decapeptide immunostimulant EP67. ACS Infect Dis. 2020;6:1169–81. 32233506 10.1021/acsinfecdis.0c00005 PMC7279522

[240] Panayiotou E, Fella E, Andreou S, Papacharalambous R, Gerasimou P, Costeas P, et al. C5aR agonist enhances phagocytosis of fibrillar and non-fibrillar Aβ amyloid and preserves memory in a mouse model of Familial alzheimer’s disease. PLoS ONE. 2019;14:e0225417. 31809505 10.1371/journal.pone.0225417 PMC6897413

[241] Li XX, Clark RJ, Woodruff TM. Anaphylatoxin receptor promiscuity for commonly used complement C5a peptide agonists. Int Immunopharmacol. 2021;100:108074. 34454293 10.1016/j.intimp.2021.108074

[242] Vogen SM, Paczkowski NJ, Kirnarsky L, Short A, Whitmore JB, Sherman SA, et al. Differential activities of decapeptide agonists of human C5a: the conformational effects of backbone N-methylation. Int Immunopharmacol. 2001;1:2151–62. 11710544 10.1016/s1567-5769(01)00141-2

[243] Carvalho K, Schartz ND, Balderrama-Gutierrez G, Liang HY, Chu S-H, Selvan P, et al. Modulation of C5a-C5aR1 signaling alters the dynamics of AD progression. J Neuroinflammation. 2022;19:178. 35820938 10.1186/s12974-022-02539-2 PMC9277945

[244] Gomez-Arboledas A, Carvalho K, Balderrama-Gutierrez G, Chu S-H, Liang HY, Schartz ND, et al. C5aR1 antagonism alters microglial polarization and mitigates disease progression in a mouse model of alzheimer’s disease. Acta Neuropathol Commun. 2022;10:116. 35978440 10.1186/s40478-022-01416-6 PMC9386996

[245] Gomez-Arboledas A, Fonseca MI, Kramar E, Chu S-H, Schartz ND, Selvan P, et al. C5aR1 signaling promotes region- and age-dependent synaptic pruning in models of alzheimer’s disease. Alzheimers Dement. 2024;20:2173–90. 38278523 10.1002/alz.13682 PMC10984438

[246] Zelek WM, Bevan RJ, Morgan BP. Targeting terminal pathway reduces brain complement activation, amyloid load and synapse loss, and improves cognition in a mouse model of dementia. Brain Behav Immun. 2024;118:355–63. 38485063 10.1016/j.bbi.2024.03.017

[247] Zelek WM, Bevan RJ, Nimmo J, Dewilde M, De Strooper B, Morgan BP. Brain-penetrant complement Inhibition mitigates neurodegeneration in an alzheimer’s disease mouse model. Brain. 2025;148:941–54. 39215579 10.1093/brain/awae278 PMC11884734

[248] Hu M, Li T, Ma X, Liu S, Li C, Huang Z, et al. Macrophage lineage cells-derived migrasomes activate complement-dependent blood-brain barrier damage in cerebral amyloid angiopathy mouse model. Nat Commun. 2023;14:3945. 37402721 10.1038/s41467-023-39693-x PMC10319857

[249] Chen W-T, Lu A, Craessaerts K, Pavie B, Sala Frigerio C, Corthout N, et al. Spatial transcriptomics and in situ sequencing to study alzheimer’s disease. Cell. 2020;182:976–e9119. 32702314 10.1016/j.cell.2020.06.038

[250] Fonseca MI, Chu S-H, Berci AM, Benoit ME, Peters DG, Kimura Y, et al. Contribution of complement activation pathways to neuropathology differs among mouse models of alzheimer’s disease. J Neuroinflammation. 2011;8:4. 21235806 10.1186/1742-2094-8-4 PMC3033336

[251] Maier M, Peng Y, Jiang L, Seabrook TJ, Carroll MC, Lemere CA. Complement C3 deficiency leads to accelerated amyloid plaque deposition and neurodegeneration and modulation of the microglia/macrophage phenotype in amyloid precursor protein Transgenic mice. J Neurosci. 2008;28:6333–41. 18562603 10.1523/JNEUROSCI.0829-08.2008 PMC3329761

[252] Zhuang B, Mancarci BO, Toker L, Pavlidis P. Mega-analysis of gene expression in mouse models of Alzheimer’s disease. eNeuro. 2019;6:ENEURO.0226–19.2019. 10.1523/ENEURO.0226-19.2019 PMC6893236 31767574

[253] Zhang X, Liu W, Cao Y, Tan W. Hippocampus proteomics and brain lipidomics reveal network dysfunction and lipid molecular abnormalities in APP/PS1 mouse model of alzheimer’s disease. J Proteome Res. 2020;19:3427–37. 32510958 10.1021/acs.jproteome.0c00255

[254] Lee MJ, Wang C, Carroll MJ, Brubaker DK, Hyman BT, Lauffenburger DA. Computational interspecies translation between alzheimer’s disease mouse models and human subjects identifies innate immune complement, TYROBP, and TAM receptor agonist signatures, distinct from influences of aging. Front Neurosci. 2021;15:727784. 34658769 10.3389/fnins.2021.727784 PMC8515135

[255] Varma C, Luo E, Bostrom G, Bathini P, Berdnik D, Wyss-Coray T, et al. Plasma and CSF biomarkers of aging and cognitive decline in Caribbean vervets. Alzheimers Dement. 2024;20:5460–80. 38946666 10.1002/alz.14038 PMC11350037

[256] Liddelow SA, Guttenplan KA, Clarke LE, Bennett FC, Bohlen CJ, Schirmer L, et al. Neurotoxic reactive astrocytes are induced by activated microglia. Nature. 2017;541:481–7. 28099414 10.1038/nature21029 PMC5404890

[257] Lian H, Yang L, Cole A, Sun L, Chiang AC-A, Fowler SW, et al. NFκB-activated astroglial release of complement C3 compromises neuronal morphology and function associated with alzheimer’s disease. Neuron. 2015;85:101–15. 25533482 10.1016/j.neuron.2014.11.018 PMC4289109

[258] Lian H, Litvinchuk A, Chiang AC-A, Aithmitti N, Jankowsky JL, Zheng H. Astrocyte-microglia cross talk through complement activation modulates amyloid pathology in mouse models of alzheimer’s disease. J Neurosci. 2016;36:577–89. 26758846 10.1523/JNEUROSCI.2117-15.2016 PMC4710776

[259] Guttikonda SR, Sikkema L, Tchieu J, Saurat N, Walsh RM, Harschnitz O, et al. Fully defined human pluripotent stem cell-derived microglia and tri-culture system model C3 production in alzheimer’s disease. Nat Neurosci. 2021;24:343–54. 33558694 10.1038/s41593-020-00796-z PMC8382543

[260] Iram T, Trudler D, Kain D, Kanner S, Galron R, Vassar R, et al. Astrocytes from old alzheimer’s disease mice are impaired in Aβ uptake and in neuroprotection. Neurobiol Dis. 2016;96:84–94. 27544484 10.1016/j.nbd.2016.08.001

[261] De Schepper S, Ge JZ, Crowley G, Ferreira LSS, Garceau D, Toomey CE, et al. Perivascular cells induce microglial phagocytic States and synaptic engulfment via SPP1 in mouse models of alzheimer’s disease. Nat Neurosci. 2023;26:406–15. 36747024 10.1038/s41593-023-01257-z PMC9991912

[262] Abud EM, Ramirez RN, Martinez ES, Healy LM, Nguyen CHH, Newman SA, et al. IPSC-derived human microglia-like cells to study neurological diseases. Neuron. 2017;94:278–e939. 28426964 10.1016/j.neuron.2017.03.042 PMC5482419

[263] Penney J, Ralvenius WT, Loon A, Cerit O, Dileep V, Milo B, et al. iPSC-derived microglia carrying the TREM2 R47H/+ mutation are Proinflammatory and promote synapse loss. Glia. 2024;72:452–69. 37969043 10.1002/glia.24485 PMC10904109

[264] Reich M, Paris I, Ebeling M, Dahm N, Schweitzer C, Reinhardt D, et al. Alzheimer’s risk gene TREM2 determines functional properties of new type of human iPSC-derived microglia. Front Immunol. 2020;11:617860. 33613545 10.3389/fimmu.2020.617860 PMC7887311

[265] Gomes C, Huang K-C, Harkin J, Baker A, Hughes JM, Pan Y, et al. Induction of astrocyte reactivity promotes neurodegeneration in human pluripotent stem cell models. Stem Cell Rep. 2024;19:1122–36. 10.1016/j.stemcr.2024.07.002 PMC11368677 39094561

[266] Dräger NM, Sattler SM, Huang CT-L, Teter OM, Leng K, Hashemi SH, et al. A CRISPRi/a platform in human iPSC-derived microglia uncovers regulators of disease States. Nat Neurosci. 2022;25:1149–62. 35953545 10.1038/s41593-022-01131-4 PMC9448678

[267] Thielen AJF, van Baarsen IM, Jongsma ML, Zeerleder S, Spaapen RM, Wouters D. CRISPR/Cas9 generated human CD46, CD55 and CD59 knockout cell lines as a tool for complement research. J Immunol Methods. 2018;456:15–22. 29447841 10.1016/j.jim.2018.02.004

[268] Wenzel TJ, Desjarlais JD, Mousseau DD. Human brain organoids containing microglia that have arisen innately adapt to a β-amyloid challenge better than those in which microglia are integrated by co-culture. Stem Cell Res Ther. 2024;15:258. 39135132 10.1186/s13287-024-03876-0 PMC11320858

[269] Chen X, Sun G, Feng L, Tian E, Shi Y. Human iPSC-derived microglial cells protect neurons from neurodegeneration in long-term cultured adhesion brain organoids. Commun Biol. 2025;8:30. 39789340 10.1038/s42003-024-07401-0 PMC11718079

[270] Urrestizala-Arenaza N, Cerchio S, Cavaliere F, Magliaro C. Limitations of human brain organoids to study neurodegenerative diseases: a manual to survive. Front Cell Neurosci. 2024;18:1419526. 39049825 10.3389/fncel.2024.1419526 PMC11267621

[271] Pașca SP. The rise of three-dimensional human brain cultures. Nature. 2018;553:437–45. 29364288 10.1038/nature25032

[272] Zhou-Yang L, Eichhorner S, Karbacher L, Böhnke L, Traxler L, Mertens J. Direct conversion of human fibroblasts to induced neurons. Methods Mol Biol. 2021;2352:73–96. 34324181 10.1007/978-1-0716-1601-7_6

[273] Cakir B, Tanaka Y, Kiral FR, Xiang Y, Dagliyan O, Wang J, et al. Expression of the transcription factor PU.1 induces the generation of microglia-like cells in human cortical organoids. Nat Commun. 2022;13:430. 35058453 10.1038/s41467-022-28043-y PMC8776770

[274] Mathys H, Davila-Velderrain J, Peng Z, Gao F, Mohammadi S, Young JZ, et al. Single-cell transcriptomic analysis of alzheimer’s disease. Nature. 2019;570:332–7. 31042697 10.1038/s41586-019-1195-2 PMC6865822

[275] Kemper C, Köhl J. Back to the future - non-canonical functions of complement. Semin Immunol. 2018;37:1–3. 29857931 10.1016/j.smim.2018.05.002 PMC7470017

[276] Denk S, Neher MD, Messerer DAC, Wiegner R, Nilsson B, Rittirsch D, et al. Complement C5a functions as a master switch for the pH balance in neutrophils exerting fundamental immunometabolic effects. J Immunol. 2017;198:4846–54. 28490576 10.4049/jimmunol.1700393

[277] Arbore G, West EE, Spolski R, Robertson AAB, Klos A, Rheinheimer C, et al. T helper 1 immunity requires complement-driven NLRP3 inflammasome activity in CD4^+^ T cells. Science. 2016;352:aad1210. 27313051 10.1126/science.aad1210 PMC5015487

[278] Maffia P, Mauro C, Case A, Kemper C. Canonical and non-canonical roles of complement in atherosclerosis. Nat Rev Cardiol. 2024;21:743–61. 38600367 10.1038/s41569-024-01016-y

[279] de Oliveira-Tore F, de Moraes C, Plácido AG, Signorini HMBS, Fontana NMDL, Batista Godoy PD, et al. Non-canonical extracellular complement pathways and the complosome paradigm in cancer: a scoping review. Front Immunol. 2025;16:1519465. 10.3389/fimmu.2025.1519465 PMC12075386 40370471

[280] Pittaluga A, Torre V, Olivero G, Rosenwasser N, Taddeucci A. Non-canonical roles of complement in the CNS: from synaptic organizer to presynaptic modulator of glutamate transmission. Curr Neuropharmacol. 2025;23:820–34. 39817397 10.2174/011570159X327960240823065729 PMC12163473

[281] West EE, Kemper C. Intracellular C1q - an unexpected player in neuronal proteostasis. Trends Immunol. 2024;45:718–20. 39327206 10.1016/j.it.2024.09.006 PMC12163964

[282] Farrer LA. Effects of age, sex, and ethnicity on the association between Apolipoprotein E genotype and alzheimer disease: A meta-analysis. JAMA. 1997;278:1349. 9343467

[283] Lambert J-C, Amouyel P. Genetics of alzheimer’s disease: new evidences for an old hypothesis? Curr Opin Genet Dev. 2011;21:295–301. 21371880 10.1016/j.gde.2011.02.002

[284] Li H, Wetten S, Li L, St Jean PL, Upmanyu R, Surh L, et al. Candidate single-nucleotide polymorphisms from a genomewide association study of alzheimer disease. Arch Neurol. 2008;65:45–53. 17998437 10.1001/archneurol.2007.3

[285] Reiman EM, Webster JA, Myers AJ, Hardy J, Dunckley T, Zismann VL, et al. GAB2 alleles modify alzheimer’s risk in APOE ɛ4 carriers. Neuron. 2007;54:713–20. 17553421 10.1016/j.neuron.2007.05.022 PMC2587162

[286] Abraham R, Moskvina V, Sims R, Hollingworth P, Morgan A, Georgieva L, et al. A genome-wide association study for late-onset alzheimer’s disease using DNA pooling. BMC Med Genomics. 2008;1:44. 18823527 10.1186/1755-8794-1-44 PMC2570675

[287] Bertram L, Lange C, Mullin K, Parkinson M, Hsiao M, Hogan MF, et al. Genome-wide association analysis reveals putative alzheimer’s disease susceptibility loci in addition to APOE. Am J Hum Genet. 2008;83:623–32. 18976728 10.1016/j.ajhg.2008.10.008 PMC2668052

[288] Carrasquillo MM, Zou F, Pankratz VS, Wilcox SL, Ma L, Walker LP, et al. Genetic variation in PCDH11X is associated with susceptibility to late-onset alzheimer’s disease. Nat Genet. 2009;41:192–8. 19136949 10.1038/ng.305 PMC2873177

[289] Beecham GW, Martin ER, Li Y-J, Slifer MA, Gilbert JR, Haines JL, et al. Genome-wide association study implicates a chromosome 12 risk locus for late-onset alzheimer disease. Am J Hum Genet. 2009;84:35–43. 19118814 10.1016/j.ajhg.2008.12.008 PMC2668056

[290] Bertram L, Tanzi RE. Alzheimer disease: new light on an old CLU: alzheimer disease. Nat Rev Neurol. 2010;6:11–3. 20057494 10.1038/nrneurol.2009.213 PMC2860389

[291] de Silva HV, Harmony JA, Stuart WD, Gil CM, Robbins J. Apolipoprotein J: structure and tissue distribution. Biochemistry. 1990;29:5380–9. 1974459 10.1021/bi00474a025

[292] DeMattos RB, Cirrito JR, Parsadanian M, May PC, O’Dell MA, Taylor JW, et al. ApoE and clusterin cooperatively suppress Abeta levels and deposition: evidence that ApoE regulates extracellular Abeta metabolism in vivo. Neuron. 2004;41:193–202. 14741101 10.1016/s0896-6273(03)00850-x

[293] Jones SE, Jomary C, Clusterin. Int J Biochem Cell Biol. 2002;34:427–31. 11906815 10.1016/s1357-2725(01)00155-8

[294] Nuutinen T, Suuronen T, Kauppinen A, Salminen A. Clusterin: a forgotten player in alzheimer’s disease. Brain Res Rev. 2009;61:89–104. 19651157 10.1016/j.brainresrev.2009.05.007

[295] Wojtas AM, Sens JP, Kang SS, Baker KE, Berry TJ, Kurti A, et al. Astrocyte-derived clusterin suppresses amyloid formation in vivo. Mol Neurodegener. 2020;15:71. 33246484 10.1186/s13024-020-00416-1 PMC7694353

[296] Lish AM, Grogan EFL, Benoit CR, Pearse RV 2nd, Heuer SE, Luquez T, et al. CLU alleviates alzheimer’s disease-relevant processes by modulating astrocyte reactivity and microglia-dependent synaptic density. Neuron. 2025;113(12):1925–e194611. 10.1016/j.neuron.2025.03.034 PMC12181066 40311610

[297] Yuste-Checa P, Trinkaus VA, Riera-Tur I, Imamoglu R, Schaller TF, Wang H, et al. The extracellular chaperone clusterin enhances Tau aggregate seeding in a cellular model. Nat Commun. 2021;12:4863. 34381050 10.1038/s41467-021-25060-1 PMC8357826

[298] DeMattos RB, O’dell MA, Parsadanian M, Taylor JW, Harmony JAK, Bales KR, et al. Clusterin promotes amyloid plaque formation and is critical for neuritic toxicity in a mouse model of alzheimer’s disease. Proc Natl Acad Sci U S A. 2002;99:10843–8. 12145324 10.1073/pnas.162228299 PMC125060

[299] Yerbury JJ, Poon S, Meehan S, Thompson B, Kumita JR, Dobson CM, et al. The extracellular chaperone clusterin influences amyloid formation and toxicity by interacting with prefibrillar structures. FASEB J. 2007;21:2312–22. 17412999 10.1096/fj.06-7986com

[300] Szymanski M, Wang R, Bassett SS, Avramopoulos D. Alzheimer’s risk variants in the clusterin gene are associated with alternative splicing. Transl Psychiatry. 2011;1:e18–18. 21892414 10.1038/tp.2011.17 PMC3165035

[301] Allen M, Zou F, Chai HS, Younkin CS, Crook J, Pankratz VS, et al. Novel late-onset alzheimer disease loci variants associate with brain gene expression. Neurology. 2012;79:221–8. 22722634 10.1212/WNL.0b013e3182605801 PMC3398432

[302] Antúnez C, Boada M, López-Arrieta J, Moreno-Rey C, Hernández I, Marín J, et al. Genetic association of complement receptor 1 polymorphism rs3818361 in alzheimer’s disease. Alzheimers Dement. 2011;7:e124–9. 21784344 10.1016/j.jalz.2011.05.2412

[303] Li Y, Song D, Jiang Y, Wang J, Feng R, Zhang L, et al. CR1 rs3818361 polymorphism contributes to alzheimer’s disease susceptibility in Chinese population. Mol Neurobiol. 2016;53:4054–9. 26189835 10.1007/s12035-015-9343-7

[304] Van Cauwenberghe C, Bettens K, Engelborghs S, Vandenbulcke M, Van Dongen J, Vermeulen S, et al. Complement receptor 1 coding variant p.Ser1610Thr in alzheimer’s disease and related endophenotypes. Neurobiol Aging. 2013;34:e22351–6. 10.1016/j.neurobiolaging.2013.03.008 23582656

[305] Shen N, Chen B, Jiang Y, Feng R, Liao M, Zhang L, et al. An updated analysis with 85,939 samples confirms the association between CR1 rs6656401 polymorphism and alzheimer’s disease. Mol Neurobiol. 2015;51:1017–23. 24878768 10.1007/s12035-014-8761-2

[306] Jin C, Li W, Yuan J, Xu W, Cheng Z. Association of the CR1 polymorphism with late-onset alzheimer’s disease in Chinese Han populations: a meta-analysis. Neurosci Lett. 2012;527:46–9. 22960360 10.1016/j.neulet.2012.08.032

[307] Zhang Q, Yu J-T, Zhu Q-X, Zhang W, Wu Z-C, Miao D, et al. Complement receptor 1 polymorphisms and risk of late-onset alzheimer’s disease. Brain Res. 2010;1348:216–21. 20558149 10.1016/j.brainres.2010.06.018

[308] Ma X-Y, Yu J-T, Tan M-S, Sun F-R, Miao D, Tan L. Missense variants in CR1 are associated with increased risk of alzheimer’ disease in Han Chinese. Neurobiol Aging. 2014;35:e44317–21. 10.1016/j.neurobiolaging.2013.08.009 24018213

[309] Kucukkilic E, Brookes K, Barber I, Guetta-Baranes T, Consortium ARUK, Morgan K, et al. Complement receptor 1 gene (CR1) intragenic duplication and risk of alzheimer’s disease. Hum Genet. 2018;137:305–14. 29675612 10.1007/s00439-018-1883-2 PMC5937907

[310] Hazrati L-N, Van Cauwenberghe C, Brooks PL, Brouwers N, Ghani M, Sato C, et al. Genetic association of CR1 with alzheimer’s disease: a tentative disease mechanism. Neurobiol Aging. 2012;33:e29495–294912. 10.1016/j.neurobiolaging.2012.07.001 22819390

[311] Crehan H, Holton P, Wray S, Pocock J, Guerreiro R, Hardy J. Complement receptor 1 (CR1) and alzheimer’s disease. Immunobiology. 2012;217:244–50. 21840620 10.1016/j.imbio.2011.07.017

[312] Chibnik LB, Shulman JM, Leurgans SE, Schneider JA, Wilson RS, Tran D, et al. CR1 is associated with amyloid plaque burden and age-related cognitive decline. Ann Neurol. 2011;69:560–9. 21391232 10.1002/ana.22277 PMC3066288

[313] Biffi A, Shulman JM, Jagiella JM, Cortellini L, Ayres AM, Schwab K, et al. Genetic variation at CR1 increases risk of cerebral amyloid angiopathy. Neurology. 2012;78:334–41. 22262751 10.1212/WNL.0b013e3182452b40 PMC3280047

[314] Hamilton G, Evans KL, Macintyre DJ, Deary IJ, Dominiczak A, Smith BH, et al. Alzheimer’s disease risk factor complement receptor 1 is associated with depression. Neurosci Lett. 2012;510:6–9. 22244847 10.1016/j.neulet.2011.12.059

[315] Fonseca MI, Chu S, Pierce AL, Brubaker WD, Hauhart RE, Mastroeni D, et al. Analysis of the putative role of CR1 in alzheimer’s disease: genetic association, expression and function. PLoS ONE. 2016;11:e0149792. 26914463 10.1371/journal.pone.0149792 PMC4767815

[316] Keenan BT, Shulman JM, Chibnik LB, Raj T, Tran D, Sabuncu MR, et al. A coding variant in CR1 interacts with APOE-ɛ4 to influence cognitive decline. Hum Mol Genet. 2012;21:2377–88. 22343410 10.1093/hmg/dds054 PMC3335317

[317] Lazaris A, Hwang KS, Goukasian N, Ramirez LM, Eastman J, Blanken AE, et al. Alzheimer risk genes modulate the relationship between plasma ApoE and cortical PiB binding. Neurol Genet. 2015;1:e22. 27066559 10.1212/NXG.0000000000000022 PMC4809461

[318] Raychaudhuri S, Iartchouk O, Chin K, Tan PL, Tai AK, Ripke S, et al. A rare penetrant mutation in CFH confers high risk of age-related macular degeneration. Nat Genet. 2011;43:1232–6. 22019782 10.1038/ng.976 PMC3225644

[319] Zetterberg M, Landgren S, Andersson ME, Palmér MS, Gustafson DR, Skoog I, et al. Association of complement factor H Y402H gene polymorphism with alzheimer’s disease. Am J Med Genet B Neuropsychiatr Genet. 2008;147B:720–6. 18163432 10.1002/ajmg.b.30668

[320] Zhang D-F, Li J, Wu H, Cui Y, Bi R, Zhou H-J, et al. CFH variants affect structural and functional brain changes and genetic risk of alzheimer’s disease. Neuropsychopharmacology. 2016;41:1034–45. 26243271 10.1038/npp.2015.232 PMC4748428

[321] Veteleanu A, Stevenson-Hoare J, Keat S, Daskoulidou N, Zetterberg H, Heslegrave A, et al. Alzheimer’s disease-associated complement gene variants influence plasma complement protein levels. J Neuroinflammation. 2023;20(1):169. 37480051 10.1186/s12974-023-02850-6 PMC10362776

[322] Hatzimanolis A, Foteli S, Stefanatou P, Ntigrintaki A-A, Ralli I, Kollias K, et al. Deregulation of complement components C4A and CSMD1 peripheral expression in first-episode psychosis and links to cognitive ability. Eur Arch Psychiatry Clin Neurosci. 2022;272:1219–28. 35532796 10.1007/s00406-022-01409-5 PMC9508018

[323] Baum ML, Wilton DK, Fox RG, Carey A, Hsu Y-HH, Hu R, et al. CSMD1 regulates brain complement activity and circuit development. Brain Behav Immun. 2024;119:317–32. 38552925 10.1016/j.bbi.2024.03.041

[324] van den Hove DLA, Riemens RJM, Koulousakis P, Pishva E. Epigenome-wide association studies in alzheimer’s disease; achievements and challenges. Brain Pathol. 2020;30:978–83. 32654262 10.1111/bpa.12880 PMC8018126

[325] Xu S-Y, Zhang S, Zeng C-L, Peng Y-J, Xu M. Emerging trends and hot spots in epigenetic modifications in neurology: A bibliometric analysis. Mol Neurobiol. 2025;62(8):10511–29. 40216692 10.1007/s12035-025-04862-0

[326] Mattei AL, Bailly N, Meissner A. DNA methylation: a historical perspective. Trends Genet. 2022;38:676–707. 35504755 10.1016/j.tig.2022.03.010

[327] Lunnon K, Mill J. Epigenetic studies in alzheimer’s disease: current findings, caveats, and considerations for future studies. Am J Med Genet B Neuropsychiatr Genet. 2013;162B:789–99. 24038819 10.1002/ajmg.b.32201 PMC3947441

[328] Lardenoije R, Pishva E, van den Lunnon K. Neuroepigenetics of aging and age-related neurodegenerative disorders. Prog Mol Biol Transl Sci. 2018;158:49–82. 30072060 10.1016/bs.pmbts.2018.04.008

[329] Mitsumori R, Sakaguchi K, Shigemizu D, Mori T, Akiyama S, Ozaki K, et al. Lower DNA methylation levels in CpG Island Shores of CR1, CLU, and PICALM in the blood of Japanese alzheimer’s disease patients. PLoS ONE. 2020;15:e0239196. 32991610 10.1371/journal.pone.0239196 PMC7523949

[330] Chibnik LB, Yu L, Eaton ML, Srivastava G, Schneider JA, Kellis M, et al. Alzheimer’s loci: epigenetic associations and interaction with genetic factors. Ann Clin Transl Neurol. 2015;2:636–47. 26125039 10.1002/acn3.201 PMC4479524

[331] Alfimova M, Kondratyev N, Golov A, Golimbet V. Relationship between alzheimer’s disease-associated SNPs within the CLU gene, local DNA methylation and episodic verbal memory in healthy and schizophrenia subjects. Psychiatry Res. 2019;272:380–6. 30599442 10.1016/j.psychres.2018.12.134

[332] Zhang X-X, Wei M, Wang H-R, Hu Y-Z, Sun H-M, Jia J-J. Mitochondrial dysfunction gene expression, DNA methylation, and inflammatory cytokines interaction activate alzheimer’s disease: a multi-omics Mendelian randomization study. J Transl Med. 2024;22:893. 39363202 10.1186/s12967-024-05680-z PMC11448268

[333] Loison F, Debure L, Nizard P, le Goff P, Michel D, le Dréan Y. Up-regulation of the clusterin gene after proteotoxic stress: implication of HSF1-HSF2 heterocomplexes. Biochem J. 2006;395:223–31. 16336210 10.1042/BJ20051190 PMC1409688

[334] Rauhala HE, Porkka KP, Saramäki OR, Tammela TLJ, Visakorpi T. Clusterin is epigenetically regulated in prostate cancer. Int J Cancer. 2008;123:1601–9. 18649357 10.1002/ijc.23658

[335] Nuutinen T, Suuronen T, Kyrylenko S, Huuskonen J, Salminen A. Induction of clusterin/apoJ expression by histone deacetylase inhibitors in neural cells. Neurochem Int. 2005;47:528–38. 16157419 10.1016/j.neuint.2005.07.007

[336] Suuronen T, Nuutinen T, Ryhänen T, Kaarniranta K, Salminen A. Epigenetic regulation of clusterin/apolipoprotein J expression in retinal pigment epithelial cells. Biochem Biophys Res Commun. 2007;357:397–401. 17420006 10.1016/j.bbrc.2007.03.135

[337] Gasparoni G, Bultmann S, Lutsik P, Kraus TFJ, Sordon S, Vlcek J, et al. DNA methylation analysis on purified neurons and glia dissects age and alzheimer’s disease-specific changes in the human cortex. Epigenetics Chromatin. 2018;11:41. 30045751 10.1186/s13072-018-0211-3 PMC6058387

[338] Zhang L, Young JI, Gomez L, Silva TC, Schmidt MA, Cai J, et al. Sex-specific DNA methylation differences in alzheimer’s disease pathology. Acta Neuropathol Commun. 2021;9:77. 33902726 10.1186/s40478-021-01177-8 PMC8074512

[339] Millán-Zambrano G, Burton A, Bannister AJ, Schneider R. Histone post-translational modifications - cause and consequence of genome function. Nat Rev Genet. 2022;23:563–80. 35338361 10.1038/s41576-022-00468-7

[340] López-Hernández L, Toolan-Kerr P, Bannister AJ, Millán-Zambrano G. Dynamic histone modification patterns coordinating DNA processes. Mol Cell. 2025;85:225–37. 39824165 10.1016/j.molcel.2024.10.034

[341] Gjoneska E, Pfenning AR, Mathys H, Quon G, Kundaje A, Tsai L-H, et al. Conserved epigenomic signals in mice and humans reveal immune basis of alzheimer’s disease. Nature. 2015;518:365–9. 25693568 10.1038/nature14252 PMC4530583

[342] Marzi SJ, Leung SK, Ribarska T, Hannon E, Smith AR, Pishva E, et al. A histone acetylome-wide association study of alzheimer’s disease identifies disease-associated H3K27ac differences in the entorhinal cortex. Nat Neurosci. 2018;21:1618–27. 30349106 10.1038/s41593-018-0253-7

[343] Nott A, Holtman IR, Coufal NG, Schlachetzki JCM, Yu M, Hu R, et al. Brain cell type-specific enhancer-promoter interactome maps and disease-risk association. Science. 2019;366:1134–9. 31727856 10.1126/science.aay0793 PMC7028213

[344] Kierdorf K, Erny D, Goldmann T, Sander V, Schulz C, Perdiguero EG, et al. Microglia emerge from erythromyeloid precursors via Pu.1- and Irf8-dependent pathways. Nat Neurosci. 2013;16:273–80. 23334579 10.1038/nn.3318

[345] Butovsky O, Jedrychowski MP, Moore CS, Cialic R, Lanser AJ, Gabriely G, et al. Identification of a unique TGF-β-dependent molecular and functional signature in microglia. Nat Neurosci. 2014;17:131–43. 24316888 10.1038/nn.3599 PMC4066672

[346] Kim B, Dabin LC, Tate MD, Karahan H, Sharify AD, Acri DJ, et al. Effects of SPI1-mediated transcriptome remodeling on alzheimer’s disease-related phenotypes in mouse models of Aβ amyloidosis. Nat Commun. 2024;15:3996. 38734693 10.1038/s41467-024-48484-x PMC11088624

[347] Esteller M. Non-coding RNAs in human disease. Nat Rev Genet. 2011;12:861–74. 22094949 10.1038/nrg3074

[348] Lukiw WJ, Zhao Y, Cui JG. An NF-kappaB-sensitive micro RNA-146a-mediated inflammatory circuit in alzheimer disease and in stressed human brain cells. J Biol Chem. 2008;283:31315–22. 18801740 10.1074/jbc.M805371200 PMC2581572

[349] Lukiw WJ, Surjyadipta B, Dua P, Alexandrov PN. Common micro RNAs (miRNAs) target complement factor H (CFH) regulation in alzheimer’s disease (AD) and in age-related macular degeneration (AMD). Int J Biochem Mol Biol. 2012;3:105–16. 22509485 PMC3325769

[350] Lukiw WJ, Alexandrov PN. Regulation of complement factor H (CFH) by multiple MiRNAs in alzheimer’s disease (AD) brain. Mol Neurobiol. 2012;46:11–9. 22302353 10.1007/s12035-012-8234-4 PMC3703615

[351] Pogue AI, Li YY, Cui J-G, Zhao Y, Kruck TPA, Percy ME, et al. Characterization of an NF-kappaB-regulated, miRNA-146a-mediated down-regulation of complement factor H (CFH) in metal-sulfate-stressed human brain cells. J Inorg Biochem. 2009;103:1591–5. 19540598 10.1016/j.jinorgbio.2009.05.012

[352] Li YY, Cui JG, Hill JM, Bhattacharjee S, Zhao Y, Lukiw WJ. Increased expression of miRNA-146a in alzheimer’s disease Transgenic mouse models. Neurosci Lett. 2011;487:94–8. 20934487 10.1016/j.neulet.2010.09.079 PMC5382794

[353] Li YY, Cui JG, Dua P, Pogue AI, Bhattacharjee S, Lukiw WJ. Differential expression of miRNA-146a-regulated inflammatory genes in human primary neural, astroglial and microglial cells. Neurosci Lett. 2011;499:109–13. 21640790 10.1016/j.neulet.2011.05.044 PMC3713470

[354] Jaber V, Zhao Y, Lukiw WJ. Alterations in micro RNA-messenger RNA (miRNA-mRNA) coupled signaling networks in sporadic alzheimer’s disease (AD) hippocampal CA1. J Alzheimers Dis Parkinsonism. 2017;7(2):312. 29051843 10.4172/2161-0460.1000312 PMC5645033

[355] Li YY, Alexandrov PN, Pogue AI, Zhao Y, Bhattacharjee S, Lukiw WJ. miRNA-155 upregulation and complement factor H deficits in down’s syndrome. NeuroReport. 2012;23:168–73. 22182977 10.1097/WNR.0b013e32834f4eb4 PMC3264826

[356] Hill JM, Pogue AI, Lukiw WJ. Pathogenic MicroRNAs common to brain and retinal degeneration; recent observations in alzheimer’s disease and age-related macular degeneration. Front Neurol. 2015;6:232. 26579072 10.3389/fneur.2015.00232 PMC4630578

[357] Xu N, Li AD, Ji LL, Ye Y, Wang ZY, Tong L. miR-132 regulates the expression of synaptic proteins in APP/PS1 Transgenic mice through C1q. Eur J Histochem. 2019;63(2):3008. 31060348 10.4081/ejh.2019.3008 PMC6511887

[358] Li A-D, Tong L, Xu N, Ye Y, Nie P-Y, Wang Z-Y, et al. miR-124 regulates cerebromicrovascular function in APP/PS1 Transgenic mice via C1ql3. Brain Res Bull. 2019;153:214–22. 31499089 10.1016/j.brainresbull.2019.09.002

[359] Zhang M, Bian Z. Alzheimer’s disease and microRNA-132: A widespread pathological factor and potential therapeutic target. Front Neurosci. 2021;15:687973. 34108863 10.3389/fnins.2021.687973 PMC8180577

[360] Yoon S, Kim SE, Ko Y, Jeong GH, Lee KH, Lee J, et al. Differential expression of MicroRNAs in alzheimer’s disease: a systematic review and meta-analysis. Mol Psychiatry. 2022;27:2405–13. 35264731 10.1038/s41380-022-01476-z

[361] Sharma M, Pal P, Gupta SK. Deciphering the role of MiRNAs in alzheimer’s disease: predictive targeting and pathway modulation - A systematic review. Ageing Res Rev. 2024;101:102483. 39236856 10.1016/j.arr.2024.102483

[362] Cogswell JP, Ward J, Taylor IA, Waters M, Shi Y, Cannon B, et al. Identification of MiRNA changes in alzheimer’s disease brain and CSF yields putative biomarkers and insights into disease pathways. J Alzheimers Dis. 2008;14:27–41. 18525125 10.3233/jad-2008-14103

[363] Upadhya R, Zingg W, Shetty S, Shetty AK. Astrocyte-derived extracellular vesicles: neuroreparative properties and role in the pathogenesis of neurodegenerative disorders. J Control Release. 2020;323:225–39. 32289328 10.1016/j.jconrel.2020.04.017 PMC7299747

[364] Lukiw WJ, Pogue AI. Vesicular transport of encapsulated MicroRNA between glial and neuronal cells. Int J Mol Sci. 2020;21:5078. 32708414 10.3390/ijms21145078 PMC7404393

[365] Weng S, Lai Q-L, Wang J, Zhuang L, Cheng L, Mo Y, et al. The role of exosomes as mediators of neuroinflammation in the pathogenesis and treatment of alzheimer’s disease. Front Aging Neurosci. 2022;14:899944. 35837481 10.3389/fnagi.2022.899944 PMC9273880

[366] Zhang Y, Zhao Y, Ao X, Yu W, Zhang L, Wang Y, et al. The role of non-coding RNAs in alzheimer’s disease: from regulated mechanism to therapeutic targets and diagnostic biomarkers. Front Aging Neurosci. 2021;13:654978. 34276336 10.3389/fnagi.2021.654978 PMC8283767

[367] Licastro F, Carbone I, Ianni M, Porcellini E. Gene signature in alzheimer’s disease and environmental factors: the virus chronicle. J Alzheimers Dis. 2011;27:809–17. 21891868 10.3233/JAD-2011-110755

[368] Bai B, Wang X, Li Y, Chen P-C, Yu K, Dey KK, et al. Deep multilayer brain proteomics identifies molecular networks in alzheimer’s disease progression. Neuron. 2020;105:975–e917. 31926610 10.1016/j.neuron.2019.12.015 PMC7318843

[369] Pallier PN, Ferrara M, Romagnolo F, Ferretti MT, Soreq H, Cerase A. Chromosomal and environmental contributions to sex differences in the vulnerability to neurological and neuropsychiatric disorders: implications for therapeutic interventions. Prog Neurobiol. 2022;219:102353. 36100191 10.1016/j.pneurobio.2022.102353

[370] Casali BT, Lin L, Benedict O, Zuppe H, Marsico E, Reed EG. Sex chromosomes and gonads modify microglial-mediated pathology in a mouse model of alzheimer’s disease. J Neuroinflammation. 2025;22:81. 40083008 10.1186/s12974-025-03404-8 PMC11907917

[371] Ali M, Garcia P, Lunkes LP, Sciortino A, Thomas M, Heurtaux T, et al. Single cell transcriptome analysis of the THY-Tau22 mouse model of alzheimer’s disease reveals sex-dependent dysregulations. Cell Death Discov. 2024;10:119. 38453894 10.1038/s41420-024-01885-9 PMC10920792

[372] Barber AJ, Del Genio CL, Swain AB, Pizzi EM, Watson SC, Tapiavala VN, et al. Age, sex and alzheimer’s disease: a longitudinal study of 3xTg-AD mice reveals sex-specific disease trajectories and inflammatory responses mirrored in postmortem brains from alzheimer’s patients. Alzheimers Res Ther. 2024;16:134. 38909241 10.1186/s13195-024-01492-x PMC11193202

[373] Davinelli S, Calabrese V, Zella D, Scapagnini G. Epigenetic nutraceutical diets in alzheimer’s disease. J Nutr Health Aging. 2014;18:800–5.25389957 10.1007/s12603-014-0552-y

[374] Mackey-Alfonso SE, Butler MJ, Taylor AM, Williams-Medina AR, Muscat SM, Fu H, et al. Short-term high fat diet impairs memory, exacerbates the neuroimmune response, and evokes synaptic degradation via a complement-dependent mechanism in a mouse model of alzheimer’s disease. Brain Behav Immun. 2024;121:56–69. 39043341 10.1016/j.bbi.2024.07.021 PMC12991061

[375] Poxleitner M, Hoffmann SHL, Berezhnoy G, Ionescu TM, Gonzalez-Menendez I, Maier FC, et al. Western diet increases brain metabolism and adaptive immune responses in a mouse model of amyloidosis. J Neuroinflammation. 2024;21:129. 38745337 10.1186/s12974-024-03080-0 PMC11092112

[376] Barberger-Gateau P, Lambert J-C, Féart C, Pérès K, Ritchie K, Dartigues J-F, et al. From genetics to dietetics: the contribution of epidemiology to Understanding alzheimer’s disease. J Alzheimers Dis. 2013;33(Suppl 1):S457–63. 22683527 10.3233/JAD-2012-129019

[377] De Miguel Z, Khoury N, Betley MJ, Lehallier B, Willoughby D, Olsson N, et al. Exercise plasma boosts memory and dampens brain inflammation via clusterin. Nature. 2021;600:494–9. 34880498 10.1038/s41586-021-04183-x PMC9721468

[378] Yang J, Yuan S, Jian Y, Lei Y, Hu Z, Yang Q, et al. Aerobic exercise regulates GPR81 signal pathway and mediates complement- microglia axis homeostasis on synaptic protection in the early stage of alzheimer’s disease. Life Sci. 2023;331:122042. 37634815 10.1016/j.lfs.2023.122042

[379] Kolonics A, Bori Z, Torma F, Abraham D, Fehér J, Radak Z. Exercise combined with postbiotics treatment results in synergistic improvement of mitochondrial function in the brain of male Transgenic mice for alzheimer’s disease. BMC Neurosci. 2023;24:68. 38110905 10.1186/s12868-023-00836-x PMC10726509

[380] Shatakshi, Dhyani S, Chaurasia H, Kumar H. An overview of our gut microbiome. Probiotics. Boca Raton: CRC; 2024. pp. 29–55.

[381] Kapoor B, Biswas P, Gulati M, Rani P, Gupta R. Gut Microbiome and alzheimer’s disease: what we know and what remains to be explored. Ageing Res Rev. 2024;102:102570. 39486524 10.1016/j.arr.2024.102570

[382] Kulkarni DH, Starick M, Aponte Alburquerque R, Kulkarni HS. Local complement activation and modulation in mucosal immunity. Mucosal Immunol. 2024;17:739–51. 38838816 10.1016/j.mucimm.2024.05.006 PMC11929374

[383] Peterson LW, Artis D. Intestinal epithelial cells: regulators of barrier function and immune homeostasis. Nat Rev Immunol. 2014;14:141–53. 24566914 10.1038/nri3608

[384] Okumura R, Takeda K. Maintenance of intestinal homeostasis by mucosal barriers. Inflamm Regen. 2018;38:5. 29619131 10.1186/s41232-018-0063-z PMC5879757

[385] Han Y, Wang B, Gao H, He C, Hua R, Liang C, et al. Vagus nerve and underlying impact on the gut Microbiota-brain axis in behavior and neurodegenerative diseases. J Inflamm Res. 2022;15:6213–30. 36386584 10.2147/JIR.S384949 PMC9656367

[386] Appleton J. The gut-brain axis: influence of microbiota on mood and mental health. Integr Med (Encinitas). 2018;17:28–32. 31043907 PMC6469458

[387] Arneth BM. Gut–brain axis biochemical signalling from the Gastrointestinal tract to the central nervous system: gut dysbiosis and altered brain function. Postgrad Med J. 2018;94:446–52. 30026389 10.1136/postgradmedj-2017-135424

[388] Chen Y, Chu JM, Wong GT, Chang RC. Complement C3 from astrocytes plays significant roles in sustained activation of microglia and cognitive dysfunctions triggered by systemic inflammation after laparotomy in adult male mice. J Neuroimmune Pharmacol. 2024;19(1):8. 38427092 10.1007/s11481-024-10107-z PMC10907447

[389] Colonna M, Butovsky O. Microglia function in the central nervous system during health and neurodegeneration. Annu Rev Immunol. 2017;35:441–68. 28226226 10.1146/annurev-immunol-051116-052358 PMC8167938

[390] Hrncir T. Gut microbiota dysbiosis: Triggers, consequences, diagnostic and therapeutic options. Microorganisms. 2022;10:578. 35336153 10.3390/microorganisms10030578 PMC8954387

[391] Neurath MF, Artis D, Becker C. The intestinal barrier: a pivotal role in health, inflammation, and cancer. Lancet Gastroenterol Hepatol. 2025;10(6):573–92. 40086468 10.1016/S2468-1253(24)00390-X

[392] Zeng MY, Inohara N, Nuñez G. Mechanisms of inflammation-driven bacterial dysbiosis in the gut. Mucosal Immunol. 2017;10:18–26. 27554295 10.1038/mi.2016.75 PMC5788567

[393] Thakkar A, Vora A, Kaur G, Akhtar J. Dysbiosis and alzheimer’s disease: role of probiotics, prebiotics and synbiotics. Naunyn Schmiedebergs Arch Pharmacol. 2023;396:2911–23. 37284896 10.1007/s00210-023-02554-x

[394] Zou Z, Lei D, Yin Y, Xu R, Luo H, Chen T, et al. Frequent fecal microbiota transplantation improves cognitive impairment and pathological changes in alzheimer’s disease FAD4T mice via the microbiota-gut-brain axis. Heliyon. 2025;11:e42925.

[395] de la Cuesta-Zuluaga J, Kelley ST, Chen Y, Escobar JS, Mueller NT, Ley RE, et al. Age- and Sex-Dependent patterns of gut microbial diversity in human adults. mSystems. 2019;4(4):e00261–19. 31098397 10.1128/mSystems.00261-19 PMC6517691

[396] Upadhyay P, Kumar S, Tyagi A, Tyagi AR, Barbhuyan T, Gupta S. Gut Microbiome rewiring via fecal transplants: Uncovering therapeutic avenues in alzheimer’s disease models. BMC Neurosci. 2025;26(1):39. 40615821 10.1186/s12868-025-00953-9 PMC12231717

[397] Imdad S, Lim W, Kim J-H, Kang C. Intertwined relationship of mitochondrial metabolism, gut Microbiome and exercise potential. Int J Mol Sci. 2022;23:2679. 35269818 10.3390/ijms23052679 PMC8910986

[398] Erny D, Hrabě de Angelis AL, Jaitin D, Wieghofer P, Staszewski O, David E, et al. Host microbiota constantly control maturation and function of microglia in the CNS. Nat Neurosci. 2015;18:965–77. 26030851 10.1038/nn.4030 PMC5528863

[399] Zhang Y, Zhang J, Duan L. The role of microbiota-mitochondria crosstalk in pathogenesis and therapy of intestinal diseases. Pharmacol Res. 2022;186:106530. 36349593 10.1016/j.phrs.2022.106530

[400] Fekete M, Lehoczki A, Tarantini S, Fazekas-Pongor V, Csípő T, Csizmadia Z, Varga JT. Improving cognitive function with nutritional supplements in aging: A comprehensive narrative review of clinical studies investigating the effects of Vitamins, Minerals, Antioxidants, and other dietary supplements. Nutrients. 2023;15(24):5116. 38140375 10.3390/nu15245116 PMC10746024

[401] Jiang Y, Li K, Li X, Xu L, Yang Z. Sodium butyrate ameliorates the impairment of synaptic plasticity by inhibiting the neuroinflammation in 5XFAD mice. Chem Biol Interact. 2021;341:109452. 33785315 10.1016/j.cbi.2021.109452

[402] Shiqing Y, Xinjie L, Xiaotong Z, Jiayan C, Jiahai L, Xiaodong C, et al. Clostridium Butyricum enhances cognitive function in APP/PS1 mice by modulating neuropathology and regulating acetic acid levels in the gut microbiota. Microbiol Spectr. 2025;13:e0017825. 40621927 10.1128/spectrum.00178-25 PMC12323352

[403] Colombo AV, Sadler RK, Llovera G, Singh V, Roth S, Heindl S. Microbiota-derived short chain fatty acids modulate microglia and promote Abeta plaque deposition. 2021. 10.7554/eLife.59826 PMC8043748 33845942

[404] Erny D, Dokalis N, Mezö C, Castoldi A, Mossad O, Staszewski O, et al. Microbiota-derived acetate enables the metabolic fitness of the brain innate immune system during health and disease. Cell Metab. 2021;33:2260–e767. 34731656 10.1016/j.cmet.2021.10.010

[405] Loh JS, Mak WQ, Tan LKS, Ng CX, Chan HH, Yeow SH, et al. Microbiota-gut-brain axis and its therapeutic applications in neurodegenerative diseases. Signal Transduct Target Ther. 2024;9:37. 38360862 10.1038/s41392-024-01743-1 PMC10869798

[406] Noris M, Remuzzi G. Overview of complement activation and regulation. Semin Nephrol. 2013;33:479–92. 24161035 10.1016/j.semnephrol.2013.08.001 PMC3820029

[407] Wu M, Zheng W, Song X, Bao B, Wang Y, Ramanan D, et al. Gut complement induced by the microbiota combats pathogens and spares commensals. Cell. 2024;187:897–e91318. 38280374 10.1016/j.cell.2023.12.036 PMC10922926

[408] Morgan BP. Complement in the pathogenesis of alzheimer’s disease. Semin Immunopathol. 2018;40:113–24. 29134267 10.1007/s00281-017-0662-9 PMC5794825

[409] Vogt NM, Kerby RL, Dill-McFarland KA, Harding SJ, Merluzzi AP, Johnson SC, et al. Gut Microbiome alterations in alzheimer’s disease. Sci Rep. 2017;7:13537. 29051531 10.1038/s41598-017-13601-y PMC5648830

[410] Zhuang Z-Q, Shen L-L, Li W-W, Fu X, Zeng F, Gui L, et al. Gut microbiota is altered in patients with alzheimer’s disease. J Alzheimers Dis. 2018;63:1337–46. 29758946 10.3233/JAD-180176

[411] Saha P, Sisodia SS. Role of the gut Microbiome in mediating sex-specific differences in the pathophysiology of alzheimer’s disease. Neurotherapeutics. 2024;21:e00426. 39054179 10.1016/j.neurot.2024.e00426 PMC11585881

[412] Brandscheid C, Schuck F, Reinhardt S, Schäfer K-H, Pietrzik CU, Grimm M, et al. Altered gut Microbiome composition and tryptic activity of the 5xFAD alzheimer’s mouse model. J Alzheimers Dis. 2017;56:775–88. 28035935 10.3233/JAD-160926

[413] Chen C, Liao J, Xia Y, Liu X, Jones R, Haran J, et al. Gut microbiota regulate alzheimer’s disease pathologies and cognitive disorders via PUFA-associated neuroinflammation. Gut. 2022;71:2233–52. 35017199 10.1136/gutjnl-2021-326269 PMC10720732

[414] Dunham SJB, McNair KA, Adams ED, Avelar-Barragan J, Forner S, Mapstone M, et al. Longitudinal analysis of the Microbiome and metabolome in the 5xfAD mouse model of alzheimer’s disease. MBio. 2022;13:e0179422. 36468884 10.1128/mbio.01794-22 PMC9765021

[415] Liang C, Pereira R, Zhang Y, Rojas OL. Gut Microbiome in alzheimer’s disease: from mice to humans. Curr Neuropharmacol. 2024;22:2314–29. 39403057 10.2174/1570159X22666240308090741 PMC11451315

[416] Tükel C, Wilson RP, Nishimori JH, Pezeshki M, Chromy BA, Bäumler AJ. Responses to amyloids of microbial and host origin are mediated through toll-like receptor 2. Cell Host Microbe. 2009;6:45–53. 19616765 10.1016/j.chom.2009.05.020 PMC2745191

[417] Friedland RP, Chapman MR. The role of microbial amyloid in neurodegeneration. PLoS Pathog. 2017;13:e1006654. 29267402 10.1371/journal.ppat.1006654 PMC5739464

[418] Prosswimmer T, Heng A, Daggett V. Mechanistic insights into the role of amyloid-β in innate immunity. Sci Rep. 2024;14:5376. 38438446 10.1038/s41598-024-55423-9 PMC10912764

[419] Irvine GB, El-Agnaf OM, Shankar GM, Walsh DM. Protein aggregation in the brain: the molecular basis for alzheimer’s and parkinson’s diseases. Mol Med. 2008;14:451–64. 18368143 10.2119/2007-00100.Irvine PMC2274891

[420] DeTure MA, Dickson DW. The neuropathological diagnosis of alzheimer’s disease. Mol Neurodegener. 2019;14:32. 31375134 10.1186/s13024-019-0333-5 PMC6679484

[421] 2025 Alzheimer’s disease facts and figures. Alzheimers Dement. 2025;21. Available from: 10.1002/alz.70235

[422] Gordon BA, Blazey TM, Su Y, Hari-Raj A, Dincer A, Flores S, et al. Spatial patterns of neuroimaging biomarker change in individuals from families with autosomal dominant alzheimer’s disease: a longitudinal study. Lancet Neurol. 2018;17:241–50. 29397305 10.1016/S1474-4422(18)30028-0 PMC5816717

[423] Quiroz YT, Zetterberg H, Reiman EM, Chen Y, Su Y, Fox-Fuller JT, et al. Plasma neurofilament light chain in the presenilin 1 E280A autosomal dominant alzheimer’s disease kindred: a cross-sectional and longitudinal cohort study. Lancet Neurol. 2020;19:513–21. 32470423 10.1016/S1474-4422(20)30137-X PMC7417082

[424] Barthélemy NR, Li Y, Joseph-Mathurin N, Gordon BA, Hassenstab J, Benzinger TLS, et al. A soluble phosphorylated Tau signature links Tau, amyloid and the evolution of stages of dominantly inherited alzheimer’s disease. Nat Med. 2020;26:398–407. 32161412 10.1038/s41591-020-0781-z PMC7309367

[425] Hansson O, Jack CR Jr. A clinical perspective on the revised criteria for diagnosis and staging of alzheimer’s disease. Nat Aging. 2024;4:1029–31. 39009837 10.1038/s43587-024-00675-3

[426] Johnson KA, Fox NC, Sperling RA, Klunk WE. Brain imaging in alzheimer disease. Cold Spring Harb Perspect Med. 2012;2:a006213–006213. 22474610 10.1101/cshperspect.a006213 PMC3312396

[427] Román G, Pascual B. Contribution of neuroimaging to the diagnosis of alzheimer’s disease and vascular dementia. Arch Med Res. 2012;43:671–6. 23142262 10.1016/j.arcmed.2012.10.018

[428] Hampel H, Elhage A, Cummings J, Blennow K, Gao P, Jack CR Jr, et al. The AT(N) system for describing biological changes in alzheimer’s disease: A plain Language summary. Neurodegener Dis Manag. 2022;12:231–9. 35866745 10.2217/nmt-2022-0013

[429] Angioni D, Delrieu J, Hansson O, Fillit H, Aisen P, Cummings J, et al. Blood biomarkers from research use to clinical practice: what must be done? A report from the EU/US CTAD task force. J Prev Alzheimers Dis. 2022;9:569–79. 36281661 10.14283/jpad.2022.85 PMC9683846

[430] Mankhong S, Kim S, Lee S, Kwak H-B, Park D-H, Joa K-L, et al. Development of alzheimer’s disease biomarkers: from CSF- to blood-based biomarkers. Biomedicines. 2022;10:850. 35453600 10.3390/biomedicines10040850 PMC9025524

[431] Shi J, Ou Q, Chen X. Blood-based biomarkers of alzheimer’s disease—A guideline for clinical use. Med Plus. 2024;1:100057.

[432] Cai H, Pang Y, Fu X, Ren Z, Jia L. Plasma biomarkers predict alzheimer’s disease before clinical onset in Chinese cohorts. Nat Commun. 2023;14:6747. 37875471 10.1038/s41467-023-42596-6 PMC10597998

[433] Wouters D, Wiessenberg HD, Hart M, Bruins P, Voskuyl A, Daha MR, et al. Complexes between C1q and C3 or C4: novel and specific markers for classical complement pathway activation. J Immunol Methods. 2005;298:35–45. 15847795 10.1016/j.jim.2004.12.018

[434] Krance SH, Wu C-Y, Zou Y, Mao H, Toufighi S, He X, et al. The complement cascade in alzheimer’s disease: a systematic review and meta-analysis. Mol Psychiatry. 2021;26:5532–41. 31628417 10.1038/s41380-019-0536-8

[435] Strohmeyer R, Shen Y, Rogers J. Detection of complement alternative pathway mRNA and proteins in the alzheimer’s disease brain. Brain Res Mol Brain Res. 2000;81:7–18. 11000474 10.1016/s0169-328x(00)00149-2

[436] Torvell M, Carpanini SM, Daskoulidou N, Byrne RAJ, Sims R, Morgan BP. Genetic insights into the impact of complement in alzheimer’s disease. Genes (Basel). 2021;12(12):1990. 34946939 10.3390/genes12121990 PMC8702080

[437] Hu WT, Watts KD, Tailor P, Nguyen TP, Howell JC, Lee RC, Seyfried NT, Gearing M, Hales CM, Levey AI, Lah JJ, Lee EK. Alzheimer’s disease Neuro-Imaging Initiative. CSF complement 3 and factor H are staging biomarkers in alzheimer’s disease. Acta Neuropathol Commun. 2016;4:14. 26887322 10.1186/s40478-016-0277-8 PMC4758165

[438] Hsu J-L, Lee W-J, Liao Y-C, Wang S-J, Fuh J-L. The clinical significance of plasma clusterin and Aβ in the longitudinal follow-up of patients with alzheimer’s disease. Alzheimers Res Ther. 2017;9:91. 29169407 10.1186/s13195-017-0319-x PMC5701424

[439] Vishnu VY, Modi M, Sharma S, Mohanty M, Goyal MK, Lal V, et al. Role of plasma clusterin in alzheimer’s disease-A pilot study in a tertiary hospital in Northern India. PLoS ONE. 2016;11:e0166369. 27861589 10.1371/journal.pone.0166369 PMC5115728

[440] Hakobyan S, Harding K, Aiyaz M, Hye A, Dobson R, Baird A, et al. Complement biomarkers as predictors of disease progression in alzheimer’s disease. J Alzheimers Dis. 2016;54:707–16. 27567854 10.3233/JAD-160420

[441] Tenner AJ, Stevens B, Woodruff TM. New tricks for an ancient system: physiological and pathological roles of complement in the CNS. Mol Immunol. 2018;102:3–13. 29958698 10.1016/j.molimm.2018.06.264 PMC6478444

[442] Zabel M, Schrag M, Mueller C, Zhou W, Crofton A, Petersen F, et al. Assessing candidate serum biomarkers for alzheimer’s disease: a longitudinal study. J Alzheimers Dis. 2012;30:311–21. 22426016 10.3233/JAD-2012-112012 PMC3616608

[443] Ma Y, Liu Y, Zhang Z, Yang G-Y. Significance of complement system in ischemic stroke: A comprehensive review. Aging Dis. 2019;10:429–62. 31011487 10.14336/AD.2019.0119 PMC6457046

[444] Li Y, Tao C, An N, Liu H, Liu Z, Zhang H, et al. Revisiting the role of the complement system in intracerebral hemorrhage and therapeutic prospects. Int Immunopharmacol. 2023;123:110744. 37552908 10.1016/j.intimp.2023.110744

[445] Zuraw BL, Bork K, Bouillet L, Christiansen SC, Farkas H, Germenis AE, et al. Hereditary angioedema with normal C1 inhibitor: an updated international consensus paper on diagnosis, pathophysiology, and treatment. Clin Rev Allergy Immunol. 2025;68:24. 40053270 10.1007/s12016-025-09027-4 PMC11889046

[446] Mullard A. FDA approves first anti-C1s antibody, targeting innate immunity for rare anaemia. Nat Rev Drug Discovery. 2022;21:249–249. 35277678 10.1038/d41573-022-00049-7

[447] Horneff R, Czech B, Yeh M, Surova E. Three years on: the role of Pegcetacoplan in paroxysmal nocturnal hemoglobinuria (PNH) since its initial approval. Int J Mol Sci. 2024;25:8698. 39201383 10.3390/ijms25168698 PMC11354333

[448] Heier JS, Lad EM, Holz FG, Rosenfeld PJ, Guymer RH, Boyer D, et al. Pegcetacoplan for the treatment of geographic atrophy secondary to age-related macular degeneration (OAKS and DERBY): two multicentre, randomised, double-masked, sham-controlled, phase 3 trials. Lancet. 2023;402:1434–48. 37865470 10.1016/S0140-6736(23)01520-9

[449] Shi JJ, Ozcan YM, Santos CIA, Patel H, Shammo J, Bat T. Current landscape of paroxysmal nocturnal hemoglobinuria in the era of complement inhibitors and regulators. Ther Adv Hematol. 2024;15:20406207241307500. 39734592 10.1177/20406207241307500 PMC11672493

[450] Pardo S, Giovannoni G, Hawkes C, Lechner-Scott J, Waubant E, Levy M. Editorial on: Eculizumab in aquaporin-4-positive neuromyelitis Optica spectrum disorder. Mult Scler Relat Disord. 2019;33:A1–2. 31324299 10.1016/j.msard.2019.07.001

[451] Vu T, Ortiz S, Katsuno M, Annane D, Mantegazza R, Beasley KN, et al. Ravulizumab pharmacokinetics and pharmacodynamics in patients with generalized myasthenia Gravis. J Neurol. 2023;270:3129–37. 36890354 10.1007/s00415-023-11617-1 PMC10188401

[452] McKeage K, Ravulizumab. First global approval. Drugs. 2019;79:347–52. 30767127 10.1007/s40265-019-01068-2

[453] Hoy SM, Pozelimab. First Approval Drugs. 2023;83(16):1551–7. 37856038 10.1007/s40265-023-01955-9

[454] Dhillon S, Crovalimab. First Approval Drugs. 2024;84(6):707–16. 38740735 10.1007/s40265-024-02032-5

[455] Shirley M, Zilucoplan. First Approval Drugs. 2024;84(1):99–104. 38093160 10.1007/s40265-023-01977-3 PMC10925559

[456] Panas RM, Burnett BP, Chong C, Guo R, Riedemann N. Treatment of Viral-induced acute respiratory distress syndrome (ARDS) with vilobelimab: A focus on C5a Inhibition. Am J Respir Crit Care Med. 2025;211(Abstracts):A3110.

[457] Lee A, Avacopan. First Approval Drugs. 2022;82(1):79–85. 34826105 10.1007/s40265-021-01643-6

[458] Syed YY, Iptacopan. First Approval Drugs. 2024;84(5):599–606. 38517653 10.1007/s40265-024-02009-4

[459] Kang C, Danicopan. First Approval Drugs. 2024;84(5):613–8. 38528310 10.1007/s40265-024-02023-6

[460] Latif-Hernandez A, Yang T, Butler RR 3rd, Losada PM, Minhas PS, White H, et al. A TrkB and TrkC partial agonist restores deficits in synaptic function and promotes activity-dependent synaptic and microglial transcriptomic changes in a late-stage alzheimer’s mouse model. Alzheimers Dement. 2024;20:4434–60. 38779814 10.1002/alz.13857 PMC11247716

